# Rare variants with large effects provide functional insights into the pathology of migraine subtypes, with and without aura

**DOI:** 10.1038/s41588-023-01538-0

**Published:** 2023-10-26

**Authors:** Gyda Bjornsdottir, Mona A. Chalmer, Lilja Stefansdottir, Astros Th. Skuladottir, Gudmundur Einarsson, Margret Andresdottir, Doruk Beyter, Egil Ferkingstad, Solveig Gretarsdottir, Bjarni V. Halldorsson, Gisli H. Halldorsson, Anna Helgadottir, Hannes Helgason, Grimur Hjorleifsson Eldjarn, Adalbjorg Jonasdottir, Aslaug Jonasdottir, Ingileif Jonsdottir, Kirk U. Knowlton, Lincoln D. Nadauld, Sigrun H. Lund, Olafur Th. Magnusson, Pall Melsted, Kristjan H. S. Moore, Asmundur Oddsson, Pall I. Olason, Asgeir Sigurdsson, Olafur A. Stefansson, Jona Saemundsdottir, Gardar Sveinbjornsson, Vinicius Tragante, Unnur Unnsteinsdottir, G. Bragi Walters, Florian Zink, Linn Rødevand, Ole A. Andreassen, Jannicke Igland, Rolv T. Lie, Jan Haavik, Karina Banasik, Søren Brunak, Maria Didriksen, Mie T. Bruun, Christian Erikstrup, Lisette J. A. Kogelman, Kaspar R. Nielsen, Erik Sørensen, Ole B. Pedersen, Henrik Ullum, DBDS Genetic Consortium, Jakob Bay, Jens K. Boldsen, Thorsten Brodersen, Kristoffer Burgdorf, Khoa M. Dinh, Joseph Dowsett, Bjarke Feenstra, Frank Geller, Lotte Hindhede, Henrik Hjalgrim, Rikke L. Jacobsen, Gregor Jemec, Katrine Kaspersen, Bertram D. Kjerulf, Margit A. H. Larsen, Ioannis Louloudis, Agnete Lundgaard, Susan Mikkelsen, Christina Mikkelsen, Ioanna Nissen, Mette Nyegaard, Alexander P. Henriksen, Palle D. Rohde, Klaus Rostgaard, Michael Swinn, Lise W. Thørner, Mie T. Bruun, Thomas Werge, David Westergaard, Gisli Masson, Unnur Thorsteinsdottir, Jes Olesen, Petur Ludvigsson, Olafur Thorarensen, Anna Bjornsdottir, Gudrun R. Sigurdardottir, Olafur A. Sveinsson, Sisse R. Ostrowski, Hilma Holm, Daniel F. Gudbjartsson, Gudmar Thorleifsson, Patrick Sulem, Hreinn Stefansson, Thorgeir E. Thorgeirsson, Thomas F. Hansen, Kari Stefansson

**Affiliations:** 1 deCODE Genetics/Amgen, Inc., Reykjavik, Iceland; 2 Danish Headache Center, Department of Neurology, Copenhagen University Hospital, Rigshospitalet-Glostrup, Copenhagen, Denmark; 3 Reykjavik University, School of Technology, Reykjavik, Iceland; 4 School of Engineering and Natural Sciences, University of Iceland, Reykjavik, Iceland; 5 Faculty of Medicine, School of Health Sciences, University of Iceland, Reykjavik, Iceland; 6 Intermountain Heart Institute, Salt Lake City, UT USA; 7 Intermountain Healthcare, Saint George, UT USA; 8 Faculty of Physical Sciences, School of Engineering and Natural Sciences, University of Iceland, Reykjavik, Iceland; 9 NORMENT, Centre for Mental Disorders Research, Division of Mental Health and Addiction, Oslo University Hospital, and Institute of Clinical Medicine, University of Oslo, Oslo, Norway; 10 Department of Global Public Health and Primary Care, University of Bergen, Bergen, Norway; 11 Department of Health and Social Science, Centre for Evidence-Based Practice, Western Norway University of Applied Science, Bergen, Norway; 12 Centre for Fertility and Health, Norwegian Institute of Public Health, Oslo, Norway; 13 Department of Biomedicine, University of Bergen, Bergen, Norway; 14 Division of Psychiatry, Haukeland University Hospital, Bergen, Norway; 15 Novo Nordisk Foundation Center for Protein Research, Faculty of Health and Medical Sciences, University of Copenhagen, Copenhagen, Denmark; 16 Department of Clinical Immunology, Copenhagen University Hospital, Rigshospitalet, Copenhagen, Denmark; 17 Department of Clinical Immunology, Odense University Hospital, Odense, Denmark; 18 Department of Clinical Immunology, Aarhus University Hospital, Aarhus, Denmark; 19 Department of Clinical Medicine Health, Aarhus University, Aarhus, Denmark; 20 Department of Clinical Immunology, Aalborg University Hospital, Aalborg, Denmark; 21 Department of Clinical Medicine, Aalborg University, Aalborg, Denmark; 22 Department of Clinical Immunology, Zealand University Hospital, Køge, Denmark; 23 Department of Clinical Medicine, Faculty of Health and Medical Sciences, University of Copenhagen, Copenhagen, Denmark; 24 Statens Serum Institut, Copenhagen, Denmark; 25 Department of Pediatrics, Landspitali University Hostpital, Reykjavik, Iceland; 26 Heilsuklasinn Clinic, Reykjavik, Iceland; 27 Laeknasetrid Clinic, Reykjavik, Iceland; 28 Department of Neurology, Landspitali University Hospital, Reykjavik, Iceland; 29 Danish Cancer Society Research Center, Copenhagen, Denmark; 30 Department of Dermatology, Zealand University Hospital, Roskilde, Denmark; 31 Department of Health Science and Technology, Faculty of Medicine, Aalborg University, Aalborg, Denmark; 32 Institute of Biological Psychiatry, Mental Health Centre, Sct. Hans, Copenhagen University Hospital, Roskilde, Denmark

**Keywords:** Genome-wide association studies, Neuroscience

## Abstract

Migraine is a complex neurovascular disease with a range of severity and symptoms, yet mostly studied as one phenotype in genome-wide association studies (GWAS). Here we combine large GWAS datasets from six European populations to study the main migraine subtypes, migraine with aura (MA) and migraine without aura (MO). We identified four new MA-associated variants (in *PRRT2*, *PALMD*, *ABO* and *LRRK2*) and classified 13 MO-associated variants. Rare variants with large effects highlight three genes. A rare frameshift variant in brain-expressed *PRRT2* confers large risk of MA and epilepsy, but not MO. A burden test of rare loss-of-function variants in *SCN11A*, encoding a neuron-expressed sodium channel with a key role in pain sensation, shows strong protection against migraine. Finally, a rare variant with *cis*-regulatory effects on *KCNK5* confers large protection against migraine and brain aneurysms. Our findings offer new insights with therapeutic potential into the complex biology of migraine and its subtypes.

## Abstract (web summary)

Genome-wide association analyses of migraine and its subtypes identify new susceptibility loci, including rare variants with large effects implicating *PRRT2*, *SCN11A* and *KCNK5*.

## Main

Migraine is a complex neurovascular disease characterized by recurrent, disabling headache attacks that are difficult to treat. It is among the most common pain disorders worldwide, with prevalence of up to 20% in adult populations and affecting three times more females than males^1^. Two main subtypes are clinically distinguished, migraine with aura (MA) and migraine without aura (MO)^2^. MO is characterized by severe headache attacks accompanied by nausea and hypersensitivity to light and sound, whereas MA is characterized by gradually spreading, fully reversible focal neurological symptoms, collectively called aura, that are usually followed by headache^1^. An estimated 30% of migraineurs have MA, and the most frequently experienced aura involves visual disturbances (for example, flashes of bright light and blurred vision)^3^. During MA attacks, characteristic regional brain blood flow changes indicate that MA is caused by cortical spreading depression, a transient wave of neuronal depolarization of the cortex^4,5^. Such findings are not observed in MO^6,7^, suggesting divergent pathogenesis of these migraine subtypes. A rare and clinically distinct subtype of MA is familial hemiplegic migraine (FHM)^2^. Three genes have been linked to FHM—one encoding a membrane protein involved in maintaining gradients of sodium and potassium ions across plasma membranes (*ATP1A2*), and two genes encoding sodium and calcium channels expressed in brain (*SCN1A* and *CACNA1A*, respectively)^8^.

More is known about the genetics and biology of migraine than any other pain disorder, leading to recent treatment advances such as those targeting the calcitonin gene-related peptide (CGRP) activation of the trigeminovascular system^9,10^. The largest genome-wide association studies (GWAS) meta-analysis of migraine to date identified 123 migraine risk loci, among them a locus including genes encoding CGRP (*CALCA* and *CALCB*)^11^. However, the pathophysiology of migraine is not fully understood, and a substantial subset of patients has treatment-resistant migraine^12^. In the study reporting 123 common (minor allele frequency (MAF) > 2%) migraine variants, subtype analysis showed that 5 associate specifically with migraine subtypes—3 with MA (in or near *CACNA1A*, *HMOX2* and *MPPED2*) and 2 with MO (near *SPINK2* and *FECH*)^11,13^. These findings suggest that the genetics of MA and MO should be studied separately and with more emphasis on detecting rare variants.

To identify both distinct and common biological underpinnings of these migraine subtypes, we performed GWAS meta-analyses of clinically defined MA, MO and overall migraine, using six datasets and analyzing variants down to 0.001% in frequency. We used samples from about 1.3 million individuals, of which 12,000 have MO, 17,000 have MA and 80,000 have migraine. Because migraine and especially its subtypes are considerably underdiagnosed^14^, and to obtain measures of specific symptoms and severity, we also assessed self-reported proxy phenotypes representing severe and recurrent migraine headaches (52,000 cases) as well as migraine’s most distinctive subtype, headaches preceded by visual aura (30,000 cases). Here we report 4 new MA-associated variants and show that 13 known migraine variants associate with MO over MA. In all, we observed associations with 44 lead variants, 12 of which are new for migraine, and we found functional evidence implicating 22 genes—3 in MA, 3 in MO and the remainder in overall migraine. Among the findings are rare variants with large effects providing new insights into biological underpinnings of distinct characteristics of migraine, with and without aura.

## Results

**Fig. 1:**
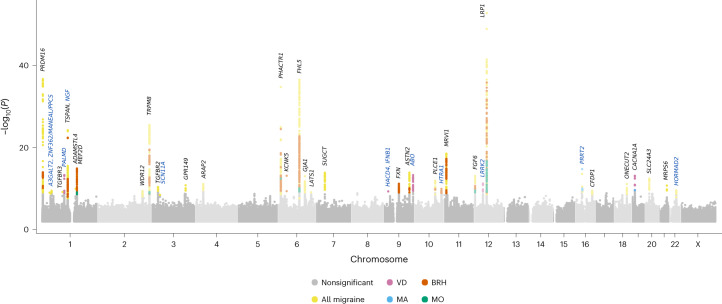
Manhattan plot of GWAS meta-analysis results for all studied phenotypes. The graph shows data for migraine (*n*_case/control_ = 74,495/1,259,808), MA (*n*_case/control_ = 16,603/1,336,517), MO (*n*_case/control_ = 11,718/1,330,747), VD (*n*_case/control_ = 30,297/86,134) and BRH (*n*_case/control_ = 51,803/123,732). See Supplementary Table 1 for *n*_case/control_ for each cohort. On the *x* axis, variants are plotted along the 22 autosomes and the X chromosome. On the *y* axis is the statistical significance of their association with the respective phenotypes from meta-analyses using a fixed-effects inverse-variance method based on effect estimates and s.e. under the additive model, in which each dataset was assumed to have a common OR but allowed to have different population frequencies for alleles and genotypes. Gray dots are not significant variants. Variant associations that reach the *P* threshold weighted by variant annotation^21^ are represented by color-coded dots. Adjacent chromosomes are presented in different shades of gray. Known migraine loci are represented by gene names in black text, and new loci are represented by gene names in blue text.

We conducted GWAS meta-analyses of clinically defined migraine, MA and MO, using datasets from Iceland (deCODE Genetics), Denmark (Copenhagen Hospital Biobank (CHB)^15^ and Danish Blood Donor Study (DBDS)^16^), the United Kingdom (UK; UK Biobank^17^), the United States (US; Intermountain Health^18^), Norway (the Hordaland Health Study (HUSK)^19^) and Finland (FinnGen^20^). We also performed GWAS meta-analyses of two self-reported proxy phenotypes available in three datasets (Iceland, UK and Denmark)—an MA proxy represented by experiencing visual disturbances (VD) preceding headaches, and a severe migraine proxy represented by bad and recurrent headaches (BRH). In total, we analyzed data on 1.3 million individuals, including 16,603 with MA, 11,718 with MO, 79,495 with any migraine, 30,297 with VD and 51,803 with BRH (Methods; Supplementary Table 1). We analyzed up to 85 million variants, and using a significance threshold weighted by variant impact^21^, we found associations with 44 lead variants at 39 loci (Fig. 1, Tables 1 and 2 and Supplementary Tables 2–7). Two variants associate with MA (one new), five with the MA-proxy VD (four new) and six with MO. The remaining variants associate with overall migraine or BRH. In all, we report 12 new migraine variants (regional plots shown in Supplementary Figs. 1 and 2).

**Table 1:** Lead variants associated with migraine subtypes and headache-related visual disturbances (MA proxy)

Phenotypes	Locus	Position hg38	Variants	OA	EA	EAF (%)	Nearest genes	Variant annotation	OR (95% CI)	*P*	*P*_bonf_	*P*_het_	SNP previously reported at locus (*r*^2^ if correlated SNP)
MA	16p11.2	29813694	rs587778771	GC	GCC	0.05	*PRRT2*	Frameshift	5.446 (3.626, 8.148)	5.6 × 10^−16^	6.7 × 10^−10^	–	–
MA	19p13.13	13228314	rs10405121	G	A	27.3	*CACNA1A*	Intron	0.927 (0.905, 0.949)	2.5 × 10^−10^	0.03	0.24	rs10405121^a^
VD	1p21.2	99579683	rs11166276	T	C	49.0	*PALMD*	TF-binding site	0.926 (0.907, 0.945)	5.1 × 10^−14^	2.2 × 10^−6^	0.87	–
VD	9p21.3	21047562	rs77778288	A	C	12.9	*HACD4/IFNB1*	Regulatory region	1.097 (1.065, 1.129)	4.9 × 10^−10^	0.02	0.77	–
VD	9q34.2	133257521	rs8176719	T	TC	32.9	*ABO*	Frameshift	1.081 (1.059, 1.104)	3.0 × 10^−13^	1.2 × 10^−7^	0.07	–
VD	12q12	40221267	rs10748014	C	T	46.8	*LRRK2*	Upstream	1.073 (1.052, 1.094)	5.6 × 10^−12^	0.00012	0.11	–
VD	19p13.13	13234712	rs11085837	G	A	45.6	*CACNA1A*	Intron	0.926 (0.907, 0.945)	8.8 × 10^−14^	3.7 × 10^−6^	0.18	rs10405121 (0.96)^a^
**MO**	**1q22**	**156460957**	rs750439^**c**^	**T**	**C**	**33.9**	***MEF2D***	**Downstream**	**1.092 (1.062, 1.123)**	**8.7** × **10**^**−10**^	**0.017**	**0.06**	rs2274319 (0.64)^a^, rs1925950 (0.86)^b^
MO	2q37.1	233937757	rs12470426^c^	G	A	9.1	*TRPM8*	Intron	0.853 (0.812, 0.897)	6.0 × 10^−10^	7.6 × 10^−2^	0.56	rs10166942 (0.39)^a^, rs10166942 (0.96)^b^
**MO**	**6p24.1**	**12903725**	rs9349379^**c**^	**A**	**G**	**41.7**	***PHACTR1***	**3**′ **UTR**	**0.904 (0.879, 0.929)**	**3.8** **×** **10**^**−13**^	**8.0** **×** **10**^**−6**^	**0.35**	rs9349379^**a,b**^
MO	6q16.1	96610677	rs2273621^c^	A	G	32.3	*FHL5*	Missense	1.096 (1.065, 1.128)	3.0 × 10^−10^	1.9 × 10^−3^	0.61	rs11153082 (1.0)^a^, rs67338227 (0.54)^b^
**MO**	**12p13.32**	**4418156**	rs2160875^**c**^	**T**	**C**	**49.1**	***FGF6***	**Regulatory region**	**1.088 (1.059, 1.118)**	**8.2** **×** **10**^**−10**^	**3.4** **×** **10**^**−2**^	**0.85**	rs2160875^a^, rs1024905 (0.83)^b^
**MO**	**12q13.3**	**57132863**	rs4759276^**c**^	**G**	**A**	**39.9**	***LRP1***	**Intron**	**0.889 (0.865, 0.914)**	**9.8** **×** **10**^**−17**^	**1.3** **×** **10**^**−8**^	**0.32**	rs11172113**(0.84)**^**a,b**^

Supplementary Table 1 shows *n*_case/control_ per cohort and Supplementary Table 7 shows associations of these variants with all migraine. Discovery phenotype is in the first column: MA, headache-related VD proxy for MA, MO. Effect allele frequency (EAF) is the average frequency of EA in the cohorts studied (Supplementary Table 1; Methods). OR and *P* value for inverse-variance weighted meta-analysis of association results for all cohorts. *P*_bonf_ is the *P* value after a variant class-specific Bonferroni adjustment^21^. *P*_het_ is the heterogeneity *P* value from a likelihood ratio test. Results per cohort and for all phenotypes are in Supplementary Tables 2–7. Associations of these and correlated variants (*r*^2^ > 0.8) with various traits listed in the GWAS Catalog (https://www.ebi.ac.uk/gwas/) are in Supplementary Table 12. Bold are variants that associate primarily with MO, over MA or VD (Fig. 3).

CI, confidence interval; EA, effect allele; OA, other allele.

^a^SNPs previously reported in ref. ^11^.

^b^SNPs previously reported in ref. ^69^.

^c^MO-associated variants that also correlate with migraine variants in Table 2; rs750439 (*r*^*2*^ = 0.64) with rs1925950, rs12470426 (*r*^*2*^ = 0.39) with rs1003540, rs2160875 (*r*^*2*^ = 1.0) with rs7957385, rs4759276 (*r*^*2*^ = 0.84) with rs11172113 and rs9349379 and rs2273621 also associate with migraine.

**Table 2:** Variants identified in association with all migraine (M) or migraine proxy (BRH)

Phenotypes	Locus	Position hg38	Variants	OA	EA	EAF (%)	Nearest genes	Variant annotation	OR (95% CI)	*P*	*P*_bonf_	*P*_het_	SNP previously reported at locus (*r*^2^ if correlated SNP)
M	1p36.32	3155918	rs10797381	T	A	22.7	*PRDM16*	Intron	1.094 (1.079, 1.109)	2.1 × 10^−37^	8.7 × 10^−30^	0.08	rs10218452 (1.0)^a^, rs2651899 (0.37)^b^
M	1p36.1	33302206	rs933718575	A	G	0.01	*A3GALT2*	Downstream	11.032 (5.11, 23.8)	9.7 × 10^−10^	0.020		–
M	**1p34.3**	**37790755**	rs71642605	**T**	**C**	**25.3**	***MANEAL***	**Upstream**	**1.042 (1.028, 1.056)**	**1.1** **×** **10**^**−9**^	**0.023**	**0.89**	–
M	1p34.2	42465863	rs11799356	G	A	34.2	*PPCS*	Downstream	1.039 (1.026, 1.052)	6.0 × 10^−10^	0.013	0.67	–
M	1p22.1	91731541	rs12070846	T	C	23.0	*TGFBR3*	Intron	1.044 (1.030, 1.058)	6.8 × 10^−10^	0.029	0.16	rs11165300 (0.88)^a^
M	1p13.2	115135325	rs12134493^c^	C	A	12.0	*TSPAN2*	TF-binding site	1.112 (1.092, 1,132)	1.7 × 10^−30^	7.2 × 10^−23^	0.03	rs2078371 (1.0)^a^, rs12134493^b^
M	1p13.2	115286692	rs6330^c^	G	A	46.7	*NGF*	Missense	1.035 (1.023–1.048)	2.1 × 10^−8^	0.041	0.06	–
**M**	**1q21.1**	**150538184**	rs6693567	**T**	**C**	**26.0**	***ADAMSTL4***	**Regulatory region**	**1.044 (1.031, 1.058)**	**1.1** **×** **10**^**−10**^	**0.0046**	**0.64**	**rs6693567**^**a**^**,**^**b**^
M	**1q22**	**156480948**	rs1925950	**A**	**G**	**36.0**	***MEF2D***	**Synonymous**	**1.047 (1.034, 1.059)**	**6.3** **×** **10**^**−14**^	**1.3** **×** **10**^**−6**^	**0.08**	rs2274319**(1.0)**^**a**^, rs3790455**(1.0)**^**b**^
M	2q33.2	202901033	rs35212307	T	C	12.6	*WDR12*	Missense	0.949 (0.933, 0.966)	6.7 × 10^−9^	0.013	0.54	rs149163995 (0.99)^b^
M	2q37.1	233917239	rs1003540	A	G	19.4	*TRPM8*	Upstream	0.923 (0.910, 0.937)	3.3 × 10^−26^	6.9 × 10^−19^	0.50	rs10166942 (1.0)^a^
M	3p24.1	30424073	rs4955309	C	A	31.9	*TGFBR2*	Intergenic	1.042 (1.030, 1.055)	4.0 × 10^−11^	0.005	0.10	rs7371912 (0.91)^a^, rs7640543 (0.97)^b^
M	3p22.2	38894643	rs33985936	C	T	25.0	*SCN11A*	Missense	1.041 (1.027, 1.054)	3.4 × 10^−9^	0.0065	0.32	–
M	3q25.2	154572157	rs13078967	A	C	3.5	*GPR149*	Regulatory region	0.892 (0.862, 0.922)	1.6 × 10^−11^	0.00066	0.27	rs13078967^a,b^
**M**	**4p15.1**	**35563301**	rs74992952	**G**	**A**	**17.9**	***ARAP2***	**Intergenic**	**0.949 (0.935, 0.963)**	**8.8** **×** **10**^**−12**^	**0.00037**	**0.45**	**rs73805934 (0.92)**^**a**^
**M**	**6p24.1**	**12903725**	rs9349379	**A**	**G**	**41.9**	***PHACTR1***	**3′ UTR**	**0.928 (0.917, 0.939)**	**1.9** **×** **10**^**−35**^	**3.9** **×** **10**^**−28**^	**0.43**	**rs9349379**^**a,b**^
BRH	6p21.2	39280316	rs72854118	A	G	0.67	*KCNK5*	TF-binding site	0.697 (0.634, 0.766)	7.6 × 10^−14^	3.2 × 10^−6^	0.10	–
M	6q16.1	96610677	rs2273621	A	G	32.3	*FHL5*	Missense	1.082 (1.069, 1.096)	1.1 × 10^−36^	2.1 × 10^−30^	0.14	rs11153082 (0.99)^a^, rs11759769 (0.55)^b^
M	6q22.31	121487928	rs7743275	G	A	19.9	*GJA1*	Regulatory region	1.060 (1.044, 1.077)	9.7 × 10^−14^	1.2 × 10^−5^	0.17	rs28455731 (0.73)^a,b^
M	6q25.1	149721026	rs1359155039	TAAAAAAAA	TAAAAAAAAA	32.8	*LATS1*	Upstream	0.958 (0.945, 0.971)	8.1 × 10^−10^	0.017	0.38	rs9383843 (0.87)^a^
M	**7p14.1**	**40367277**	rs186166891	**A**	**T**	**10.4**	***SUGCT***	**Intron**	**1.084 (1.062, 1.106)**	**1.1** **×** **10**^**−14**^	**1.4** **×** **10**^**−6**^	**0.40**	**rs10234636 (0.91)**^**a**^, rs4379368 (**0.91)**^**b**^
BRH	9q21.11	69099647	rs34965002	G	A	43.3	*FXN/TJP2*	Regulatory region	1.056 (1.039, 1.072)	6.6 × 10^−12^	0.000277	0.19	rs7034179 (0.87)^a^
**M**	**9q33.1**	**116479356**	rs12684144^**d**^	**T**	**C**	**22.3**	***ASTN2***	**Intron**	**1.055 (1.041, 1.070)**	**1.3** **×** **10**^**−14**^	**5.4** **×** **10**^**−7**^	**0.01**	rs3891689**(0.91)**^**a**^, rs6478241 (**0.57)**^**b**^
M	**10q23.33**	**94279840**	rs2274224	**G**	**C**	**41.5**	***PLCE1***	**Missense**	**0.959 (0.948, 0.970)**	**2.7** **×** **10**^**−12**^	**5.1** **×** **10**^**−6**^	**0.04**	**rs2274224**^**a**^**,** **rs11187838 (1.0)**^**b**^
BRH	10q26.13	122470997	rs12252027	G	T	11.4	*HTRA1*	Intron	0.926 (0.904, 0.948)	1.2 × 10^−10^	0.0149	0.73	–
**M**	**11p15.4**	**10652192**	rs4909945	**C**	**T**	**33.0**	***MRVI1***	**Missense**	**0.945 (0.934, 0.957)**	**3.1** **×** **10**^**−19**^	**5.9** **×** **10**^**−13**^	**0.61**	rs4910165**(1.0)**^**a,b**^
M	**12p13.32**	**4416380**	rs7957385	**G**	**A**	**48.6**	***FGF6***	**Intergenic**	**1.045 (1.033, 1.057)**	**8.2** **×** **10**^**−14**^	**1.0** **×** **10**^**−5**^	**0.03**	rs2160875**(1.0)**^**a**^**,** **rs140668749 (1.0)**^**b**^
**M**	**12q13.3**	**57133500**	rs11172113	**T**	**C**	**42.3**	***LRP1***	**Intron**	**0.912 (0.901, 0.923)**	**1.8** **×** **10**^**−53**^	**7.4** **×** **10**^**−46**^	**0.42**	**rs11172113**^**a,b**^
M	16p11.2	29813694	rs587778771	GC	GCC	0.05	*PRRT2*	Frameshift	3.038 (2.320, 3.977)	6.6 × 10^−16^	1.0 × 10^−9^	0.83	–
M	16q23.1	75289942	rs17685540	C	T	41.0	*CFDP1*	Downstream	1.037 (1.025, 1.049)	1.4 × 10^−9^	0.029	0.31	rs8046696 (0.98)^a^, rs77505915 (0.91)^b^
M	18q21.31	57494932	rs7233335	C	G	20.4	*ONECUT2*	Downstream	0.954 (0.941, 0.968)	1.8 × 10^−10^	0.0038	0.01	rs8087942 (0.45)^a^
M	**20p11.23**	**19494370**	rs3827986	**G**	**A**	**24.5**	***SLC24A3***	**Intron**	**1.050 (1.036, 1.064)**	**4.6** **×** **10**^**−13**^	**1.9** **×** **10**^**−5**^	**0.52**	rs4814864**(1.0)**^**a**^
M	**21q22.11**	**34221526**	rs28451064	**G**	**A**	**13.6**	***MRPS6***	**Regulatory region**	**0.943 (0.927, 0.959)**	**1.8** **×** **10**^**−11**^	**0.00075**	**0.2**	**rs28451064**^**a**^
M	22q12.2	30076759	rs5753008	T	C	35.6	*HORMAD2*	Upstream	1.039 (1.027, 1.051)	4.0 × 10^−10^	0.0084	0.5	–

Effect allele frequency (EAF) is the average frequency of EA in the cohorts studied. OR and *P* value for inverse-variance-weighted meta-analysis of association results for all cohorts (Supplementary Table 1; Methods). *P*_bonf_ is the *P* value after a variant class-specific Bonferroni adjustment ^21^. *P*_het_ is the heterogeneity *P* value from a likelihood ratio test. Bold are variants that associate primarily with MO, not MA or VD, or with larger effects on MO than on MA or VD (Fig. 3).

^a^SNPs previously reported in ref. ^11^.

^b^SNPs previously reported in ref. ^69^.

^c^Results presented are after adjusting for the respective effects of these uncorrelated (*r*^2^ = 0.02) variants at this locus. Results per cohort for all studied phenotypes are in Supplementary Tables 2–7.

^d^rs12684144-C confers protection against VD and risk against MO.

Using cross-trait linkage disequilibrium (LD) score regression^22^, we calculated genetic correlations in nonoverlapping samples (Methods) showing that VD correlates genetically with clinically defined MA (*r*_*g*_ = 0.65, *P* = 4.0 × 10^−23^) but not MO (*r*_*g*_ = −0.09, *P* = 0.21), and BRH correlate strongly with clinically defined migraine (*r*_*g*_ = 0.85, *P* = 7.4 × 10^−91^; Supplementary Table 8 and Supplementary Fig. 3). Further supporting VD as an MA proxy, the GWAS meta-analysis of VD reveals an association with a variant (rs11085837-A) in high LD (*r*^*2*^ = 0.96) with the reported MA variant in *CACNA1A*, rs10405121-A^11^ (Fig. 1 and Table 1). Its VD effect (odds ratio (OR) = 0.926, *P* = 8.8 × 10^−14^) is consistent with its MA effect (OR = 0.930, *P* = 1.8 × 10^−9^), and no association is detected with MO (OR = 0.983, *P* = 0.22). In Supplementary Table 9, we list associations with all migraine phenotypes of the current study with the recently published 123 migraine variants^11^, finding support (*P* < 0.05) in our data for all but 9 variants (Supplementary Note 1).

### A rare loss-of-function *PRRT2* variant associates with MA

The top MA association is with a rare insertion in *PRRT2* leading to frameshift (rs587778771-GCC, p.Arg217ProfsTer8; OR = 5.446, *P* = 5.6 × 10^−16^). This variant also associates with VD (OR = 3.634, *P* = 0.0037) but not MO (*P* = 0.97; Table 3). It is detected in only three cohorts, with a founder effect observed in Iceland (frequency = 0.117%), compared to UK and US (frequency = 0.013% and 0.0051%, respectively). It is detected at even lower frequencies in samples from Denmark, with no carriers detected in Norway or Finland. This variant has been reported in case studies of rare neurological disorders, including benign infantile seizures and paroxysmal kinesigenic dyskinesia (PKD)^23^. In a few carriers, FHM has also been detected^8^. Among six Danish heterozygous carriers identified, five are in the same family, of which three have FHM.

**Table 3:** GWAS meta-analysis results for *PRRT2* frameshift variant (p.Arg217ProfsTer8)

Phenotypes	Iceland (MAF = 0.117%)		UK (MAF = 0.013%)		US (MAF = 0.0051%)		Combined		
	OR (95% CI)	*P*	OR (95% CI)	*P*	OR (95% CI)	*P*	OR (95% CI)	*P*	*P*_het_
Epilepsy	7.482 (5.398, 10.370)	1.3 × 10^−33^	4.284 (1.548, 11.859)	0.0051	5.455 (0.407, 73.054)	0.20	7.077 (5.197, 9.635)	1.9 × 10^−35^	0.58
MA	5.534 (3.631, 8.434)	1.7 × 10^−15^	3.019 (0.283, 32.163)	0.36	5.869 (0.712, 48.348)	0.10	5.446 (3.626, 8.148)	5.6 × 10^−16^	0.88
Migraine	3.129 (2.333, 4.196)	2.6 × 10^−14^	2.482 (1.202, 5.125)	0.014	3.553 (0.489, 25.791)	0.21	3.038 (2.320, 3.977)	6.6 × 10^−16^	0.83
BRH	5.276 (2.104, 13.227)	3.9 × 10^−4^	1.981 (0.857, 4.581)	0.11	–	–	3.091 (1.664, 5.742)	3.6 × 10^−4^	0.12
VD	8.344 (1.952, 35.662)	4.2 × 10^−3^	2.274 (0.764, 6.771)	0.14	–	–	3.634 (1.519, 8.696)	3.7 × 10^−3^	0.16
MO	1.025 (0.283, 3.712)	0.97	0.017 (0.000, 4357619.32)	0.68	0.017 (0.000, 15176.02)	0.56	0.972 (0.271, 3.489)	0.97	0.78

The table shows OR with 95% CI and two-sided *P* values from GWAS results derived from a logistic regression of selected phenotypes in the three cohorts where p.Arg217ProfsTer8 was detected at sufficient frequency. Combined OR and two-sided *P* are results from inverse-variance-weighted meta-analyses of GWAS results. *P* values after a variant class-specific Bonferroni adjustment^21^. *P*_het_ is the heterogeneity *P* value from a likelihood ratio test. See Supplementary Table 1 for cohort descriptions and Supplementary Table 10b for other neurological associations with both rare *PRRT2* frameshift variants (p.Arg217ProfsTer8 and p.Arg217GlufsTer12).

The p.Arg217ProfsTer8 insertion is located in an unstable DNA site^24,25^ where we find another rarer (0.024%) deletion (p.Arg217GlufsTer12) that also leads to premature PRRT2 truncation^25^. This variant also shows a founder effect in Iceland, being tenfold more frequent than in the UK (frequency of 0.0025%), and not detected in other cohorts. It was previously reported in a single case study of a homozygous carrier with severe PKD that responded to carbamazepine, an epilepsy drug that reduces the generation of rapid action potentials in the brain^26^ and is also used to treat migraine. We found p.Arg217GlufsTer12 in 38 heterozygous carriers in Iceland, mainly in two families where it segregates with migraine and epilepsy. Of 38 carriers, 11 (29%) are diagnosed with migraine (without subtype), six (16%) with epilepsy and one with MA and epilepsy.

For these rare variants, we looked for associations with other phenotypes. Apart from the MA and migraine associations, p.Arg217ProfsTer8 associates only with epilepsy (OR = 7.077, *P* = 1.9 × 10^−35^; Table 3 and Supplementary Table 10). We find epilepsy moderately genetically correlated with migraine (*r*_*g*_ = 0.28, *P* = 9.4 × 10^−6^) and VD (*r*_*g*_ = 0.28, *P* = 2.8 × 10^−4^), but not with MO (*r*_*g*_ = 0.05, *P* = 0.90). We tested 30 epilepsy variants^27^ in our data and found that only two also impact migraine (at *P* < 3.3 × 10^−4^ = 0.05/30 variants × 5 phenotypes). The common (23.3%) intron variant rs59237858-T in *SCN1A* that confers protection against epilepsy^27^ confers risk of migraine (OR = 1.031, *P* = 8.6 × 10^−6^) in our data, and rs62151809-T (44.7%) near *TMEM182* confers risk of epilepsy^27^ and of VD in our data (OR = 1.047, *P* = 8.5 × 10^−6^). None of the 30 epilepsy variants associate with MO or BRH (Supplementary Table 11). Conversely, of the 44 variants reported here, only p.Arg217ProfsTer8 associates with epilepsy.

### GWAS meta-analysis of MA-proxy phenotype yields new MA-associated loci

Besides the known MA-associated variant in *CACNA1A*, we found four other variants associating with the MA-proxy VD, all new to migraine (Table 1). The first, rs11166276-C, is in a TF-binding site near *PALMD* (OR = 0.926, *P* = 5.1 × 10^−14^). It is in complete LD with rs7543130 that also associates protectively with aortic valve stenosis^28^. Secondly, in *ABO*, the frameshift variant rs8176719-TC associates with VD (OR = 1.081, *P* = 3.0 × 10^−13^). This variant contributes to determining the non-O blood groups^29^, and variants in high LD associates with various coagulation factors and risk of venous thromboembolism (Supplementary Table 12). This variant associates with MA (OR = 1.030, *P* = 0.015) and overall migraine (OR = 1.020, *P* = 1.5 × 10^−3^; Supplementary Table 7). Thirdly, a variant upstream of *LRRK2*, rs10748014-T, associates with VD (OR = 1.073, *P* = 5.6 × 10^−12^). *LRRK2* encodes leucine-rich repeat kinase 2, a gene harboring common risk variants for inherited Parkinson’s disease (PD)^30^, none of which are in LD with rs10748014 (Supplementary Table 12). This variant also associates with MA (OR = 1.065, *P* = 8.4 × 10^−8^) and weakly with overall migraine (OR = 1.012, *P* = 0.048), and we detected no association with MO or PD. Finally, in a regulatory region near *HACD4/IFNB1* is an association with rs77778288-C (frequency = 12.9%, OR = 1.097, *P* = 4.9 × 10^−10^). *IFNB1* encodes interferon β 1, which is used to treat multiple sclerosis and can induce headaches^31^.

**Fig. 2:**
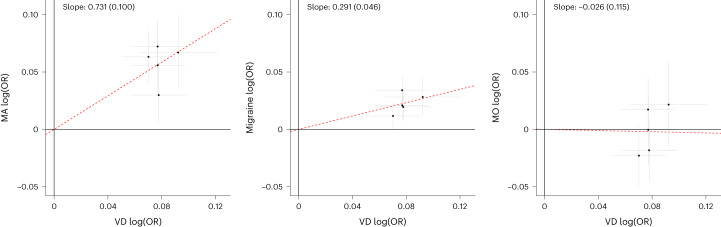
Effects of SNPs associated with self-reported headache-related VD in clinically defined MA, overall migraine and MO. The *x* axis (VD, *n*_case/control_ = 30,297/86,134) and the *y* axis (MA, *n*_case/control_ = 16,603/1,336,517; migraine, *n*_case/control_ = 74,495/1,259,808 and MO, *n*_case/control_ = 11,718/1,330,747) show the logarithmic estimated odds ratios, log(OR), for the associations with the respective phenotypes from meta-analyses using a fixed-effects inverse-variance method based on effect estimates and s.e. under the additive model, in which each dataset was assumed to have a common OR but allowed to have different population frequencies for alleles and genotypes. All effects are shown for the VD risk allele, and black crosses indicate 95% CIs. The dashed red lines represent slope (s.d.) based on a simple linear regression through the origin using 1/s.e. as weights. Effect estimates are 73%, 29% and 0% of VD effect estimates for MA, migraine and MO, respectively.

We compared the effects of these VD variants on MA and all migraine in effect–effect plots (Fig. 2). Based on the slope derived from a weighted regression through the origin, overall MA and migraine effect estimates are 73% and 29%, respectively, of VD effect estimates, and no associations were detected for MO, which is in line with our estimates of genetic correlation between these traits.

### Migraine subtype classification of lead variants

**Fig. 3:**
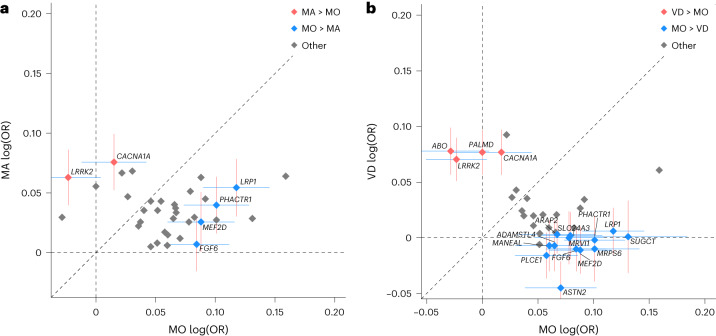
Subtype classification of lead variants. Effect plots for all lead variants except the MA variant in *PRRT2*. Effects are from meta-analyses using a fixed-effects inverse-variance method based on effect estimates and s.e. under the additive model, in which each dataset was assumed to have a common OR but allowed to have different population frequencies for alleles and genotypes. Data are presented as additive effect estimates (center) with 95% CI (crosses) for the annotated variants. **a**, Axes show logarithm of odds ratios (log(OR)) for MO (*x* axis; *n*_case/control_ = 11,718/1,330,747) and MA (*y* axis; *n*_case/control_ = 16,603/1,336,517). **b**, Axes show MO (*x* axis; *n*_case/control_ = 11,718/1,330,747) and VD (*y* axis; *n*_case/control_ = 30,297/86,134). log(OR) is calculated for the effect allele. The effects of variants that have been colored and annotated with gene names differ between the migraine subtypes at a significance threshold of 0.0012 = 0.05/43. The 95% CIs for the log(ORs) are shown for annotated variants. Effects are adjusted with sample overlap (*r*_*ij*_) estimated from counts of cases, controls and the counts of overlaps in these groups between phenotypes^70^ from all cohorts except FinnGen (for which we only have summary statistics). The parameter representing sample overlap between MO and MA is *r*_*ij*_ = 0.023 and MO and VD is *r*_*ij*_ = 0.012. Dashed lines show the coordinate axes, the diagonal and a line through the origin with slope = 1 (Methods; see Supplementary Tables 13 and 14 and Supplementary Fig. 4 for VD versus MA plot).

We used a similar approach discussed in ref. ^11^ to study the effects of 43 lead variants on the migraine subtypes adjusting for sample overlap (*PRRT2* excluded as it has larger effects than other variants and is shown to be an MA-associated variant; Methods). We find that the new variants in *ABO*, *LRRK2* and *PALMD*, and the previously reported^11^ MA-associated variant in *CACNA1A* are classified as MA-associated variants, and 13 variants are classified as MO-associated variants (bold in Tables 1 and 2; Fig. 3 and Supplementary Fig. 4). All MO-associated variants are in known migraine loci except the new MO-associated variant rs71642605-C in *MANEAL*. We find that one of the MO-associated variants, rs12684144-C in *ASTN2*, confers protection against VD (OR = 0.956, *P* = 0.00017) but risk of MO (OR = 1.073, *P* = 1.5 × 10^−5^). In line with only 30% of migraineurs experiencing aura^3^, its association with overall migraine confers risk (OR = 1.055, *P* = 1.3 × 10^−14^).

**Fig. 4:**
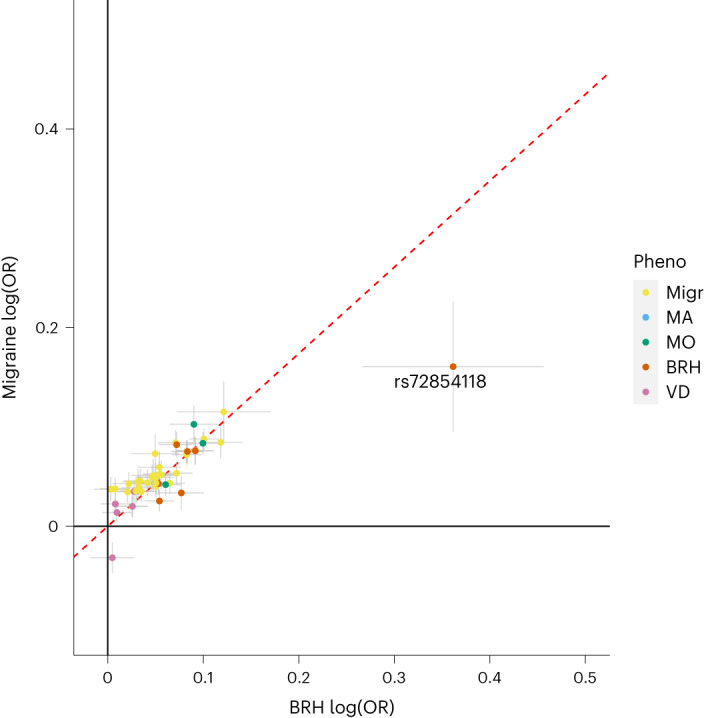
Rare variant rs72854118 in regulatory region targeting *KCNK5* associates with BRH. Effect–effect plot of clinically defined migraine (*n*_case/control_ = 74,495/1,259,808) vs. self-reported BRH (*n*_case/control_ = 51,803/123,732) effects for 42 lead variants identified in this study (excluding high-impact variants in *PRRT2* and *A3GALT2*; see Supplementary Table 7 for their associations with the respective phenotypes). Effects are from meta-analyses using a fixed-effects inverse-variance method based on effect estimates and s.e. under the additive model, in which each dataset was assumed to have a common OR but allowed to have different population frequencies for alleles and genotypes. The *x* axis and the *y* axis show the logarithmic estimated ORs for the associations with the respective phenotypes. Error bars represent 95% CI. The dashed red lines represent slope (s.d.) based on a simple linear regression through the origin using 1/s.e. as weights. Cohort descriptions are in Supplementary Table 1. Variants are colored according to their primary associations in this study. The red dot outlier depicts the variant rs72854118-G near *KCNK5*, its effects on BRH exceeding its effects on all migraine. Pheno, phenotype; Migr, migraine.

### Protein-altering variants in *NGF* and *SCN11A*

Among new variants associated with overall migraine is the common missense variant rs6330-A (p.Ala35Val) in *NGF* (OR = 1.035, *P* = 2.1 × 10^−8^). *NGF* encodes nerve growth factor that is involved in regulating growth and differentiation of sympathetic and certain sensory neurons (https://www.ncbi.nlm.nih.gov/gene). *NGF* is at 1p13.2 and nearby is *TSPAN2*, harboring a previously reported^11^ migraine-associated variant (rs2078371) that is, however, uncorrelated (*r*^2^ = 0.02) with rs6330. Conditional analysis shows that the effects of rs6330-A on migraine are significant when adjusting for rs2078371 (Table 2).

In *SCN11A*, another common (25%) missense variant, rs33985936-T (p.Val909Ile), associates with overall migraine (OR = 1.041, *P* = 3.4 × 10^−9^). *SCN11A* encodes Na_v_1.9, which is highly expressed in nociceptive neurons of dorsal root and trigeminal ganglia^32,33^. Rare loss-of-function (LOF) variants in *SCN11A* can lead to both extremely painful and completely pain-insensitive disorders^32,33^. We looked for LOF variants in *SCN11A* and found them at very low frequency in all datasets studied, with the highest in the UK at a combined frequency of 0.13%, which is two orders of magnitude higher than in other cohorts. We used a genome-wide burden test combining the effects of these rare variants on migraine in the UK, and at a threshold of *P* = 2.5 × 10^−6^ (*P* = 0.05/20,000 genes^34^ tested), they associate with strong protection against overall migraine (OR = 0.650, *P* = 3.9 × 10^−7^) and other severe headaches and are not driven by a single variant (Table 4 and Supplementary Note 2).

**Table 4:** Results of *SCN11A* LOF variant burden tests in the respective cohorts for association with migraine

Cohorts	Unique LOF variants combined	Combined frequency (%)	OR	*P* value	*n*_cases_	*n*_controls_
UK Biobank	127	0.129	0.650	3.90 × 10^−7^	22,082	408,965
US	26	0.0254	0.751	0.63	7,427	50,785
Denmark	26	0.0183	0.629	0.43	14,371	266,473
Iceland	5	8.79 × 10^−3^	0.882	0.83	24,604	319,066
–	Combined	0.0454	0.660	2.90 × 10^−7^	68,484	1,045,289

The table shows a number of unique LOF variants tested in each cohort. We classified as high-impact variants those predicted as start-lost, stop-gain, stop-lost, splice donor, splice acceptor or frameshift. We used logistic regression under an additive model to test for association between LOF gene burdens and phenotypes using likelihood ratio test to compute two-sided *P* values (Methods; see Supplementary Note 2 for other headache associations in UK Biobank data).

### A rare variant targeting *KCNK5* with protective effects

In the GWAS meta-analysis of BRH, there is an association with a large protective effect (OR = 0.697, *P* = 7.6 × 10^−14^) with the rare (0.67%) intergenic variant rs72854118-G located in a regulatory region between two potassium channel genes, *KCNK5* and *KCNK17*. The variant also protects against clinically defined migraine (OR = 0.836, *P* = 9.7 × 10^−7^), but does not associate with migraine subtypes, MA, MO or VD (*P* > 0.05). Two additional variants in high LD are at this locus, rs72854120 and rs72851880 (Supplementary Fig. 2). A common (28.1%) intronic variant in *KCNK5* was previously reported^11^ to be associated with migraine (rs10456100, OR = 1.051, *P* = 9.2 × 10^−19^), but is uncorrelated with rs72854118 (*r*^2^ = 0.002). rs72854118-G is reported in weak association with decreased diastolic blood pressure (*β* = −0.07, *P* = 2.7 × 10^−7^)^35^, and in a GWAS meta-analysis of self-reported migraine and headaches combined, one of two correlated SNPs, rs72854120-C, shows borderline association, more so with headaches than migraine (*Z*_migraine_ = −2.68, *Z*_headache_ = −5.49, *P* = 2.8 × 10^−8^)^36^. Inspection of effect–effect plots of BRH versus clinically defined migraine for all 44 lead variants shows that rs72854118-G effects on BRH far exceed its migraine effects (Fig. 4 and Supplementary Fig. 5). We performed a phenoscan in 1,000 GWAS meta-analyses at deCODE Genetics (*P* threshold = 0.05/1,000 = 5.0 × 10^−5^) and observed that rs72854118-G also confers substantial protection against brain aneurysms (OR = 0.470, *P* = 1.8 × 10^−8^) and coronary artery disease (CAD) requiring bypass surgery (OR = 0.725, *P* = 9.3 × 10^−8^), but associates more weakly with CAD in general (OR = 0.900, *P* = 1.9 × 10^−5^) and systolic blood pressure (effect = −0.054 s.d., *P* = 2.0 × 10^−5^; Supplementary Table 15). Of 17 known brain aneurysm variants^37^, 3 are in migraine loci (*FHL5*, *SLC24A3* and *PLCE1*). Plotting effects of the brain aneurysm variants (including rs72854118) on brain aneurysms versus effects on migraine and BRH, we find this variant is an outlier in both and confers larger protective effects against brain aneurysms than other brain aneurysm variants (Supplementary Fig. 5).

### Colocalization highlights new migraine and aura genes

We performed systemic functional annotation of the 44 lead variants and variants in high LD (*r*^*2*^ ≥ 0.8) and studied their association with mRNA sequence data (expression quantitative trait loci (eQTL)) and with protein levels in plasma^38^ (protein quantitative trait loci (pQTL); Methods; Supplementary Tables 16–19). Results are summarized in Supplementary Fig. 6. For the lead variants, we find 144 eQTLs, of which 16 implicate a specific gene (Supplementary Table 17). Variant rs4768221-G, in complete LD with rs10748014-T (VD association OR = 1.073, *P* = 1.2 × 10^−12^) upstream of *LRRK2*, consistently associates with VD and is the top ranking eQTL for this gene in blood. The allele associated with increased risk of VD associates with reduced *LRRK2* expression in blood (*β* = −0.74 s.d., *P* = 1.3 × 10^−1,260^).

The lead BRH variant near *KCNK5* rs72854118, but not the other correlated variants at this locus, is found within a distal enhancer-like sequence (dELS) as defined by ENCODE’s catalog of candidate *cis*-regulatory elements^39^, and the gene target for this regulatory element is *KCNK5* (Supplementary Tables 20 and 21 and Supplementary Note 3). The variant is too rare to be studied in Genotype-Tissue Expression (GTEx, which includes only three carriers; Supplementary Fig. 7), and its expression coverage in tissues available to us is too low for conclusive results.

Three variants (or variants with *r*^2^ ≥ 0.8) represent top *cis* pQTLs at their respective loci in Icelandic SomaScan plasma protein association data and two variants in the UK Olink data (Supplementary Table 19). These proteomic methods differ in protein profiles, but in both datasets are pQTL variants correlating with the migraine variant rs1359155039-TAAAAAAAAA upstream of *LATS1* that associates with reduced migraine risk and increased LRP11 plasma levels (*β* = 0.58 s.d., *P* = 10^−1,140^ and *β* = 0.59 s.d., *P* = 10^−2,140^ in Iceland and UK, respectively). LRP11 is predicted to be located in plasma membrane and involved in several processes, including response to heat and cold (https://www.ncbi.nlm.nih.gov/gene).

We do not have RNA expression or protein data for enough carriers of the rare *PRRT2* variants to detect transcription or protein associations. However, on the basis of previous functional studies^40^, the gene’s known function as a key component of the Ca^2+^-dependent neurotransmitter release machinery^41^, and its reported links to rare paroxysmal brain disorders including infantile convulsions, the movement disorder PKD and FHM^42^, in addition to the findings in this current study, we conclude that *PRRT2* is also a risk gene for the common forms of MA and epilepsy. Finally, we scanned the GWAS catalog (https://www.ebi.ac.uk/gwas/) for associations with lead variants identified in this study (or *r*^2^ ≥ 0.8). Results are presented in Supplementary Table 12.

### Pathway analysis highlights NGF-related processes

For the 22 genes with evidence supporting their role in migraine or subtypes, we performed a protein network analysis (https://reactome.org). Among the top 67 relevant pathways identified, 13 involve NGF processing, including TrkA activation by NGF, previously studied in the context of pain and pain therapeutics^43^. Interestingly, pathways involved in phase-4 resting potential and cardiac conduction involve the products of both *KCNK5* and *SCN11A*, with the products of both *LRRK2* and *LRPI* interacting in the cardiac conduction pathway (Supplementary Data and Supplementary Table 22).

### Genetic drug target analysis

We performed a genetic drug target analysis for the 22 genes for which we have evidence of function pointing to the gene in addition to the established MA gene *CACNA1A*. Drugs at various levels of development target four genes that associate with MA (*PRRT2*, *ABO*, *LRRK2* and *CACNA1A*), none associated with MO, and four genes that associate with overall migraine or severe headaches (*KCNK5*, *NGF*, *SCN11A* and *TRPM8*; Supplementary Table 23 and Supplementary Note 5). Targeting *PRRT2* is bryostatin, a powerful protein kinase C agonist that was originally developed to prevent tumor growth, but in preclinical studies has also shown promising effects as a restorative synapse drug that is currently in trials to treat Alzheimer’s disease^44^. Several voltage-gated Ca^+2^ channel blockers have been developed against *CACNA1A*, but have not been tested in migraine. Targeting *TRPM8*, cutaneous menthol treatment has been found to alleviate migraine headaches^45^. Targeting *SCN11A* (and other voltage-gated sodium transporter genes), intranasal lidocaine can be effective in treating acute migraine^46^, and intravenous lidocaine infusion is suggested for treating refractory chronic migraine^47^. Drugs targeting other genes have not been tested for migraine, but β-nerve growth factor inhibitors (antibodies) that target *NGF* (fasinumab, tanezumab and fulranumab) are widely studied in the context of various other chronic pain conditions (for example, sciatica, low back pain and abdominal pain; www.ClinicalTrials.gov).

## Discussion

Whether MA and MO are different diseases or part of a migraine continuum has long been debated^48,49^. Little is known about the genetics underlying migraine subtypes as most prior studies have focused on migraine in general. Here we have identified several new associations supporting the distinct pathogenesis of MA and MO. In terms of MA, variants in *PRRT2*, *PALMD*, *CACNA1A*, *ABO* and *LRRK2* associate with MA (VD) over MO. Of these, two genes have the highest expression in the cerebellum (*PRRT2* and *CACNA1A*), and in both are rare autosomal dominant variants reported to cause rare forms of movement disorders and hemiplegic migraine (https://www.omim.org/). This is of interest in light of the characteristic cortical spreading depression observed in MA but not MO^4,5^. Both *ABO* and *PALMD* are widely expressed in tissues, and both harbor variants associated with cardiovascular disorders. Indeed, the link between migraine and cardiovascular disease is well established^50^. Drugs targeting these genes are in various phases of development, but for indications other than migraine. Five drugs target *CACNA1A* for seven indications, including anxiety, insomnia and cardiovascular disease, and targeting *LRRK2* is a trial drug DNL201 (ClinicalTrials.gov identifier: NCT0371070, https://clinicaltrials.gov/study/NCT03710707) that shows promising therapeutic potential against PD^51^. LRRK2 is especially abundant in dopamine-innervated areas and dopaminergic neurons of the substantia nigra^30^. Increased LRRK2 kinase activity is thought to impair lysosomal function and thus contribute to the pathogenesis of PD^52^. However, consistent with our results showing that the variant in *LRRK2* associates with increased risk of VD (MA) and with reduced LRRK2 mRNA expression, the main adverse effects of this LRRK2 inhibitor in healthy individuals were headache (40% of participants) and nausea (13%), the main symptoms of migraine, and dizziness (in 13%)^51^. While *LRRK2*’s expression is highest in brain areas associated with PD pathology, it is also expressed in other neurons and glial cells of the human brain^53^. Considerable pleiomorphism can occur among *LRRK2* carriers sharing the same pathogenic variant, even within the same family^54^. Indeed, *LRRK2* has been dubbed the ‘Rosetta stone’ of Parkinsonism, perhaps providing a common link between various neurological diseases^55^.

Our GWAS meta-analysis identified six variants associated with MO, all in previously reported migraine loci. However, by the subtype stratification of all lead variants, we detect 13 variants that impact MO over MA. These MO-associated variants are in or near genes with various functions, such as muscle cell development and differentiation (*MEF2D*, *FGF6* and *LRP1*) and intracellular calcium homeostasis (*MRVI1* and *SLC24A3*). Several are in genes highly expressed in arteries (*MEF2D*, *LRP1*, *ADAMTSL4*, *SUGCT*, *MRVI1* and *MRPS6*) and in brain (*MEF2D*, *ARAP2*, *PHACTR1* and *SLC24A3*). Of these, only *LRP1* is currently a drug target (https://platform.opentargets.org). *LRP1* encodes low-density lipoprotein receptor-related protein 1, and an LRP1 binding agent is in trials to treat various brain tumors.

Our results highlight three genes in or near which rare variants show large and informative effects. Firstly, the rare insertion (p.Arg217ProfsTer8) in *PRRT2* that associates with large effects on epilepsy and MA provides new insights into these comorbid^56^ and genetically correlated diseases. *PRRT2* is a four-exon gene that encodes a 340 amino acid protein with two predicted transmembrane domains^25^. Both the insertion and rarer deletion lead to premature termination of around one-third of PRRT2, resulting in nonsense-mediated decay^40^. Due to the founder effect in Iceland, we have power to show the pleiotropic effect of these LOF variants. Not only can they lead to rare neurological disorders, but they also confer substantial risk of common forms of MA and epilepsy, both of which are paroxysmal brain diseases frequently experienced with aura^57,58^. *PRRT2* is widely expressed in the brain, particularly in the cerebellum^25,59^. It is enriched in presynaptic terminals, is regulated by Ca^+2^ release and interacts with SNAP-25 and synaptogamin^41^. The mutant PRRT2 of the truncating variants leads to increased glutamate release and subsequent neuronal hyperexcitability^60^. A study of three Na_v_1 subunits (Na_v_1.1 encoded by *SCN1A*, Na_v_1.2 encoded by *SCN2A* and Na_v_1.6 encoded by *SCN8A*) expressed in human embryonic kidney cell lines (HEK-293) demonstrated that PRRT2 directly interacts with and negatively modulates Na_v_1.2 and Na_v_1.6, which generate action potentials in excitatory neurons, but does not affect Na_v_1.1 channels, which generate action potentials in inhibitory neurons^61^. Lack of PRRT2 leads to hyperactivity of Na_v_1.2 and Na_v_1.6 in homozygous *PRRT2* knockout (human and mouse) neurons^61^. The authors of that study suggest that the lack of PRRT2 effects on Na_v_1.1 may enhance excitation/inhibition imbalance and trigger hyper-synchronized activity in neuronal networks^61^. Interestingly, we find that the only epilepsy variant in our data that also associates with migraine is rs59237858 in *SCN1A*, the gene that encodes Na_v_1.1.

Secondly, in the context of Na_v_1 channels, it is of interest that we find both common and rare variants in *SCN11A* that impact migraine risk. *SCN11A* encodes Na_v_1.9 that is expressed in primary sensory neurons in peripheral and trigeminal ganglia^62^ and is known to have a substantial role in pain perception^62^. Compared to other sodium channels, Na_v_1.9 generates a persistent current regulated by G-protein pathways^63^. Whether Na_v_1.9 is also affected by *PRRT2*, like Na_v_1.2 and Na_v_1.6 (ref. ^61^), is not known. Currently in various stages of development are 63 drugs targeting *SCN11A* (most unspecific blockers of all Na_v_ subtypes), with 341 indications, including headache, epilepsy and pain in general (https://genetics.opentargets.org/gene/ENSG00000168356). Increasing specificity of Na_v_ subtype channel blockers and studying their protein interactions seems key to harnessing their therapeutic potential^64,65^.

Thirdly, the rare intergenic rs72854118-G near *KCNK5* and *KCNK17* is another variant providing insight into the pathogenesis of migraine. Previous studies have assigned this variant to *KCNK17* and reported weak associations with reduced blood pressure^35^ and protection against self-reported headaches and migraine^36^. However, we find that rs72854118, but not its correlated variants at this locus, is in a *cis*-regulatory region targeting *KCNK5*. *KCNK5* encodes TWIK-related acid-sensitive potassium channel 2, primarily expressed in kidney (GTEx, https://gtexportal.org) but also in T cells, suggesting a role in the immune system^66^. We find that the variant also confers protection against brain aneurysms and severe occlusive CAD, but associates weakly with blood pressure. Although hypertension is a risk factor for both aneurysms and CAD, it is not a conclusive risk factor for migraine^67^. The observed association with brain aneurysms begs the question whether in some cases undetected brain aneurysms could be misclassified as migraine^68^. According to the Open Targets Platform, no drugs are in development that target *KCNK5*.

In all, our findings are consistent with the results of previous GWAS analyses that have established migraine as a complex neurovascular brain disorder^13,69^. However, our results also highlight several distinct biological pathways involved in MA and MO that warrant further study. In summary, we contribute new insights into both general and specific mechanisms underlying migraine and its subtypes, especially to the visual aura associated with migraine attacks. Our results also emphasize the importance of assessing disease subtypes and proxies to improve understanding of complex genetic signals.

## Methods

### Ethics statement

All human research was approved by the relevant ethics review boards and conducted according to the Declaration of Helsinki. All participants provided written and informed consent as described per the study population below.

### Study populations

Cases and controls were defined from six study populations.

#### Iceland

About 155,000, or close to half of the Icelandic population of 340,000, have participated in an ongoing nationwide research program at deCODE Genetics^71,72^. Participants donated blood or buccal samples after signing informed consents allowing the use of their samples and data in various studies approved by the National Bioethics Committee (NBC). The data used here were analyzed under a study on the genetics of migraine (NBC; 19-158-V3, VSNb2019090003/03.01) following review by the Icelandic Data Protection Authority.

#### Denmark

Danish samples and data were obtained in collaboration with the Copenhagen Hospital Biobank Study^15^ and the DBDS^16^. CHB is a research biobank, which contains samples obtained during diagnostic procedures on hospitalized and outpatients in the Danish Capital Region hospitals. Data analysis within this study was performed under the ‘Genetics of pain and degenerative diseases’ protocol, approved by the Danish Data Protection Agency (P-2019-51) and the National Committee on Health Research Ethics (NVK-18038012). The DBDS Genomic Cohort is a nationwide study of ~110,000 blood donors^16^. The Danish Data Protection Agency (P-2019-99) and the National Committee on Health Research Ethics (NVK-1700407) approved the studies under which data on DBDS participants were obtained for this study.

#### UK

Since 2006, the UK Biobank resource has collected extensive phenotype and genotype data from ~500,000 participants recruited in the age range of 40–69 from across the UK after signing an informed consent for the use of their data in genetic studies^17^. The North West Research Ethics Committee reviewed and approved the UK Biobank’s scientific protocol and operational procedures (REC Reference: 06/MRE08/65). This study was conducted using the UK Biobank Resource (application 42256).

#### Finland

The FinnGen study^20^ consists of samples collected from the Finnish biobanks and phenotype data collected at Finland’s national health registers. The Coordinating Ethics Committee of the Helsinki and Uusimaa Hospital District evaluated and approved the FinnGen research project. The project complies with existing legislation (in particular the Biobank Law and the Personal Data Act). The official data controller of the study is the University of Helsinki. The summary statistics for FinnGen’s migraine GWAS were imported from a source available to consortium partners (Release 6: https://r6.finngen.fi/).

#### US

Participants from the US were recruited via ongoing studies conducted at Intermountain Healthcare (https://intermountainhealthcare.org). These studies include the Intermountain Inspire Registry and the HerediGene: Population study^18^. The latter is a large-scale collaboration between Intermountain Healthcare, deCODE Genetics and Amgen. The Intermountain Healthcare Institutional Review Board approved this study, and all participants provided written informed consent and samples for genotyping.

#### Norway

Data on Norwegian migraine cases and controls were obtained from the HUSK study, a population-based study carried out in Hordaland county in Western Norway^19^. In 1992–1993, all Hordaland County residents born between 1950 and 1952, all Bergen residents born between 1925 and 1927 and three neighboring municipalities and a random sample of individuals born between 1926 and 1949 were invited to participate. In total, 18,044 individuals participated, of which 17,561 provided blood samples for genotyping, of which 10,000 were genotyped at deCODE Genetics. All participants signed informed consents, and the study was approved and carried out by the National Health Screening Service, Oslo (now the Norwegian Institute of Public Health) in cooperation with the University of Bergen^19^.

### Phenotype definitions

Cases with migraine and the migraine subtypes with and without aura were in all cohorts but Norway (using self-reported migraine from questionnaires), mainly defined by International Classification of Diseases 10th Revision (ICD-10) codes (or comparable codes from earlier versions of ICD) representing MA (code G43.1, MO (G43.0) and overall migraine (G43). Diagnostic codes were assigned by physicians and captured through both inpatient and outpatient diagnostic registries. As triptan medications (Anatomical Therapeutic Chemical code N02CC) are used to prevent/treat migraine attacks, individuals who had received triptan subscriptions were identified in data from drug registries (Iceland, Denmark, Finland and the UK) and added to migraine cases (without subtype).

Both proxy phenotypes used in this study were based on validated questionnaire items selected for the headache section of UK Biobank’s pain questionnaire (https://biobank.ctsu.ox.ac.uk/crystal/ukb/docs/pain_questionnaire.pdf), which was designed in consultation with a group of leaders in pain research. The headache section is based on questions used in the American Migraine Prevalence and Prevention study^73^. For the MA-proxy phenotype used in this study (VD preceding headaches), we defined cases and controls from questionnaire data obtained in the studies conducted in Iceland, Denmark and the UK Biobank. Questions used in Icelandic and Danish cohorts were comparable to the question answered by participants in the UK Biobank (data field 120065: data description: visual changes before or near the onset of headaches, Question: ‘I develop visual changes such as spots, lines and heat waves or graying out of my vision’). Responses ‘Yes’ were compared to responses ‘No.’ Such defined cases with, and controls without, headache-related VD had all previously responded ‘Yes’ to a question on headaches as asked in the UK Biobank survey (data field 120053: data description: bad and/or recurring headaches at any time in life, Question: ‘Have you ever had bad and/or recurring headaches at any time in your life?’). We used this UK Biobank data field 120053 as a migraine proxy, defining comparable severity qualified headache questions in Icelandic and Danish questionnaire datasets for the GWAS meta-analysis.

### Genotyping and whole-genome sequencing

#### Iceland

At deCODE Genetics, 63,118 Icelandic samples have been whole-genome sequenced (WGS) using GAIIx, HiSeq, HiSeqX and NovaSeq Illumina technology^71,72^ to a mean depth of 38×. Genotypes of single-nucleotide polymorphisms (SNPs) and insertions/deletions (indels) were identified and called jointly by Graphtyper^74^. The effects of sequence variants on protein-coding genes were annotated using the variant effect predictor (VEP) using protein-coding transcripts from RefSeq. Including all sequenced samples, 155,250 samples from Icelandic participants have been genotyped using various Illumina SNP arrays^71,72^. The chip-typed individuals were long-range phased^75^, and the variants identified in the WGS Icelanders imputed into the chip-typed individuals. Additionally, genotype probabilities for 285,644 ungenotyped close relatives of chip-typed individuals were calculated based on extensive encrypted genealogy data compiled by deCODE Genetics (an unencrypted version is publicly available to all Icelandic citizens at https://www.islendingabok.is/english). All variants tested were required to have imputation information over 0.8.

#### Denmark

Danish samples from both CHB and DBDS were genotyped at deCODE Genetics using Illumina Infinium Global Screening Array. Individual genotype arrays were discarded if the total yield was below 98%. Variants were derived from sequencing 25,215 Scandinavian samples (8,360 Danish) using NovaSeq Illumina technology. Only samples with a genome-wide average coverage of over 20× were used. The genotypes of SNPs and indels were called jointly by Graphtyper^74^. Variants with a missing rate >2% were discarded. The genotyped samples were phased using Eagle (version 2.4.1) and high-quality variants imputed into 270,627 genotyped Danes using haplotype sharing in a Hidden Markov Model based on a Li and Stephens model^76^ similar to the one used in IMPUTE2 (ref. ^77^).

#### UK

In the UK Biobank dataset, the first 50,000 participants were genotyped using a custom-made Affymetrix chip, UK BiLEVE Axiom^78^, and the remaining participants using the Affymetrix UK Biobank Axiom array^17^. We used existing long-range phasing of the SNP chip-genotyped samples^17^. We excluded SNP and indel sequence variants in which at least 50% of samples had no coverage (genotype quality (GQ) score = 0), if the Hardy–Weinberg *P* value was <10^−30^ or if heterozygous excess <0.05 or >1.5. At deCODE Genetics, a collaborative effort was recently performed to whole-genome sequence 150,119 samples from the UK Biobank, allowing us to create a haplotype reference panel, which was then imputed into the UK Biobank chip-genotyped dataset, as previously described elsewhere^79^.

#### US

Samples from the US (Intermountain dataset) were genotyped using Illumina Global Screening Array chips (*n* = 28,279) and WGS using NovaSeq Illumina technology (*n* = 16,621). Samples were filtered on 98% variant yield and any duplicates were removed. Over 245 million high-quality sequence variants and indels, sequenced to a mean depth of 20×, were identified using Graphtyper^74^. Quality-controlled chip genotype data were phased using SHAPEIT4 (ref. ^80^). A phased haplotype reference panel was prepared from the sequence variants using the long-range phased chip-genotyped samples using in-house tools and methods described previously^71,72^.

#### Norway

Norwegian samples were genotyped on Illumina SNP arrays (OmniExpress or Global Screening Array). The chip-genotyping QC and imputation of the Norwegian dataset were performed at deCODE Genetics in Iceland using the same methods as described above for the Icelandic samples. The imputation for Norwegian samples is based on a haplotype reference panel of 25,215 samples of European ancestry, of which 3,336 are Norwegian.

#### Finland

A custom-made FinnGen ThermoFisher Axiom array (>650,000 SNPs) was used to genotype FinnGen samples at the Thermo Fisher Scientific genotyping service facility in San Diego. Genotype calls were made with the AxiomGT1 algorithm (https://finngen.gitbook.io/documentation/methods/genotype-imputation). The FinnGen Release 6 used in this study contains 260,405 genotyped individuals after quality control (QC). Individuals with ambiguous sex, high genotype missingness (>5%), excess heterozygosity (±4 s.d.) or non-Finnish ancestry were excluded, as were variants with high missingness (>2%), low Hardy–Weinberg equilibrium (<1 × 10^−6^) or minor allele count (<3). Imputation was performed using the Finnish population-specific and high coverage (25–30 times) WGS backbone and the population-specific SISu v3 imputation reference panel with Beagle 4.1. More than 16 million variants have been imputed in the Finnish dataset (https://www.finngen.fi/en/access_results).

### Genetic ancestry filtering and principal components

For the UK Biobank, we used a British–Irish ancestry subset defined previously^79^. Procedures to account for ancestry in FinnGen^20^ and Iceland^72^ have also been previously described. Genetic ancestry analysis to identify subsets of individuals with similar ancestry was performed for the Danish, Intermountain and Norwegian datasets separately. ADMIXTURE (v1.23)^81^ was run in supervised mode using the 1000 Genomes populations^82^ CEU (Utah residents with Northern and Western European ancestry), CHB (Han Chinese in Beijing, China), ITU (Indian Telugu in the UK), PEL (Peruvian in Lima, Peru) and YRI (Yoruba in Ibadan, Nigeria) as training samples. These training samples had themselves been filtered for ancestry outliers using principal component analysis (PCA) and unsupervised ADMIXTURE.

For the Danish and Intermountain datasets, samples assigned <0.93 CEU were excluded. We performed a different filtering procedure for the Norwegian dataset to include individuals with Finnish and Saami ancestry, who are common in Norway^83^. To identify such individuals, we first selected candidates those assigned between 0.5 and 0.93 CEU ancestry. We then merged these individuals with the Human Origins dataset and calculated *F* statistics^84^ of the form *f*_3_ (Mbuti; candidate individual, X), where X was each of the Human Origins populations Nganasan, Pima, Han and Norwegian. In these *F*_3_ statistics, we identified a clear cluster of individuals with excess affinity to Nganasan and Norwegian over Pima and Han. In available metadata, we observed that these individuals were highly enriched for locations of residence in Finnmark and officially designated Saami villages. These genetic and demographic features match expectations for individuals of Saami or Finnish ancestry. Except for this cluster, we excluded all other Norwegian individuals assigned <0.93 CEU ancestry. Genetic principal components for use as covariates in association analysis were obtained using bigsnpr^85^.

### Association testing and meta-analysis

Using software developed at deCODE Genetics^72^, we applied logistic regression assuming an additive model to test for genome-wide associations between sequence variants and migraine phenotypes. Association results from FinnGen were imported (Release 6: http://r6.finngen.fi). For the Icelandic data, the model included sex, county of birth, current age or age at death (first-order and second-order terms included), blood sample availability for the individual and an indicator function for the overlap of the lifetime of the individual with the time span of phenotype collection. To include imputed but ungenotyped individuals, we used county of birth as a proxy covariate for the first PCs in our analysis because county of birth has been shown to be in concordance with the first PC in Iceland^86^. For the Danish, Norwegian, UK and US data, the covariates were sex, age, expected allele count and 20 PCs to adjust for population stratification. The association analysis of the imported Finnish data was adjusted for sex, age, the genotyping batch and the first ten PCs. We used LD score regression intercepts^22^ to adjust the *χ*^2^ statistics and avoid inflation due to cryptic relatedness and stratification, using a set of 1.1 million variants. *P* values were calculated from the adjusted *χ*^2^ results. All statistical tests were two-sided unless otherwise indicated.

For the meta-analyses, we combined GWASs from the respective cohorts with summary statistics from Finland using a fixed-effects inverse-variance method based on effect estimates and s.e. in which each dataset was assumed to have a common OR but allowed to have different population frequencies for alleles and genotypes. The total number of variants included in the meta-analyses was between 68 and 80 million variants. Sequence variants were mapped to the NCBI Build 38 and matched on position and alleles to harmonize the datasets. The threshold for genome-wide significance was corrected for multiple testing with a weighted Bonferroni adjustment that controls for the family-wise error rate, using as weights the enrichment of variant classes with predicted functional impact among association signals^21^. The significance threshold then becomes 2.5 × 10^−7^ for high-impact variants (including stop-gained, frameshift, splice acceptor or donor), 5.0 × 10^−8^ for moderate-impact variants (including missense, splice-region variants and in-frame indels), 4.5 × 10^−9^ for low-impact variants, 2.3 × 10^−9^ for other DNase I hypersensitivity sites (DHS) variants and 7.5 × 10^−10^ for other non-DHS variants^21^. In a random-effects method, a likelihood ratio test was performed in all genome-wide associations to test the heterogeneity of the effect estimate in the four datasets; the null hypothesis is that the effects are the same in all datasets, and the alternative hypothesis is that the effects differ between datasets.

The primary signal at each genomic locus was defined as the sequence variant with the lowest Bonferroni-adjusted *P* value using the adjusted significance thresholds described above. Conditional analysis was used to identify possible secondary signals within 500 kb from the primary signal. This was done using genotype data for the Icelandic, Norwegian, Danish, UK and US datasets and an approximate conditional analysis implemented in GCTA software^87^ for the Finnish summary data. Adjusted *P* values and ORs were combined using a fixed-effects inverse-variance method. Class-specific genome-wide significance thresholds were also used for the secondary signals. Manhattan plots were generated using topr package in R.

For burden testing, we used the UK Biobank whole-exome sequenced dataset, consisting of 400,912 whole-exome sequenced White British (individuals identified by PCA analyses)^88,89^ who enrolled in the study between 2006 and 2010 throughout the UK and were aged 38–65 years at recruitment. A wide range of phenotypic data has been provided by the UK Biobank primarily from hospital records and increasingly from general practitioners from the UK. For the Icelandic, US and Danish cohorts, we used the phenotypes and WGS and imputation data previously described.

We used VEP^90^ to attribute predicted consequences to the variants sequenced in each dataset. We classified as high-impact variants those predicted as start-lost, stop-gain, stop-lost, splice donor, splice acceptor or frameshift, collectively called LOF variants. For case–control analyses, we used logistic regression under an additive model to test for association between LOF gene burdens and phenotypes, in which disease status was the dependent variable and genotype counts as the independent variable, using likelihood ratio test to compute two-sided *P* values. Individuals were coded 1 if they carried any of the LOF variants in the autosomal gene being tested and 0 otherwise. For the UK Biobank association testing, 20 PCs were used to adjust for population substructure, and age and sex were included as covariates in the logistic regression model. We further included variables indicating sequencing batches to remove batch effects. For these analyses, we used software developed at deCODE Genetics^72^.

### Genetic correlations

Using cross-trait LD score regression^22^, we estimated the genetic correlation between each of the migraine and proxy (BRH) and migraine subtype phenotypes (MO, MA and VD) defined in this study, in addition to epilepsy. In this analysis, we used results for about 1.2 million well-imputed variants, and for LD information, we used precomputed LD scores for European populations (downloaded from https://data.broadinstitute.org/alkesgroup/LDSCORE/eur_w_ld_chr.tar.bz2). To avoid bias due to sample overlap, we used the Icelandic and Danish cohorts combined to test for correlation with the respective phenotypes in the other remaining datasets combined. Finally, we meta-analyzed the results of the two correlation analyses for each correlation for a combined correlation estimation. The significance level for the correlation estimates was determined using a simple Bonferroni correction for the number of meta-analyzed correlations, and hence significance was set at *P* < 0.0033 (0.05/15).

### Identification and confirmation of rare *PRRT2* variants

The variants in the *PRRT2* gene are in a stretch of nine C’s, with one extra C in carriers of the insertion (p.Arg217ProfsTer8) and one missing C in carriers of the deletion (p.Arg217GlufsTer12). This imposes a technical challenge for accurate whole-genome sequence calling. Therefore, all potential carriers of both variants were analyzed with Sanger sequencing. Primers were designed using Primer 3 software. Following PCR, cycle sequencing reactions were performed in both directions on MJ Research PTC-225 thermal cyclers, using the BigDye Terminator Cycle Sequencing Kit v3.1 (Life Technologies) and Ampure XP and CleanSeq kits (Agencourt) for cleanup of the PCR products and cycle sequencing reactions. Sequencing products were loaded onto the 3730 XL DNA Analyzer (Applied Biosystems) and analyzed with Sequencher 5.0 software (Gene Codes Corporation). Based on the sequencing results, the variants were then re-imputed into the respective cohorts.

### Migraine subtype analysis of lead variants

To classify our lead variants by migraine subtype, we plotted their effects on MA versus MO and VD versus MO using the method applied in ref. ^11^. This method requires a correlation parameter between MO and MA (MO and VD) to account for sample overlap, and previously this parameter was estimated from GWAS summary statistics^11^, using empirical Pearson correlation of effect size estimates of common variants (MAF > 0.05), which do not show a strong association with either of the migraine subtypes studied (*P* > 1 × 10^−4^)^91^. In our data, this estimate of the correlation parameter was *r*_*ij*_ = 0.59 between MO and MA and *r*_*ij*_ = 0.198 between MO and VD (estimated using 7,858,264 markers), which is considerably larger than if we estimated the sample overlap directly using counts of cases, controls and the counts of overlaps in these groups between phenotypes^70^ (from all cohorts except the summary statistics from FinnGen), where we get *r*_*ij*_ = 0.023 for MO and MA and *r*_*ij*_ = 0.012 for MO and VD. As the latter estimates are more conservative, we used those in the subtype analysis. Finally, we tested whether the effect sizes between MA and MO (and VD and MO) were equal at a Bonferroni corrected significance threshold of *P* = 0.05/43 (as we excluded from the 44 lead variants the MA variant in *PRRT2*) performed by using normal approximation and accounting for the correlation in effect size difference estimators. As pointed out in ref. ^11^, this subtype classification method takes into account the different statistical power of the migraine subtype GWASs, which is an advantage compared to simply comparing subtype effects. For the subtype analysis, we followed the R code available at https://github.com/mjpirinen/migraine-meta.

### Functional data and colocalization analysis

To highlight genes whose products potentially mediate the observed associations with migraine and migraine subtypes, we annotated the associations detected in this study (Tables 1 and 2) as well as variants in high LD (*r*^2^ ≥ 0.8 and within ±1 Mb) that are predicted to affect coding or splicing of a protein (VEP using RefSeq gene set), mRNA expression (top local eQTL, *cis*-eQTL) in multiple tissues from deCODE, GTEx (https://www.gtexportal.org) and other public datasets (see Supplementary Table 18 for eQTL data sources) and/or plasma protein levels (top pQTL) identified in large proteomic datasets from Iceland and the UK. The Icelandic proteomics data were analyzed using the SomaLogic SOMAscan proteomics assay that scans 4,907 aptamers, measuring 4,719 proteins in samples from 35,559 Icelanders with the genetic information available at deCODE Genetics^38^. Plasma protein levels were standardized and adjusted for year of birth, sex and year of sample collection (2000–2019)^38^. The UK proteomics dataset was analyzed using the Olink proteomics assay characterizing 1,463 proteins in 54,306 participants in the UK Biobank^92^.

RNA sequencing was performed on whole blood from 17,848 Icelanders and on subcutaneous adipose tissue from 769 Icelanders, respectively^38^. Gene expression was computed based on personalized transcript abundances using kallisto^93^. Association between sequence variants and gene expression (*cis*-eQTL) was tested using a generalized linear regression, assuming additive genetic effect and normal quantile gene expression estimates, adjusting for measurements of sequencing artifacts, demographic variables, blood composition and PCs^94^. The gene expression PCs were computed per chromosome using a leave-one-chromosome-out method. All variants within 1 Mb of each gene were tested.

We performed gene-based enrichment analysis using the GENE2FUNC tool in FUMA^95^. The genes were tested for over-representation in different gene sets, including Gene Ontology cellular components (MsigDB c5) and GWAS Catalog-reported genes.

### Genetic drug target analysis

Using sources from the Drug-Gene Interaction Database^96^, Open Targets^97^ and the National Institutes of Health’s Illuminating the Druggable Genome^98^, we performed a genetic drug target analysis for the 22 genes for which we have evidence of function pointing to the gene (Supplementary Fig. 6), in addition to the established MA gene *CACNA1A*.

### Reporting summary

Further information on research design is available in the Nature Portfolio Reporting Summary linked to this article.

## Online content

Any methods, additional references, Nature Portfolio reporting summaries, source data, extended data, supplementary information, acknowledgements, peer review information; details of author contributions and competing interests; and statements of data and code availability are available at 10.1038/s41588-023-01538-0.

### Supplementary information

Supplementary Information Supplementary Figs. 1–7 and Supplementary Notes 1–5.

Reporting Summary

Supplementary Tables Supplementary Tables 1–23.

Supplementary Data Reactome gene pathway report.

## Data Availability

Our previously described Icelandic population whole-genome sequence data have been deposited at the European Variant Archive under accession PRJEB15197. The GWAS summary statistics for the migraine GWAS meta-analyses are available at https://www.decode.com/summarydata/. FinnGen data are publicly available and were downloaded from https://www.finngen.fi/en/access_results. The UKB data were downloaded under application 42256. Proteomics data and protein mapping to UniProt identifiers and gene names were provided by SomaLogic and Olink. Other data generated or analyzed in this study are included in the article and its Supplementary Information. URLs for other external data used are as follows: precomputed LD scores for European populations, https://data.broadinstitute.org/alkesgroup/LDSCORE/eur_w_ld_chr.tar.bz2; GWAS Catalog, https://www.ebi.ac.uk/gwas/; GTEx project, https://gtexportal.org/home/. URL sources for expression data can be found in Supplementary Table 18.

## Code availability

We used publicly available software that is available on request under the following URLs: GraphTyper (v2.0-beta, GNU GPLv3 license), https://github.com/DecodeGenetics/graphtyper; Eagle (version 2.4.1), http://www.hsph.harvard.edu/alkes-price/software/; SHAPEIT4, https://odelaneau.github.io/shapeit4/; ADMIXTURE (v1.23), https://dalexander.github.io/admixture/; BOLT-LMM (v.2.1), http://www.hsph.harvard.edu/alkes-price/software/; R (version 3.6.3), https://www.r-project.org/; R package ggplot for visualization (version 3.3.3), https://ggplot2.tidyverse.org/; Ensembl v.87, https://www.ensembl.org/index.html; IMPUTE2 v.2.3.1, https://mathgen.stats.ox.ac.uk/impute/impute_v2.html; dbSNP v.140, http://www.ncbi.nlm.nih.gov/SNP/; kallisto v.0.46, https://github.com/pachterlab/kallisto; for subtype stratification analysis, we used R code available at https://github.com/mjpirinen/migraine-meta; MAGMA (v1.08), http://ctglab.nl/software/magma; VEP (release 100), https://github.com/Ensembl/ensembl-vep; FUMA, https://fuma.ctglab.nl/; Sequencher 5.0, https://sequencher.software.informer.com/5.0/; NCBI Build 38, https://www.ncbi.nlm.nih.gov/. No custom code was written for this study.
