# Supplementary material for: Rare variants with large effects provide functional insights into the pathology of migraine subtypes, with and without aura
Source: Nat Genet. 2023 Oct 26;55(11):1843–53. doi: 10.1038/s41588-023-01538-0 (PMC10632135; doi:10.1038/s41588-023-01538-0)
Supplement: Supplementary file 1 — Supplementary Figs. 1–7 and Supplementary Notes 1–5. [file 41588_2023_1538_MOESM1_ESM.pdf]

nature genetics

Article

https://doi.org/10.1038/s41588-023-01538-0

Rare variants with large effects provide functional insights into the pathology of migraine subtypes, with and without aura

In the format provided by the authors and unedited

Contents

Supplementary Figures 2

Figure 1 – Locus plots for *PRRT2* variant p.Arg217ProfsTer8 2

1a. Epilepsy GWAS in Iceland N = 6155/354672 2

1b. Migraine in Iceland N = 24604/319066 3

1c. Migraine with aura in Iceland (MA) N = 4640/307222 4

Figure 2 – Locus plots for novel variants 5

Figure 3 – Phenotypes studied and their genetic correlations 11

3a. Diagram of phenotypes studied and their relationships 11

3b. Genetic correlations 11

Figure 4 – Lead variants stratified by VD vs MA 12

Figure 5 – *KCNK5*, BRH and brain aneurysms 13

5a. Brain aneurysm variants and BRH effects. 13

5b. Lead variants, BRH and brain aneurysms 14

5c. Brain aneurysm effects vs migraine effects 15

Figure 6 – Genes likely to mediate effects 16

Figure 7 – KCNK5 expression by rs72854118 in GTEx 17

Supplementary Notes 18

1 – 123 migraine variants reported by Hautakangas et al. 18

2 – Results of *SCN11A* LOF burden test 19

3– Bad recurrent headache (BRH) variant and *KCNK5* 20

4 - FUMA Gene2Function analysis 22

a. Gene expression heatmap 24

b. GWAS catalogue reported genes 25

c. Differentially expressed genes (DEG) 26

d. Brainspan – 29 different ages of brain samples 27

e. Brainspan – 11 general developmental stages of brain samples 28

f. Brainspan – 11 general developmental stages of brain samples 29

5 - Genetic drug target analysis 30

Supplementary Figures

Figure 1 – Locus plots for *PRRT2* variant p.Arg217ProfsTer8

Regional association plots for p.Arg217ProfsTer8 at the 16p11.2 locus in association under the additive model, with **1a** epilepsy, **1b** migraine and **1c** migraine with aura (MA) in Icelandic data (N = Ncases/Nctrls). For all three plots, the x-axis shows the chromosomal location (NCBI Build 38 coordinates), the left y-axis shows the two-sided negative $\log_{10}$ (*P*) from the genome-wide association study (GWAS) results in the Icelandic dataset, a chi-squared test used to calculate *P*-values, that are corrected for multiple testing with a weighted Bonferroni adjustment using as weights the enrichment of variant classes with predicted functional impact among association signals[1]. The squared correlation (r^2) to the lead variant is shown by colors based on genetic correlation. The right y-axis shows calculated recombination rates at the chromosomal location plotted as a solid grey graph. Variants are colored to reflect their linkage disequilibrium (LD) with p.Arg217ProfsTer8 (chr16:29813694:IG, black diamond) in the dataset. Known genes in the region are shown underneath the plot, taken from the UCSC genes track in the UCSC Genome Browser (https://genome.ucsc.edu/).

1a. Epilepsy GWAS in Iceland N = 6155/354672

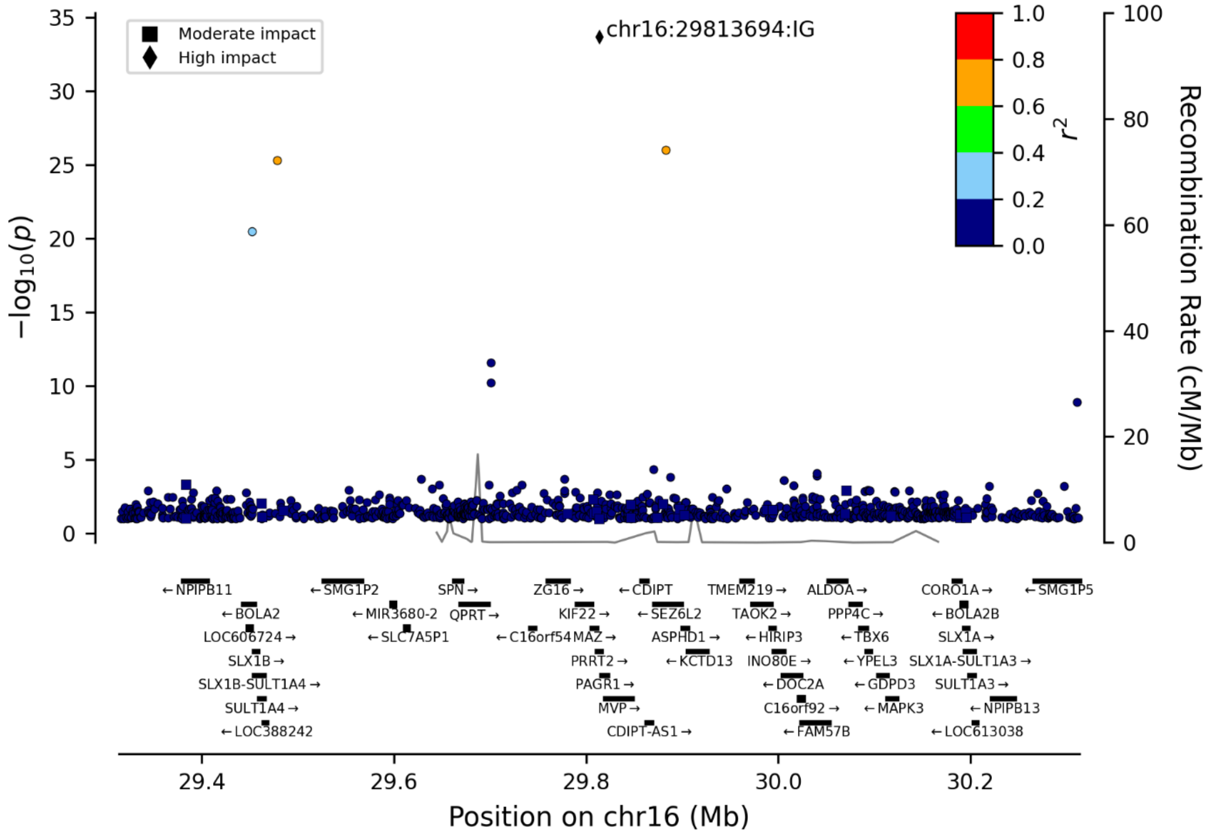

1b. Migraine in Iceland N = 24604/319066

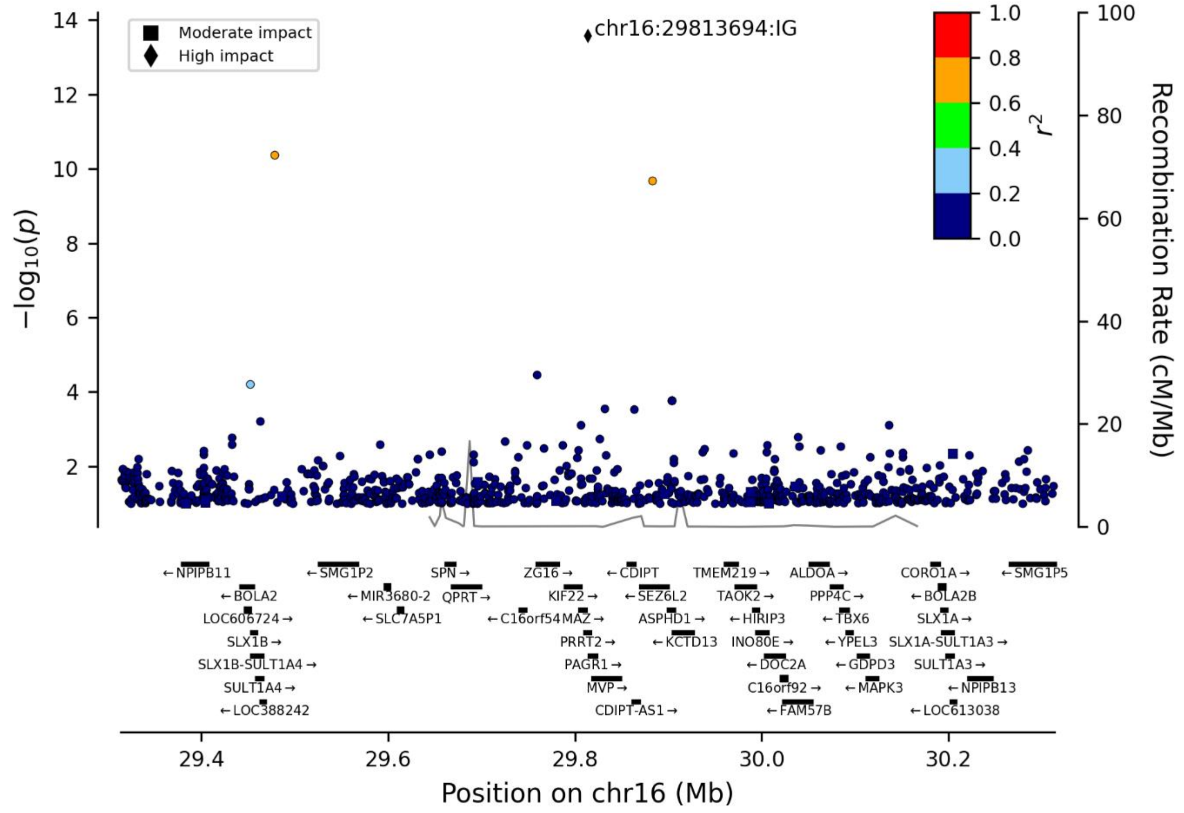

1c. Migraine with aura in Iceland (MA) N = 4640/307222

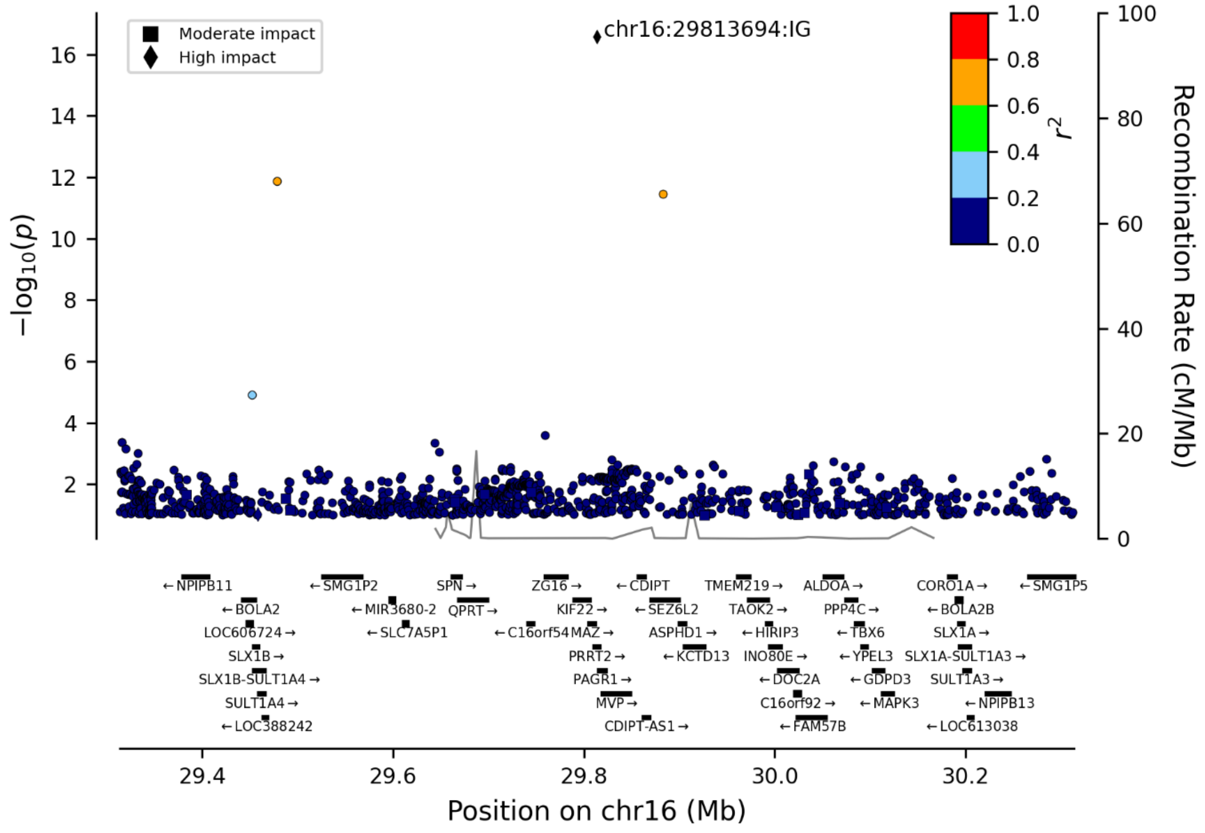

Figure 2 – Locus plots for novel variants

Regional association plots for novel variants associated in genome-wide meta-analyses under the additive model, for: Migraine (MIG) 6 cohorts $N_{case/ctrl}$ = 74,495/1,259,808; Migraine with aura (MA) 5 cohorts $N_{case/ctrl}$ = 16,603/1,336,517; Migraine without aura (MO) $N_{case/ctrl}$ = 11,718/1,330,747; Visual disturbances (VD) $N_{case/ctrl}$ = 30,297/86,134; and Bad recurrent headaches (BRH) $N_{case/ctrl}$ = 51,803/123,732. (See Supplementary Table 1 for $N_{case/ctrl}$ per cohort).

For all following plots, the x-axis shows the chromosomal location (NCBI Build 38 coordinates), the left y-axis shows the two-sided negative $\log_{10}$ (P) derived from a likelihood-ratio test. P-values are corrected for multiple testing with a weighted Bonferroni adjustment using as weights the enrichment of variant classes with predicted functional impact among association signals[1]. The squared correlation (r^2) to the lead variant is shown by colors based on genetic correlation. Grey background graph shows the recombination rate. Known genes in the region are shown underneath the plot, taken from the UCSC genes track in the UCSC Genome Browser (https://genome.ucsc.edu). All positions are in NCBI Build 38 coordinates.

<u>VD: rs11166276 in *PALMD* TF binding site (MAF = 49.0%)</u>

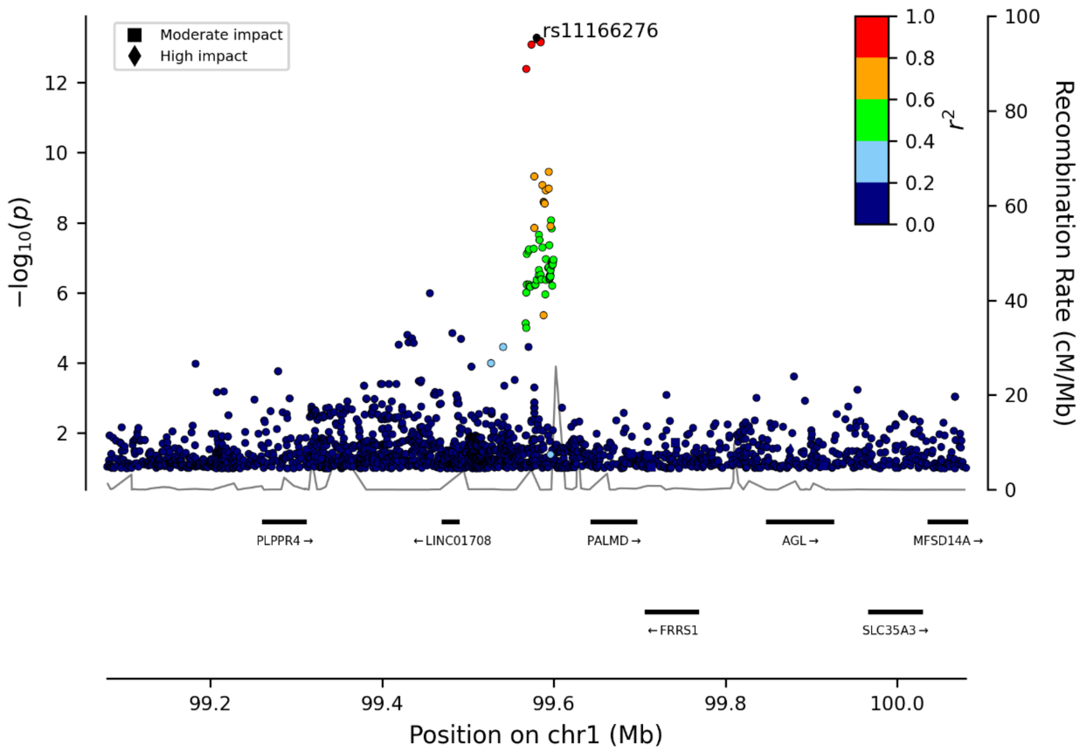

VD: rs77778288 in *HACD4/IFNB1* regulatory region (MAF = 12.9%)

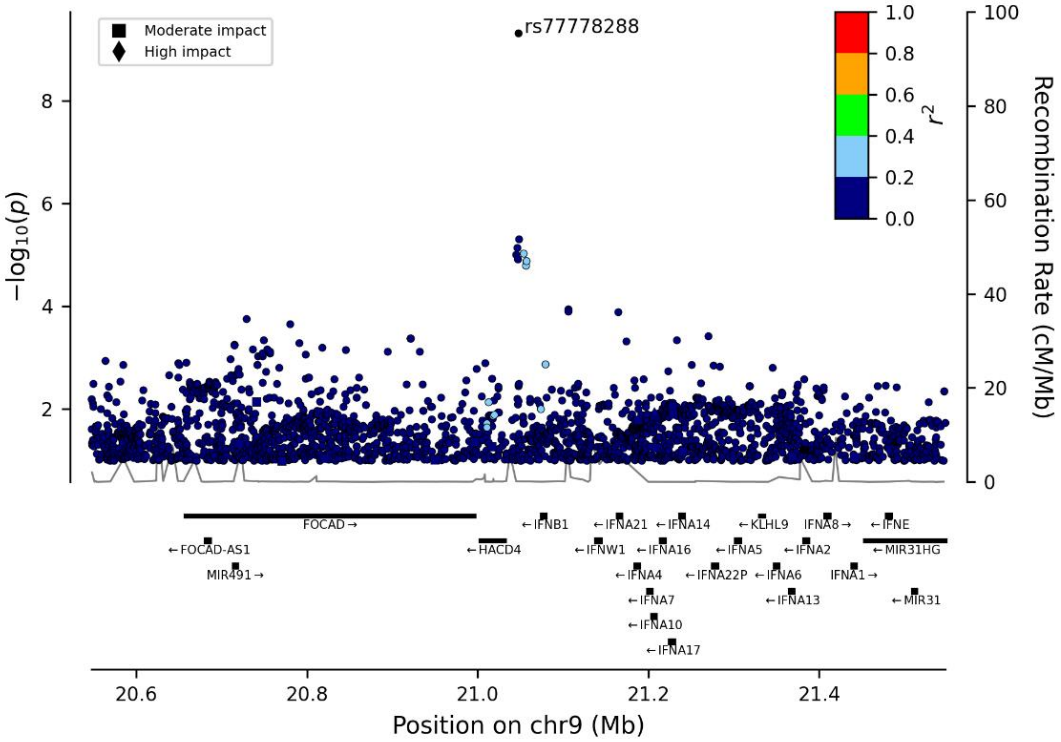

VD: rs8176719 *ABO* frameshift variant (MAF = 32.9%)

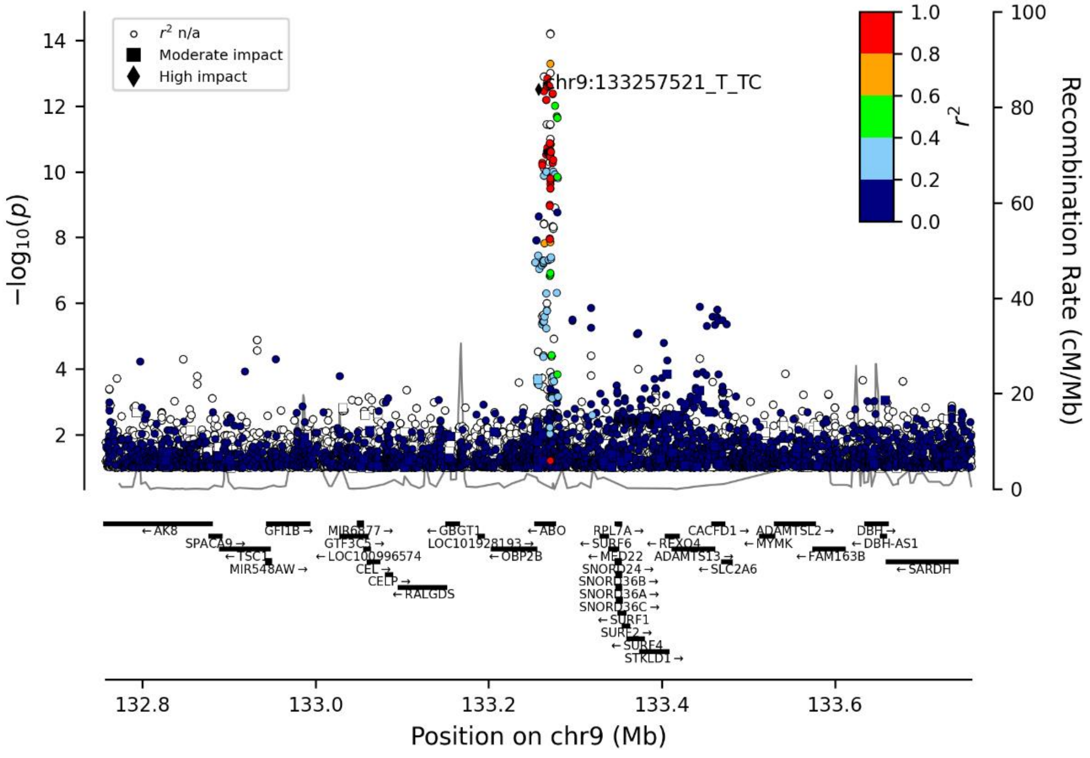

VD: rs10748014 *LRRK2* upstream variant (MAF = 46.8%)

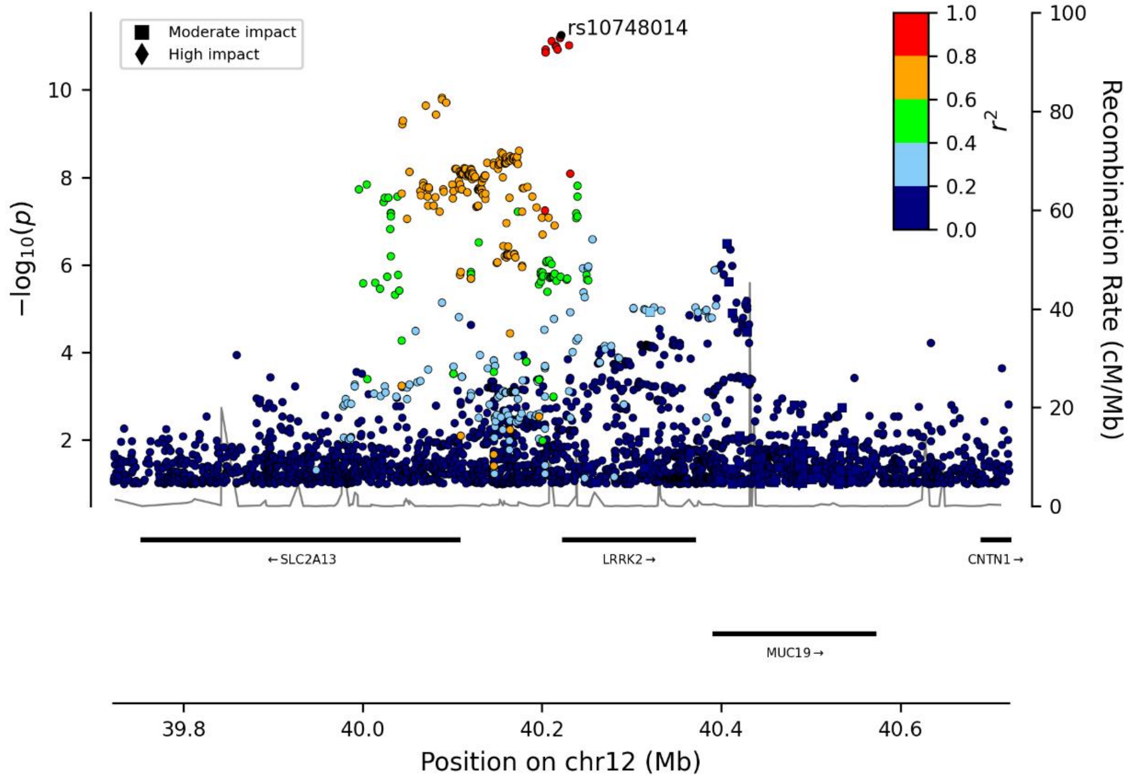

MIG: rs933718575 *A3GALT2/ZNF362* downstream variant (MAF = 0.01%), only in UKB data

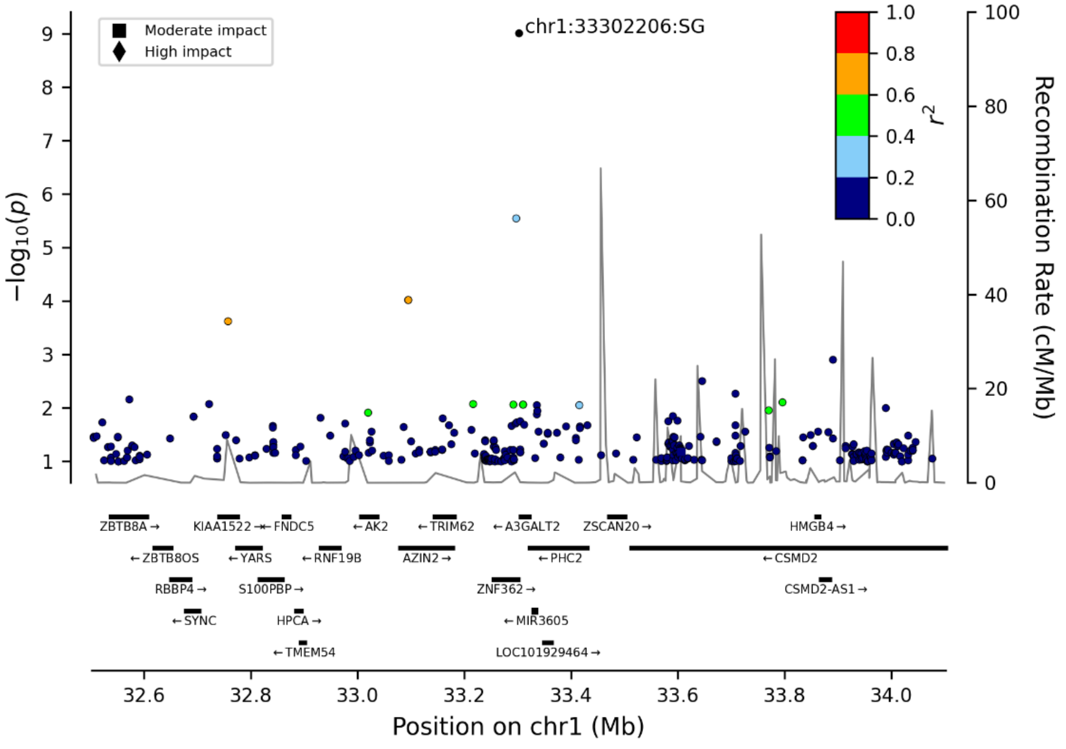

MIG: rs71642605 *MANEAL* upstream variant (MAF = 25.3%)

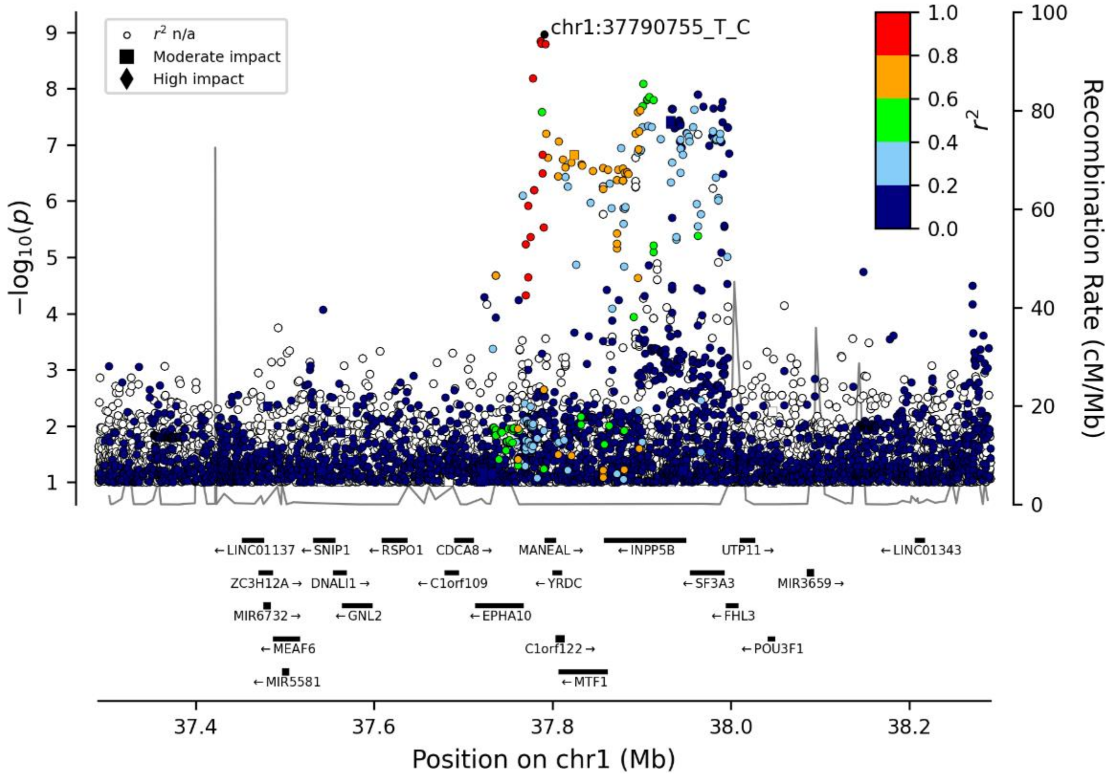

MIG: rs11799356 *PPCS* downstream variant (MAF = 34.2%)

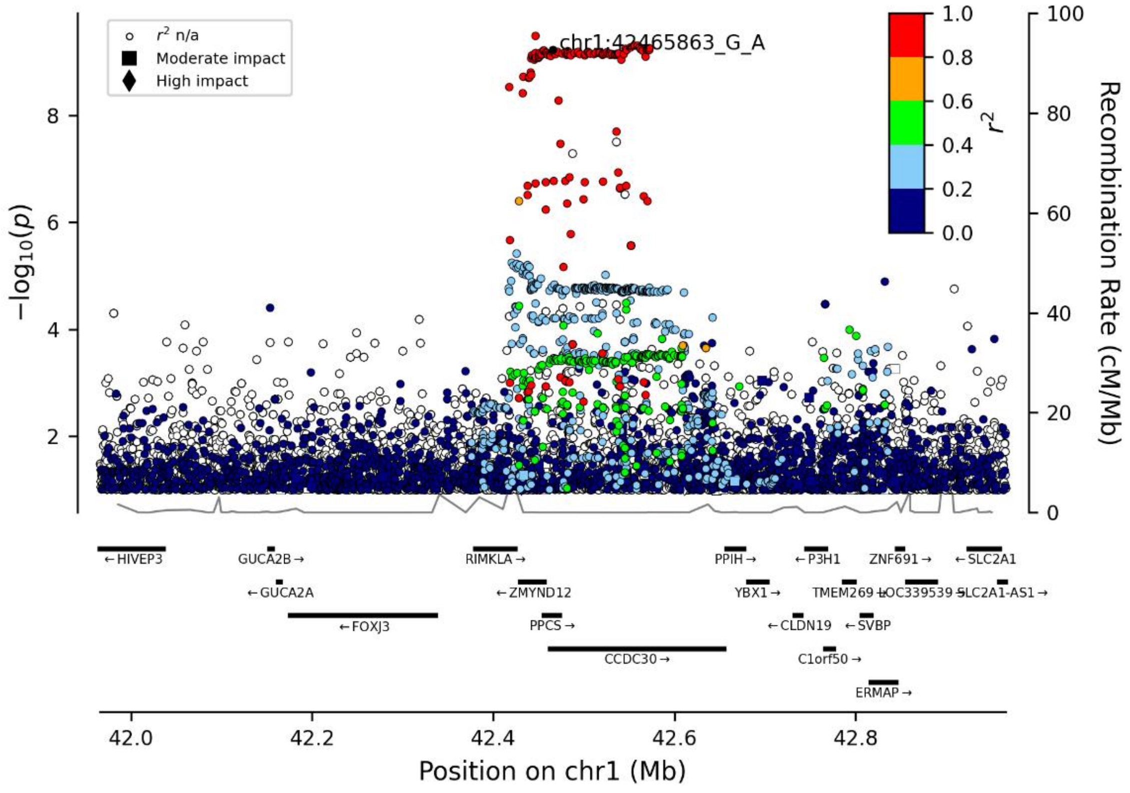

MIG: rs6330 *NGF* missense variant (MAF = 46.7%)

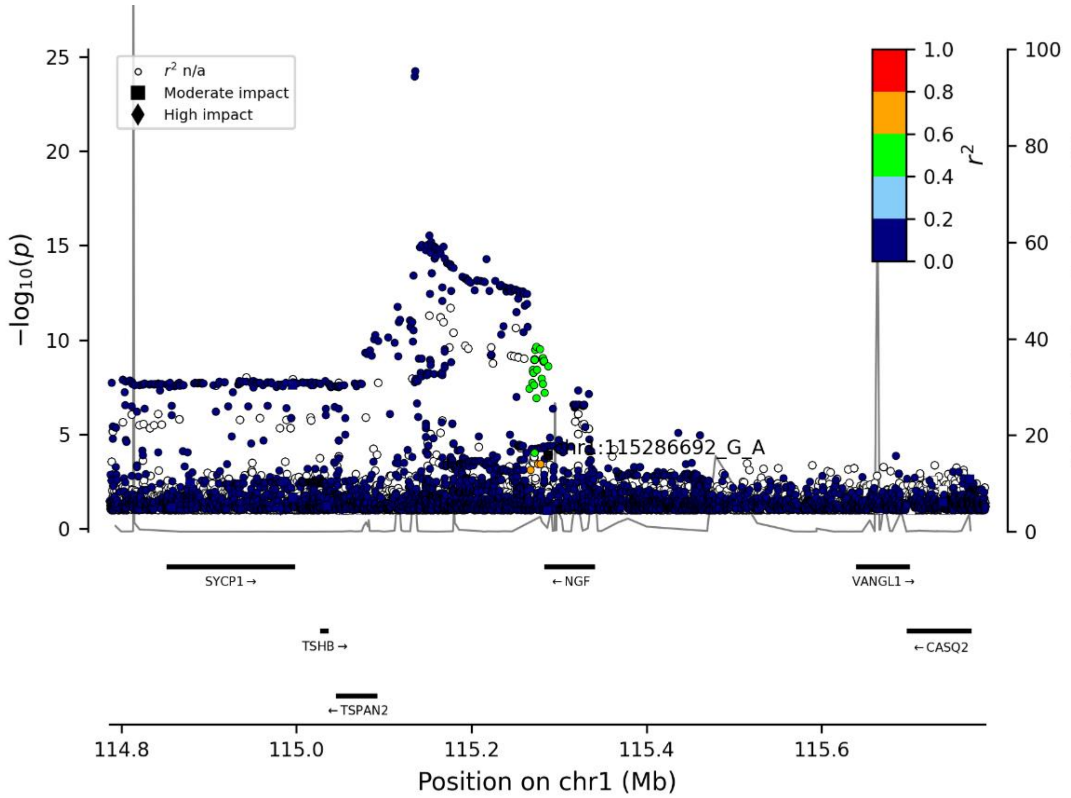

MIG: rs33985936 *SCN11A* missense variant (MAF = 25.0%))

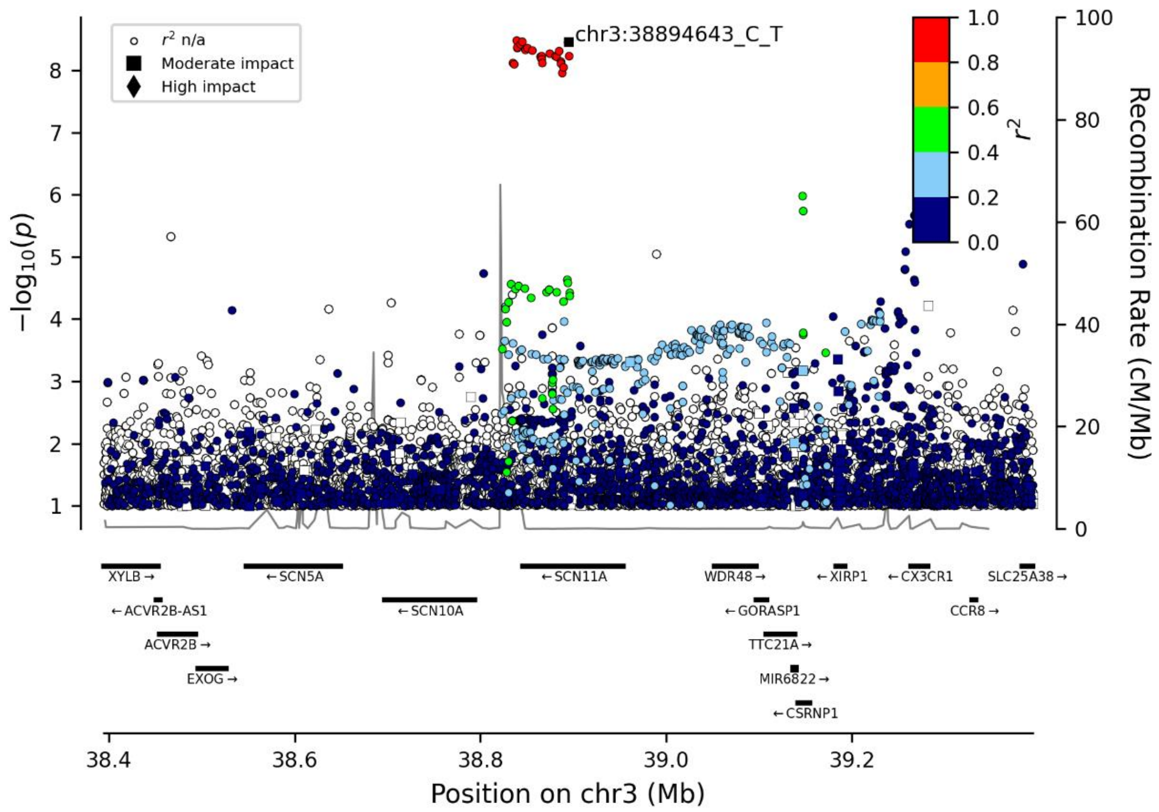

MIG: rs5753008 *HORMAD2* upstream variant (MAF = 35.6%)

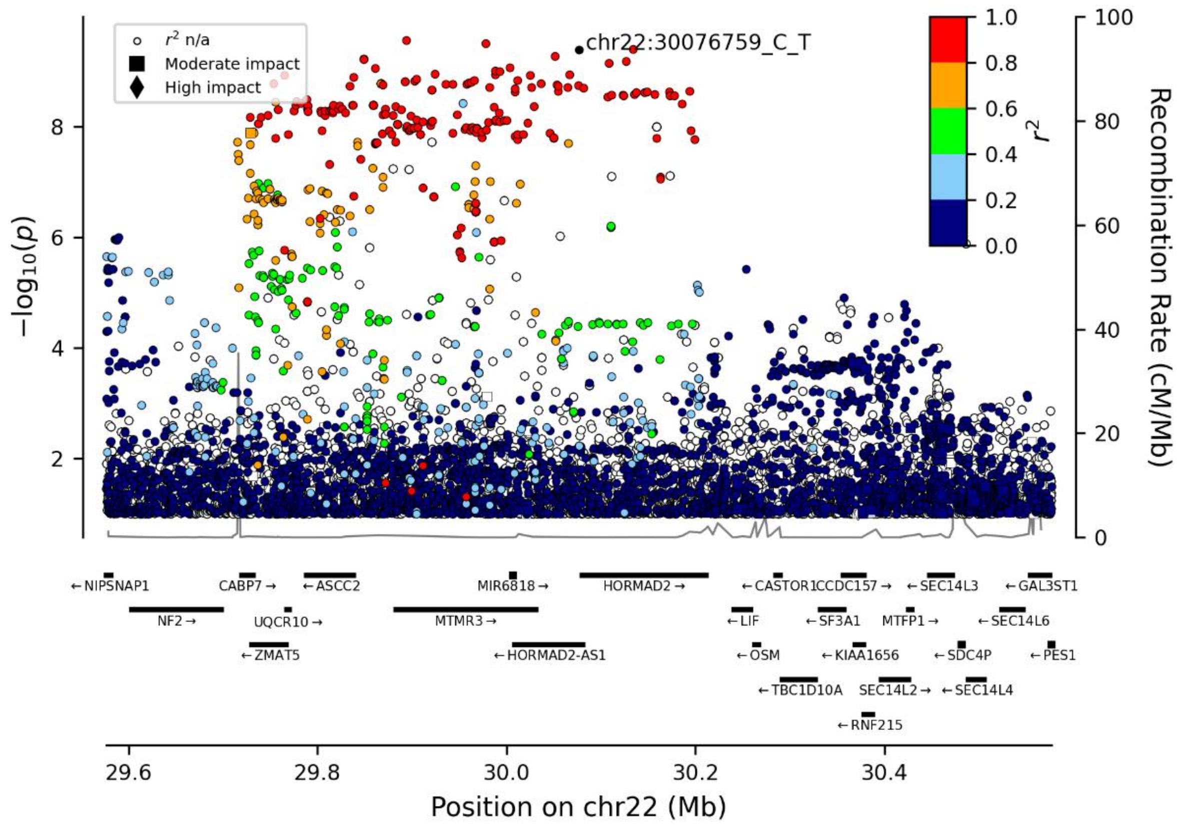

BRH: rs72854118 near *KCNK5* in cis-regulatory region (MAF = 0.67%)

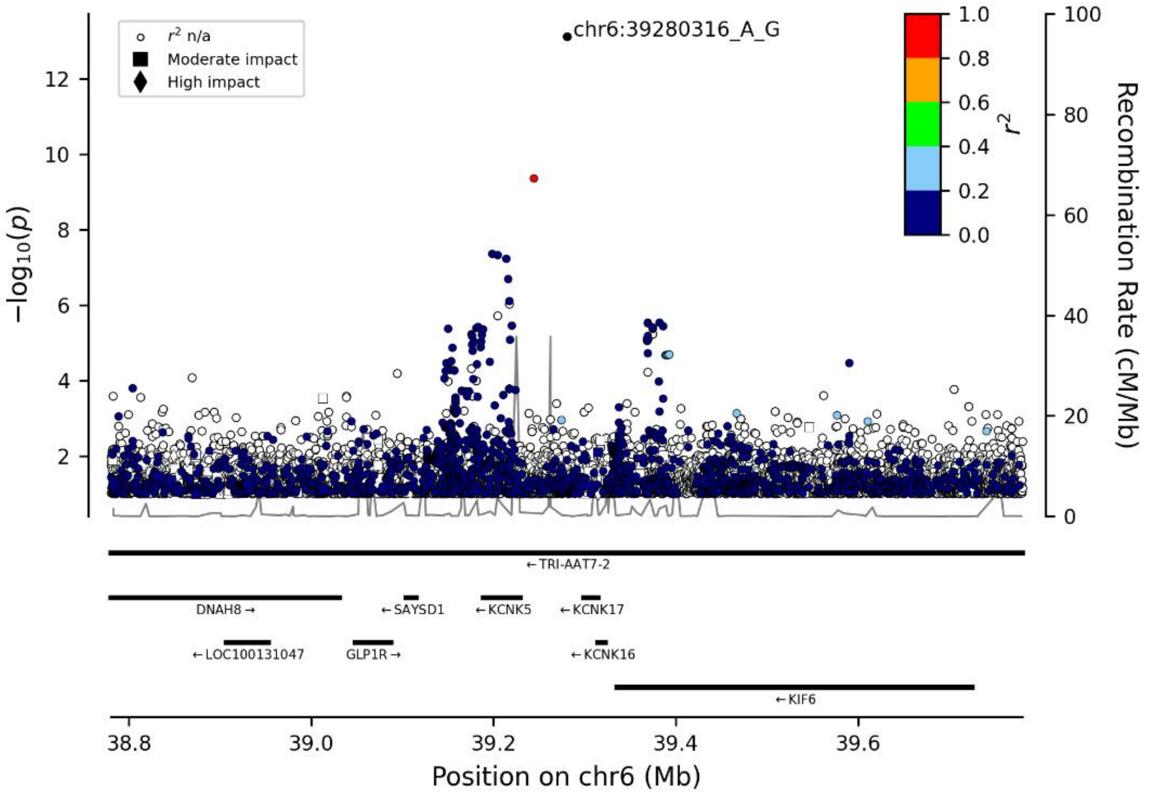

Figure 3 – Phenotypes studied and their genetic correlations

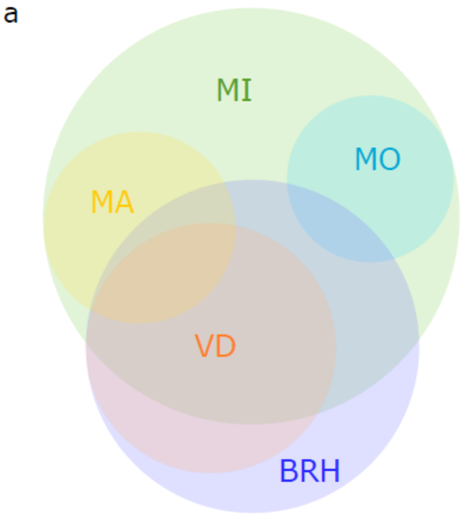

Meta-GWAS	cases	ctrls
Migraine	79,495	1,259,808
MO	11,718	1,330,747
MA	16,603	1,336,517
VD	30,297	86,134
BRH	51,803	123,732

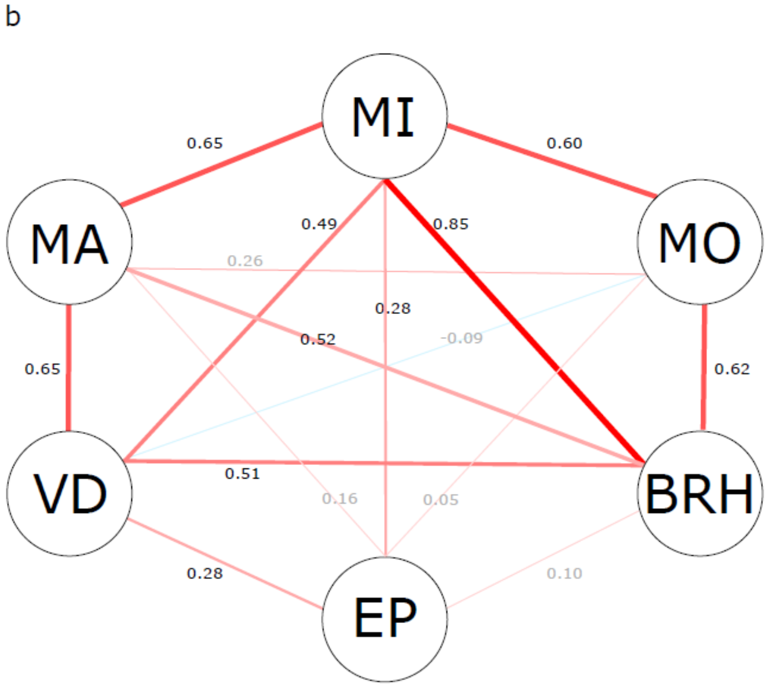

3a. Diagram of phenotypes studied and their relationships

Size of each phenotype circle represents total case numbers (shown in table, see sample sizes per cohort in Supplementary Table1). Clinically defined migraine (MI) includes migraine with aura (MA) and migraine without aura (MO). Self-reported Bad and recurrent headaches (BRH) are used as a proxy for severe migraine, and visual disturbances (VD) preceding such headaches, as a proxy for the aura experienced in migraine with aura (MA).

3b. Genetic correlations

Diagram shows genetic correlations between discovery phenotypes in current study in addition to Epilepsy (EP) (4 cohorts, $N_{case/ctrl}$ = 22,402/1,043,357), in light of the strong MA and Epilepsy association of the *PRRT2* variants. Using cross-trait LD score regression we calculated genetic correlations in non-overlapping samples (Methods). The significance level for the correlation estimates was determined using a simple Bonferroni correction for the number of meta-analyzed correlations. Significant ($P < 0.05/15 = 0.003$) correlation coefficients (r_g) are shown numerically in black and depicted by thickness of connecting lines, red are positive correlations, blue negative. Non-significant correlations are blurred (See Supplementary Table 8 for correlations with exact *P*-values).

Figure 4 – Lead variants stratified by VD vs MA

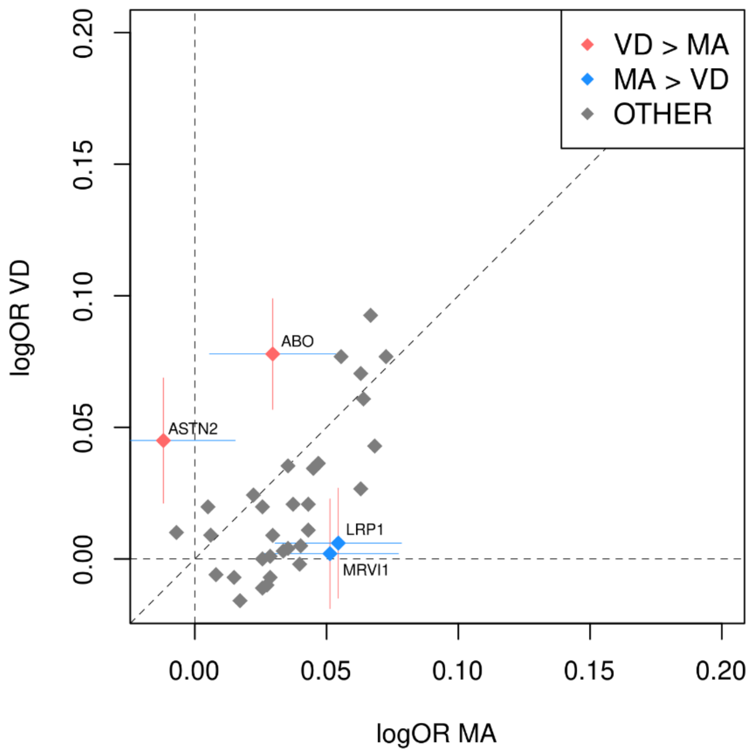

VD vs MA classification of lead variants (see Figure 3 in main text for plots depicting MA vs MO and VD vs MO). Effect plots for all lead variants (diamonds) excluding the MA variant in *PRRT2*. Data are presented as additive effect estimates (center) with 95% confidence intervals (CI) (crosses). Axes show logarithm of odds ratios (logOR) for VD (x-axis; $N_{case/control}$ = 30,297 / 86,134) and MA (y-axis; $N_{case/control}$ = 16,603 /1,336,517). LogOR is calculated for the effect allele. The effects of variants that have been colored and annotated with gene name differ between the migraine subtypes at a significance threshold of 0.0012 = 0.05/43. The 95% CIs for the logORs are shown for annotated variants. Effects are adjusted with sample overlap (r_{ij}) estimated from counts of cases, controls and the counts of overlaps in these groups between phenotypes[2] from all cohorts except FinnGen (for which we only have summary statistics). The parameter representing sample overlap between MA and VD r_{ij} = 0.283. Dashed lines show the coordinate axes, the diagonal, and a line through the origin with slope = 1. (Methods, Supplementary Tables 13-14)

Supplementary note on subtype classification analysis: We found that using the suggested[3] empirical Pearson correlation of effect sizes between migraine subtype GWAS statistics for estimation of the bivariate likelihood approximation (representing sample overlap) was in our data quite high or r_{ij} = 0.59 (Pearson correlation of betas using 7,858,264 markers in the summary statistics that have Effect Allele Frequency (EAF) > 5% and $P > 1 \times 10^{-4}$ for both traits). The r_{ij} for MO and VD was 0.103 (using 7,831,533 markers in the summary statistics with EAF > 5% and $P > 1 \times 10^{-4}$ for both traits). Hence, we evaluated sample overlap r_{ij} by using case and control counts[2] from all cohorts except FinnGen (as we only have summary statistics for this cohort) (Methods and Supplementary Tables 13-14 for analysis results). Using this method, the parameter representing sample overlap between MO and MA is r_{ij} = 0.023, MO and VD r_{ij} = 0.012, and between MA and VD r_{ij} = 0.283. Clearly, the correlation from the summary statistics reflects more the polygenic similarity of the migraine subtype traits rather than the sample overlap.

Figure 5 – *KCNK5*, BRH and brain aneurysms

5a. Brain aneurysm variants and BRH effects. Plot shows bad recurrent headache (BRH) effect versus brain aneurysm effect for 17 brain aneurysm variants[4] (black points), adding BRH variant rs72854118 from current study. Data are presented as additive effect estimates (center) with 95% confidence intervals (CI) (crosses). Axes show logarithm of odds ratios (logOR) for brain aneurysms from Bakker et al.[4] (x-axis; $N_{case/control}$ = 10,754 / 306,882) and BRH from current study (y-axis; $N_{case/control}$ = 51,803 /123,732).

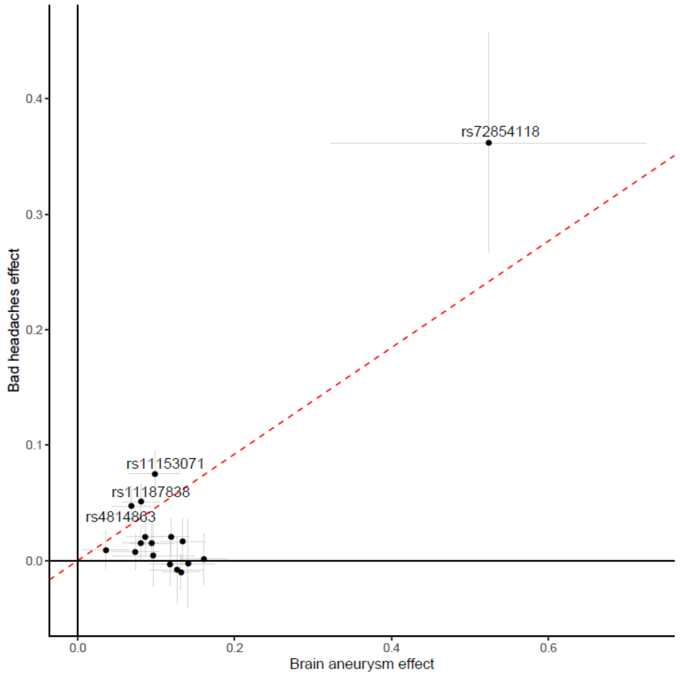

Brain aneurysm variants that are at known migraine loci and their BRH association in current study compared to rs72854118

rsname	Gene	EA	OR	*P*	r^2
rs72854118	*KCNK5*	G	0.697	7.57E-14	--
rs11153071	*FHL5*	A	1.078	4.49e-14	0.46
rs11187838	*PLCE1*	A	0.951	1.22e-10	1.00
rs4814863	*SLC24A3*	A	1.048	1.51e-07	1.00

r^2 is correlation with variant reported at locus in curent study

17 brain aneurysm variants from Bakker et al. 2020[4]:

Lead SNP	Locus	Annotated genes	Risk allele	Odds ratio	95% CI	Note
rs6841581	4q31.22	*EDNRA*	A	0.80	0.77–0.84	
rs4705938	5q31.1	*SLC22A5/ SLC22A4/P4HA2*	T	1.13	1.09–1.17	
**rs11153071**	**6q16.1**	***FHL5***	**A**	**1.16**	**1.11–1.22**	**Migraine Locus**
rs62516550	8q11.23	*SOX17*	T	1.17	1.12–1.22	
rs1537373	9p21.3	*CDKN2B-AS1*	T	0.84	0.81–0.86	
**rs11187838**	**10q23.33**	***PLCE1***	**A**	**0.92**	**0.89–0.94**	**Migraine Locus**
rs732998	10q24.33	*NT5C2/MARCKSL1P1*	T	1.19	1.14–1.25	
s2280543	11p15.5	*BET1L*	T	1.27	1.19–1.35	
rs11044991	12p12.2	*RP11-664H17.1*	A	0.88	0.84–0.92	
rs2681492	12q21.33	*ATP2B1*	T	1.12	1.08–1.17	
rs7137731	12q22	*FGD6/NR2C1*	T	0.89	0.86–0.92	
rs3742321	13q13.1	*STARD13*	T	0.87	0.84–0.90	
rs8034191	15q25.1	*PSMA4*	T	0.89	0.85–0.93	
rs7184525	16q23.1	*BCAR1/RP11-252K23.2*	A	1.15	1.11–1.19	
rs11661542	18q11.2	*RBBP8*	A	0.87	0.85–0.90	
**rs4814863**	**20p11.23**	***SLC24A3***	**A**	**1.11**	**1.07–1.15**	**Migraine Locus**
rs39713	22q12.2	*MTMR3*	T	1.20	1.12–1.28	

5b. Lead variants, BRH and brain aneurysms

Plot shows brain aneurysm effect versus bad recurrent headache (BRH) effect for all lead variants in current study reported in Tables 1 and 2 in manuscript (black points), excluding rare variant rs587778771 in *PRRT2* and rs933718575 near *A3GALT2* due to lack of carriers in brain aneurysms. Data are presented as additive effect estimates (center) with 95% confidence intervals (CI) (crosses). Axes show logarithm of odds ratios (logOR) for brain aneurysms from Bakker et al.[4] (x-axis; $N_{case/control}$ = 10,754 / 306,882) and BRH from current study (y-axis; $N_{case/control}$ = 51,803 /123,732).

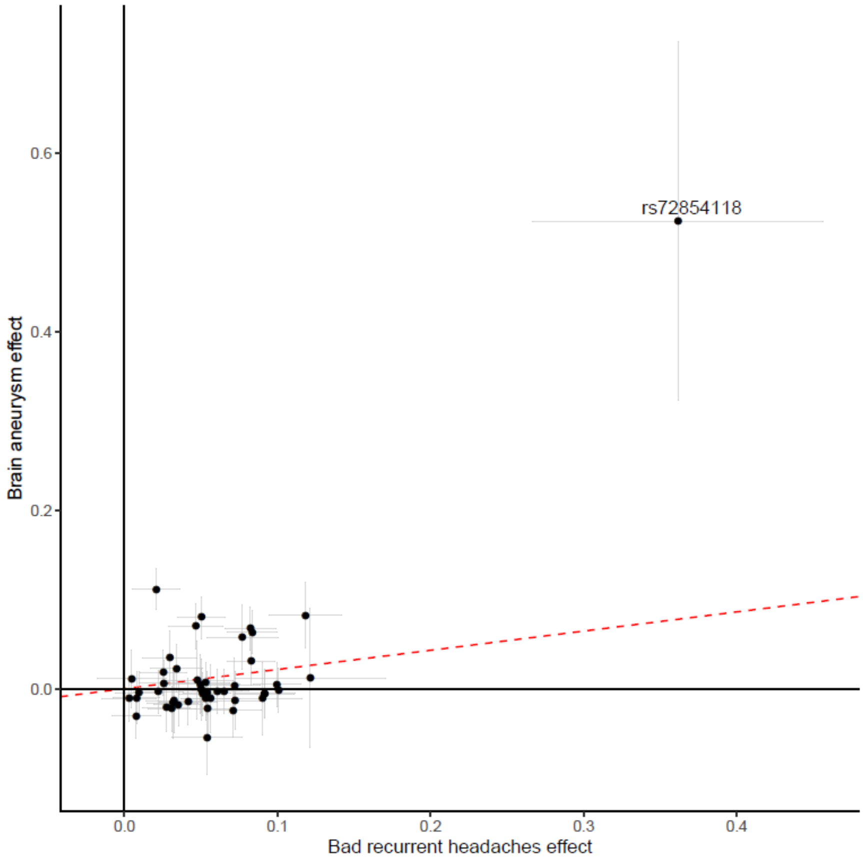

5c. Brain aneurysm effects vs migraine effects

Plot shows brain aneurysm effect vs migraine effect for all lead variants in current study reported in Tables 1 and 2 in manuscript (black points), excluding rare variant rs587778771 in *PRRT2* and rs933718575 near *A3GALT2* due to lack of carriers in brain aneurysms. Data are presented as additive effect estimates (center) with 95% confidence intervals (CI) (crosses). Axes show logarithm of odds ratios (logOR) for brain aneurysms from Bakker et al.[4] (x-axis; $N_{case/control}$ = 10,754 / 306,882) and Migraine from current study (y-axis; $N_{case/control}$ = 79,495 /1,259,808).

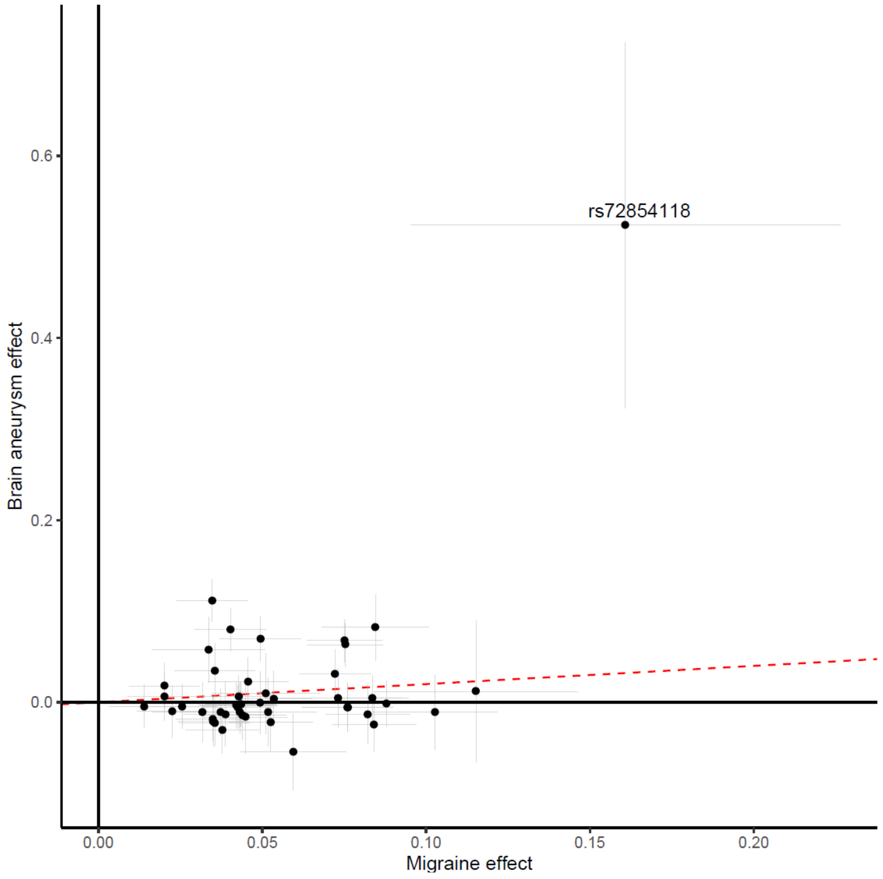

Figure 6 – Genes likely to mediate effects

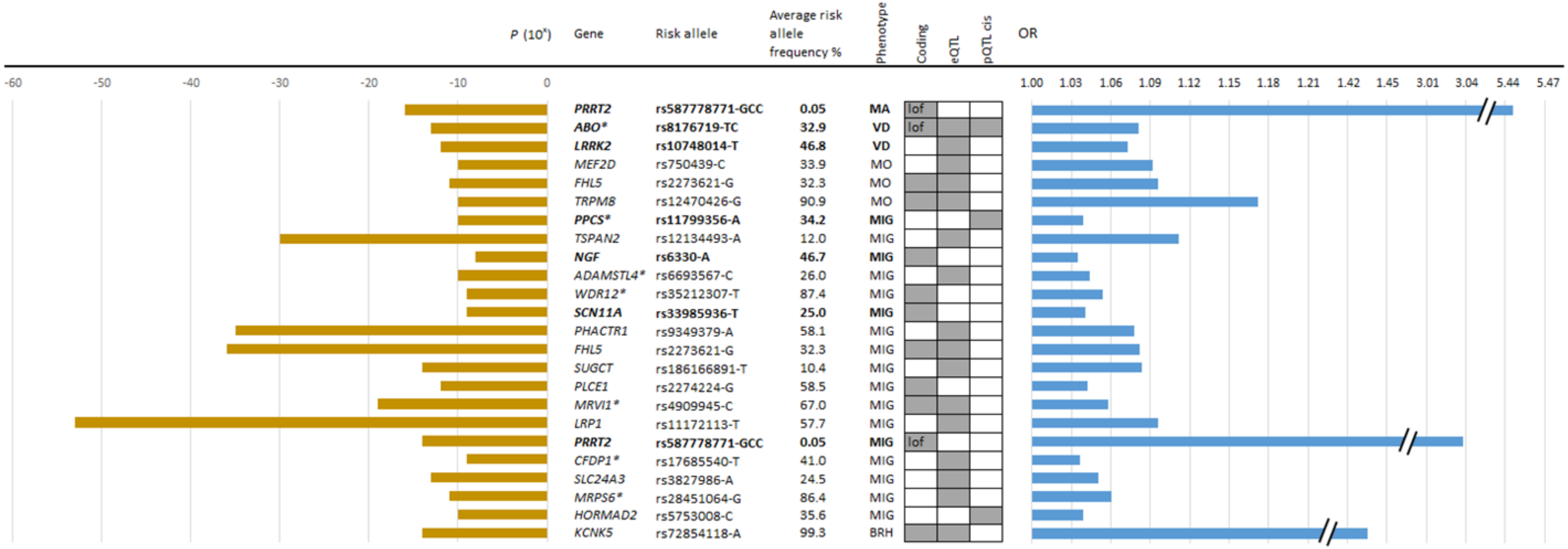

Sequence variants for which functional evidence implicates genes conferring risk of migraine (MIG), bad and recurrent headaches (BRH, the migraine-proxy), migraine with aura (MA), migraine without aura (MO), or visual disturbances (VD, proxy for MA). As depicted by gray boxes, the variants are either protein-coding variants (highest impact frameshift or loss-of-function marked lof), affect mRNA expression (top cis-eQTL), and/or associate with protein products of the respective genes as measured in plasma (pQTL)(Supplementary Tables 16-19). *Variants also associated in cis with mRNA or protein of other genes. The meta-analyses were performed using logistic regression, the risk (odds ratios (OR) in blue) are shown on the right scale for the risk-increasing allele, the OR bars truncated for the highest impact variants. Significance (*P*-value) is depicted on the left scale in yellow. In bold are variants that represent novel migraine-associated signals, others are previously reported (Tables 1 and 2). The *TRPM8* splice region variant rs2302153 is included here as it is in high LD with the lead variant reported at this locus.

Figure 7 – KCNK5 expression by rs72854118 in GTEx

Distribution of KCNK5 expression in tissues from GTEx (https://gtexportal.org/home/) stratified by rs72854118 genotype. The expression values are scaled in terms of transcripts per million (TPM) and log2-transformed. White boxes indicate quantile boundary (Q1-Q3) and the black horizontal line shows the median. Whiskers represent the ±1.5 times the interquartile range. The filled circles correspond to expression values, for non-carriers representing outliers that lie beyond the extremes of the whiskers. Although GTEx doesn´t test variants at frequencies <1%, we find three (n = 3) individuals that are rs72854118-G carriers, and one (n= 1) with KCNK5 expression data in kidney cortex that is higher than for non-carriers (n = 72) and one (n=1) with expression data in minor salivary gland, also higher than non-carriers (n=143).

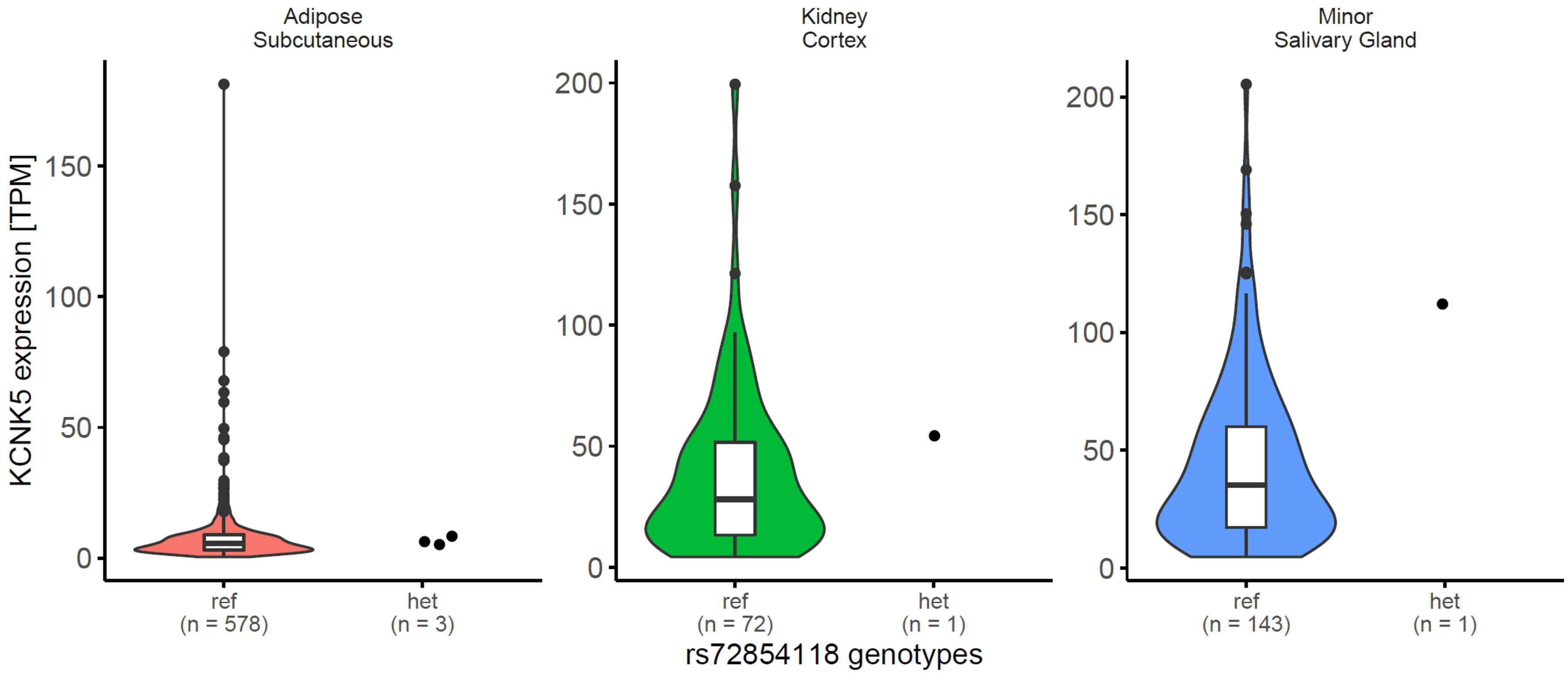

Supplementary Notes

1 – 123 migraine variants reported by Hautakangas et al.

The largest meta-GWAS on migraine to date by Hautakangas et al., included 102,084 cases and 771,257 controls and identified 123 migraine loci[3], while our meta-GWAS including 79,495 cases and 1,330,303 controls, identifies fewer migraine loci than expected, given an expected linear relationship between sample size and number of loci identified[5]. There are three plausible explanations for this:

First, despite the large number of controls our sample size for the clinically defined migraine analysis is actually smaller than in Hautakangas et al. We estimated the effective sample size for each GWAS used in our meta-analysis as Ne = 2*Na*Nc/(Na+Nc) * 1/lg, where Na and Nc are the number of cases and controls, respectively, and lg is genomic inflation factor. Adding the effective samples size for the different studies we estimate the effective sample size used in our study as 132,078 cases and the same number of controls, wereas for Hautakangas et al., based on sample sizes given in table 1 of their paper[3], we get 172,476 cases vs same number of controls.

Second, we use a more stringent threshold for genome-wide significance than was used by Hautakangas et al.'s study, which included only common variants and used *P*-value < 5e-8. In our study we included also rare variants and used tiered thresholds corresponding to a Bonferroni adjustment for number of variants within annotation categories of variants tested according to methods described by Sveinbjornsson et al.[1]. In our meta-GWAS of migraine results, we looked up the 123 variants identified by Hautakangas et al. and find that only 12/122 (one variant was not found in our data) were not significantly associated with migraine in our data (*P*-value ≥ 0.05), of which three had a *P*-value < 0.05 in association with a migraine subtype (see Supplementary Table 9). All variant effects reported in Hautakangas et al. had consistent direction of effects in our data in association with migraine. So in all, we find support for all but 9 variants reported in Hautakangas et al.

Third, we observe when we compare the effect estimates for the variants reported in Hautakangas et al. to the effect estimates in our meta-analysis of clinically defined migraine, that our estimates are on average about 70% of the estimates in Hautakangas et al. (Slope estimate by IVW log(OR) = 0.713, *P* = 1.16e-69, see figure below).

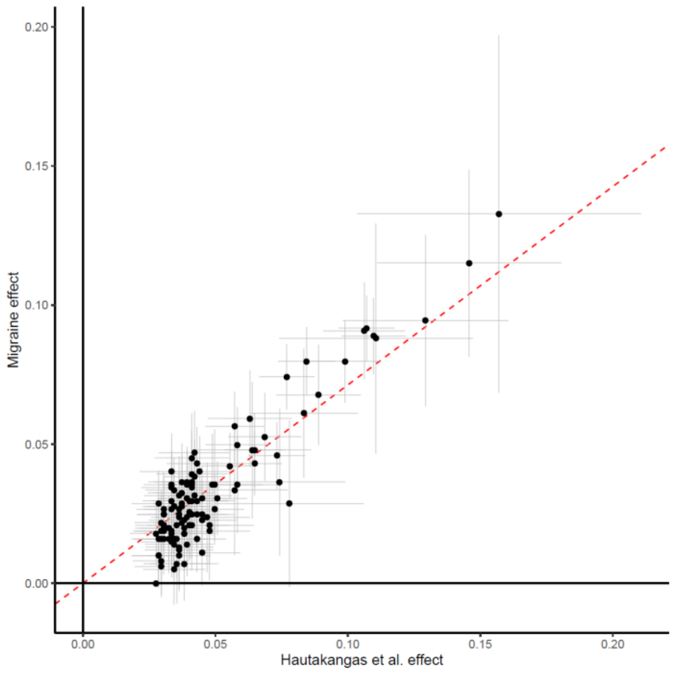

The plot on the left shows migraine effects of 122 out of the 123 migraine variants published by Hautakangas et al. (X-axis), against migraine effects in the current study (Y-axis). Effect estimates (black points) are expressed as logarithms of odds ratios (log(OR)) and grey crosses indicate 95% confidence intervals (CI) around effects. The dashed red line represents the linear regression fit through the origin, weighting variants according to the square of the standard error of their effect estimates (also known as inverse-variance weighted, IVW).

In the Hautakangas et al. study, cases and controls in the largest cohorts studied were defined from self-reported migraine, with controls reporting not having migraine[3]. In our study of clinically defined migraine, we used population controls.

Given estimated high rates of undiagnosed migraine in populations[6], our controls are likely to include more undiagnosed migraine than the controls in the Hautakangas et al. study. Thus, lower effect estimates in our meta-GWAS of migraine are to be expected and contribute to the lower number of associated variants in our study compared to Hautakangas et al..

2 – Results of *SCN11A* LOF burden test

Results of burden test combining effects of predicted *SCN11A* LOF variants in the UK Biobank data (combined frequency = 0.13%) on migraine and severe headaches (see Table 3 in manuscript and Supplementary Table 24 lists the 127 *SCN11A* LOF variants tested)

**UK phenotype (ICD diagnosis or UK Biobank field ID)**	**OR**	***P*-value**	**N case/ctrl**
Migraine (ICD10 G43)	0.650	3.90E-07	22,082/408,965
Headaches or migraine (f6159, SR, ICD10 G43, incl. triptan use)	0.663	8.10E-07	92,817/338,230
Headaches, all (f6159, SR)	*0.667*	*2.00E-06*	*87,391/343,656*
Bad, recurring headaches at any time in life (f120053, SR)	*0.604*	*2.70E-05*	*48,816/98,250*
Pain, pounding, pulsating, throbbing, when headaches were at worst (f120059, SR)	*0.307*	*3.70E-05*	*26,612/6,213*

Threshold of $P = 0.05/20{,}000$ genes[7] tested or $P = 2.5 \times 10^{-6}$. Effects in standard deviations (OR). For self-reports (SR) cases vs controls represents yes vs no. In italics are associations not reaching *P*-value threshold but included for effect comparisons.

3– Bad recurrent headache (BRH) variant and *KCNK5*

Bad recurrent headache (BRH) variant in regulatory region targeting *KCNK5* increases KCNK5 expression and protects against BRH and migraine:

In association with the meta-GWAS of a migraine by proxy phenotype (self-reported bad and recurrent headaches, based on GWAS results from three of the studied cohorts: UKB, Denmark and Iceland), we detect 14 signals. All are variants previously reported to associate with migraine (See Supplementary Tables 2-7). The lead variant for this signal, rs72854118 (chr6:39280316, hg38) is strongly correlated to two other sequence variants, rs72854120 (r^2=1.00) and rs72851880 (r^2=.88).

Functional annotation

We downloaded the cell type agnostic definition of candidate cis-regulatory elements (cCRE) from the ENCODE project[8] (screen.encodeproject.org) and tissue specific regulatory elements from Meuleman et al.[9] (zenodo.org/record/3838751#.YYUyjhrP2Ul). We then determined whether the lead sequence variant or any of their correlated variants (r^2 > 0.80) are located within cCRE or tissue specific regulatory regions. We looked for association signals in enhancer elements defined in EpiMap[8] (compbio.mit.edu/epimap) to then see if those same enhancers are predicted to influence nearby genes based on per-sample analysis datasets: personal.broadinstitute.org/cboix/epimap/links/links_corr_only.

The lead association variant (rs72854118), but not the other two correlated variants, is found within an enhancer-like sequence (dELS) as defined by ENCODE´s catalog of candidate cis-regulatory elements (cCRE)[8], which suggests non-coding regulatory functions for this signal (Table A below). This regulatory element is not found across tissue types, but is preferrentially found in renal tissues, cancers, and in embryonic tissues (Table B below).

Table: ***A)*** The lead variant of the GWAS association signal at chromosome 6q21.2 is located within a distal enhancer-like sequence defined in ENCODEs catalog of candidate cis-regulatory elements (cCREs) which is ***B)*** preferrentially found in open chromatin configuration in renal tissues and cancers.

A)

cCRE type	**GWAS association signal** *chr6p21.2*
CTCF-only-CTCF-bound	
dELS-CTCF-bound	
dELS	chr6:39280316:SG
DNase-H3K4me3-CTCF-bound	
DNase-H3K4me3	
pELS-CTCF-bound	
pELS	
PLS-CTCF-bound	
PLS	

B)

Tissue specificity	**GWAS association signal** *chr6p21.2*
Cancer-epithelial	
Cardiac	
Digestive	
Lymphoid	
Musculoskeletal	chr6:39244135:SG
Myeloid-erythroid	
Neural	
Organdevel-renal	
Placental-trophoblast	
Primitive-embryonic	chr6:39280316:SG
Pulmonarydevel	
Renal-cancer	chr6:39280316:SG
StromalA	
StromalB	
Tissue-invariant	
Vascular-endothelial	

The gene target for this regulatory element is *KCNK5* according to Epimap[10], a resource based on available data from ENCODE and Roadmap Epigenomics projects (Supplementary Table 21 – Predicted Gene Targets). Interestingly, the lead variant is located in binding sites for transcription factors in relevant cell types, *e.g.* VDR (vitamin D receptor), ETS1 (ETS proto-oncogene 1, transcription factor) and ZNF467 (zinc finger protein 467), in cells / cell-lines derived from kidney tissue (Supplementary Table 22 – DNA binding proteins).

The allele conferring the large protective effects on bad and recurrent headaches, rs72854118-G (OR = 0.70, *P* = 7.6E-14) is a VDR bindin motif – see figure C below. In Figure C, the G allele is depicted, enlarged proportionally with its importance for VDR binding and marked with black arrow.

Figure C – VDR binding motif at rs72854118

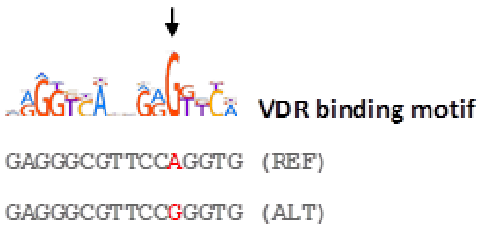

A common (MAF = 28.1%) variant, rs10456100-C in *KCNK5* was previously reported to associate with migraine (OR = 1.05, *P* = 9.2E-19) in the most recent meta-analysis by Hautakangas et al.[3] (Supplementary Table 9). The rare variant reported here, rs72854118, does not correlate with this common *KCNK5* variant (r^2 = 0.002).

The variant was not tested in GTEx, due to low frequency (MAF < 1%), however we found 3 carriers of rs72854118-G in GTEx data, one with expression data in kidney tissue where it is higher than in non-carriers and higher than in adipose and salivary gland tissue (See Supplementary Figure 3).

4 - FUMA Gene2Function analysis

Genes with evidence of functional effect on migraine phenotypes (https://fuma.ctglab.nl/).

ENSG	entrezID	symbol	OMIM	UniProtID	DrugBank	GeneCards
ENSG00000072952	10335	MRVI1	604673	Q9Y6F6	NA	GeneCard
ENSG00000112137	221692	PHACTR1	608723	Q9C0D0	NA	GeneCard
ENSG00000112214	9457	FHL5	605126	Q5TD97	NA	GeneCard
ENSG00000116604	4209	MEF2D	600663	Q14814	NA	GeneCard
ENSG00000123384	4035	LRP1	107770	Q07954	DB00025:DB00031:DB00100:DB06245:DB13152:DB13998:DB13999	GeneCard
ENSG00000127125	79717	PPCS	609853	Q9HAB8	NA	GeneCard
ENSG00000134198	10100	TSPAN2	613133	O60636	NA	GeneCard
ENSG00000134259	4803	NGF	162030	P01138	DB01407:DB05892:DB09221	GeneCard
ENSG00000138193	51196	PLCE1	608414	Q9P212	NA	GeneCard
ENSG00000138442	55759	WDR12	616620	Q9GZL7	NA	GeneCard
ENSG00000144481	79054	TRPM8	606678	Q7Z2W7	DB00825:DB01744:DB09061:DB11345:DB11755:DB14009:DB14011	GeneCard
ENSG00000153774	10428	CFDP1	608108	Q9UEE9	NA	GeneCard
ENSG00000164626	8645	KCNK5	603493	O95279	NA	GeneCard
ENSG00000167371	112476	PRRT2	614386	Q7Z6L0	NA	GeneCard
ENSG00000168356	11280	SCN11A	604385	Q9UI33	DB00907:DB00909	GeneCard
ENSG00000175164	28	ABO	110300	NA	NA	GeneCard
ENSG00000175600	79783	SUGCT	609187	Q9HAC7	NA	GeneCard
ENSG00000176635	150280	HORMAD2	NA	Q8N7B1	NA	GeneCard
ENSG00000185052	57419	SLC24A3	609839	Q9HC58	NA	GeneCard
ENSG00000188906	120892	LRRK2	609007	Q5S007	DB12010	GeneCard
ENSG00000243927	64968	MRPS6	611973	P82932	NA	GeneCard

Five of these genes are drug targets (Open Targets, https://platform.opentargets.org/):

- *LRP1* (a Prolow-density lipoprotein receptor-related protein 1 binding agent to treat various brain tumors),
- *NGF* (5 drugs, beta-nerve growth factor inhibitors (antibodies) to treat various forms of chronic pain, back pain, osteoarthritis, diabetic neuropathy etc),
- *TRPM8 (*3 drugs with 40 indications, including migraine and headaches)
- *SCN11A* (63 drugs (small molecules for unspecific blockage of several sodium channel alpha subunit blockers) to treat pain, epilepsy, cerebral palsy, seizures and atrial fibrillation
- *LRRK2* (inhibitor for treatment of Parkinson Disease).

a. Gene expression heatmap

GTEx v8 54 tissue types (Generated by FUMA https://fuma.ctglab.nl/). Input genes were the 22 genes for which variants with functional associations pointed to the gene associating with migraine or subtypes (Supplementary Figure 6). Expression value - average expression per label (log2 transformed), see Supplementary Table 19.

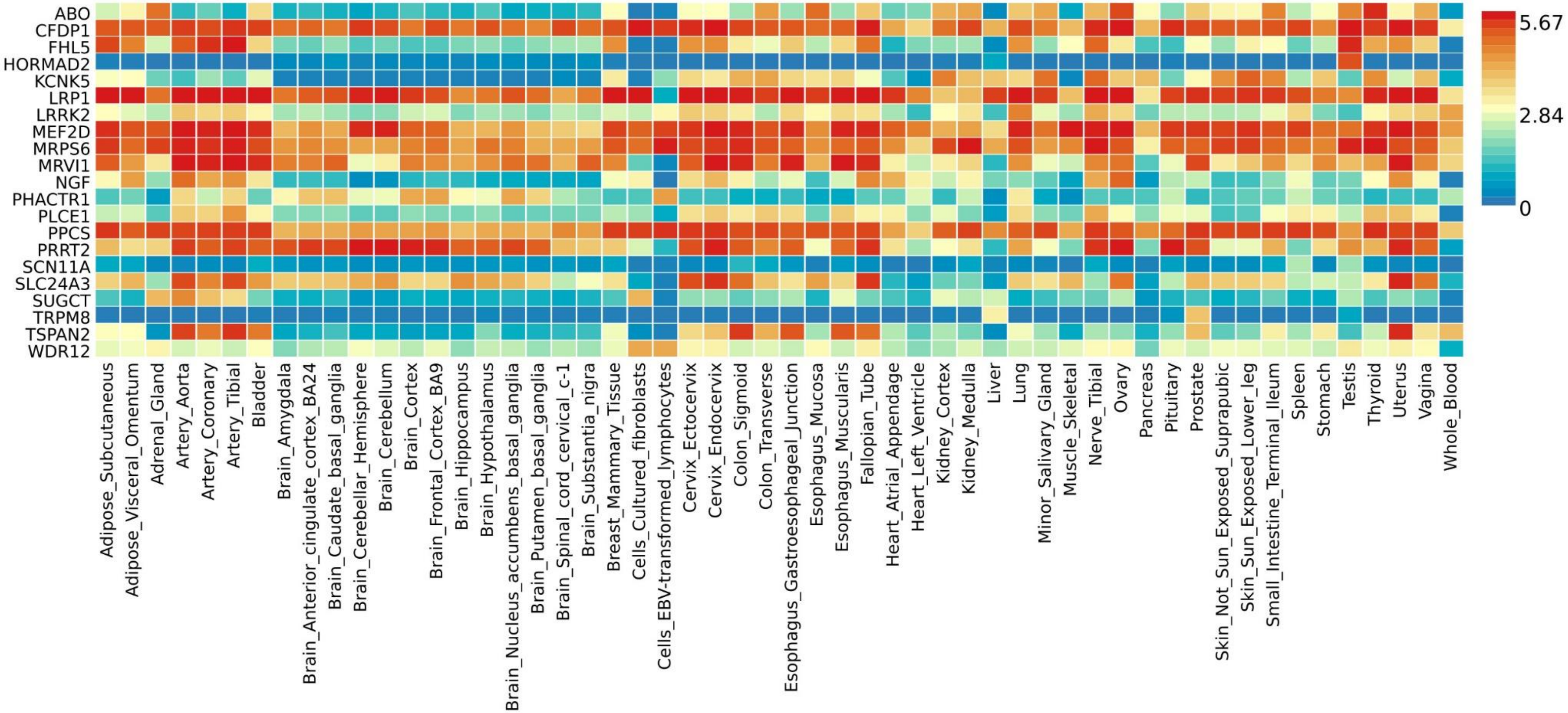

b. GWAS catalogue reported genes

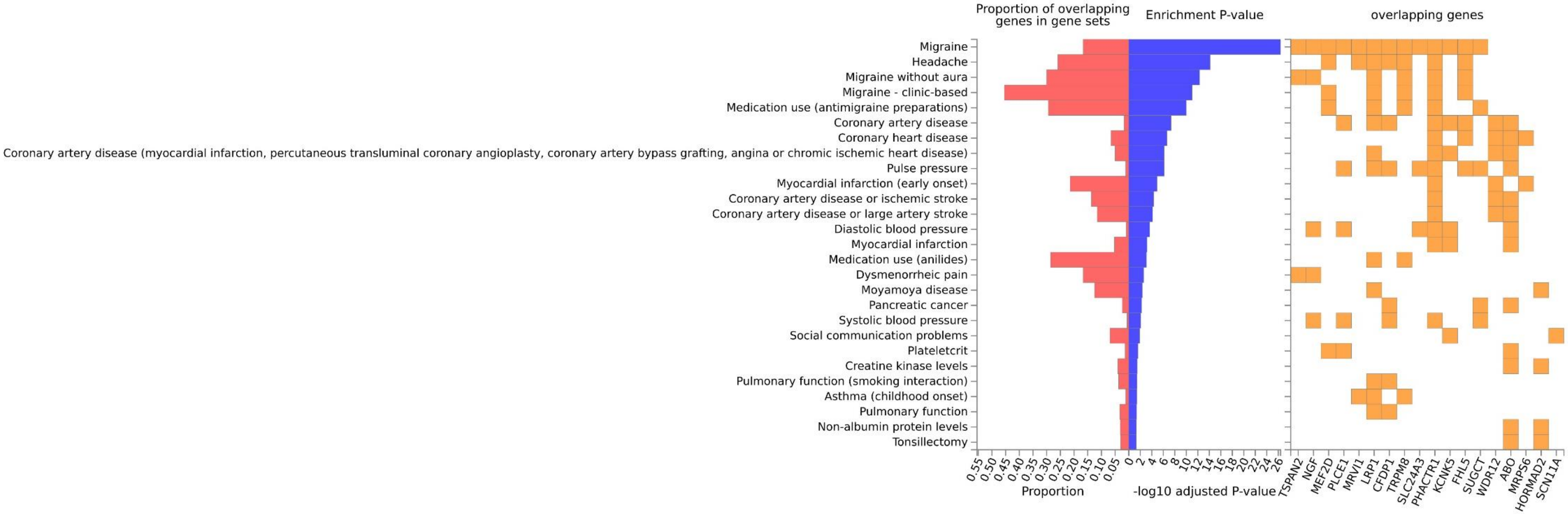

c. Differentially expressed genes (DEG)

Significantly enriched DEG sets ($P_{bon} < 0.05$) are highlighted in red (GTEx v8 54 tissue types)

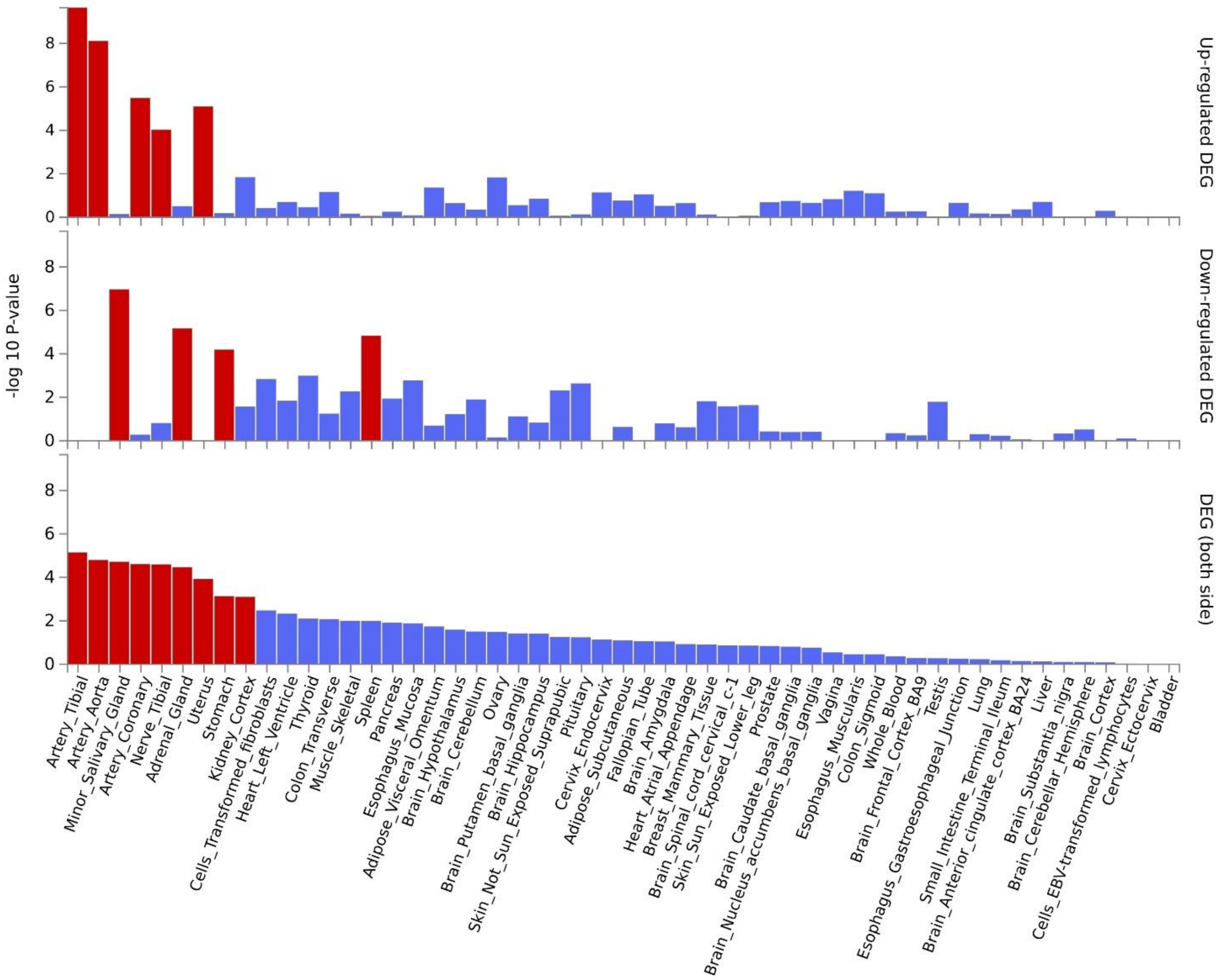

d. Brainspan – 29 different ages of brain samples

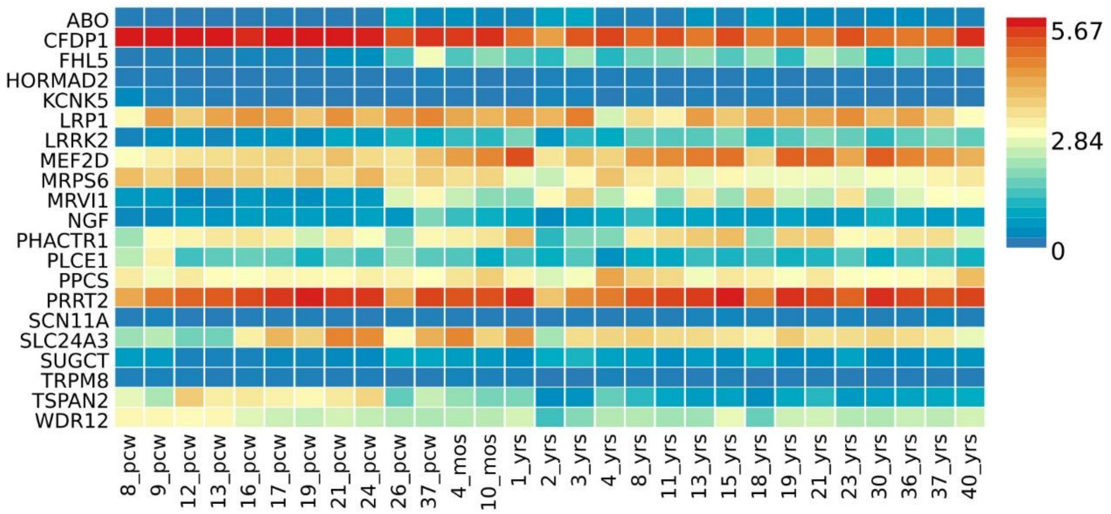

e. Brainspan – 11 general developmental stages of brain samples

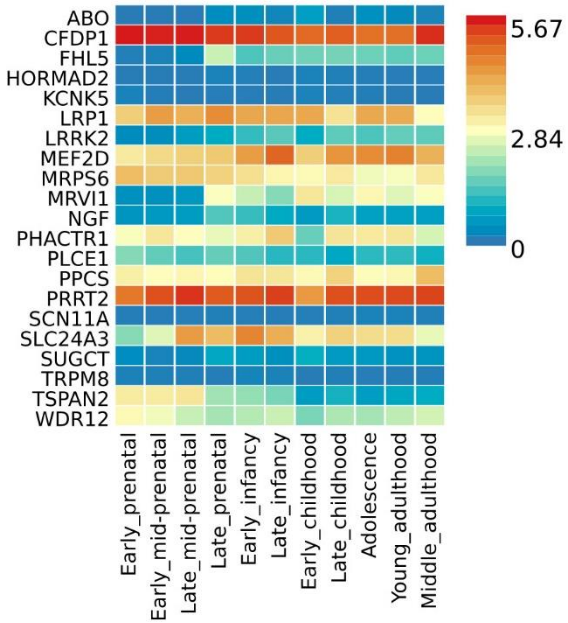

f. Brainspan – 11 general developmental stages of brain samples

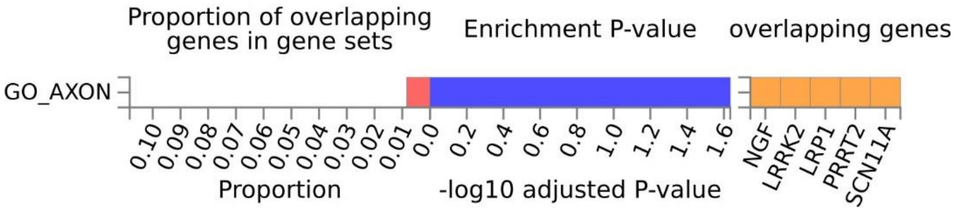

GO cellular components (MsigDB c5) (1)

Plot / Table

GeneSet	N	n	P-value	adjusted P	genes
GO_AXON	600	5	2.32e-5	2.32e-2	NGF, LRRK2, LRP1, PRRT2, SCN11A

5 - Genetic drug target analysis

Summary for the 22 genes for which we have functional evidence (Figure 6 in main text, in addition to the MA gene *CACNA1A*) we ran queries in The Drug-Interaction Database (https://www.dgidb.org/), the Illuminating the Druggable Genome (IDG) project (www.pharos.nih.gov), and Open Targets (www.opentargets.gov). Supplementary Table 23 lists all results of these queries. Additional sources for the information provided in the table below are from www.clinicaltrials.gov, and www.go.drugbank.com.

The IDG resource provides drug target information in three classes based on the degree to which the targets are studied:

- Tchem (Target has at least one ChEMBL compound): 4 genes: *KCNK5, LRRK2, NGF, CACNA1A*
- Tclin (Target with at least one approved drug): 2 genes: *SCN11A, TRPM8*
- Tbio (Targets that do not have known drug or small molecule activities): 17 genes: *ABO, ADAMTSL4, CFDP1, FHL5, HORMAD2, LRP1, MEF2D, MRPS6, MRVII1, PHACTR1, PLCE1, PPCS, PRRT2, SLC24A3, SUGCT, TSPAN2, WDR12*

**rsID lead SNP**	**Discovery phenotype**	**OR GWAS**	**PVAL GWAS (10E-X)**	**Gene**	**IDG family**	**Drug (name, group, indications, mode of action)**
rs8176719-TC	VD	1.081	13	*ABO*	Enzyme	None in DGIDB, IDG, OpenTargets, or ClinicalTrials.gov.<br>go.drugbank.com lists 13 experimental drugs targeting ABO – unknown indications
rs6693567-C	MIG	1.044	10	*ADAMSTL4**	Other	None
rs11085837-A	VD	0.926	14	*CACNA1A*	Ion	Mibefradil: a benzimidazoyl-substituted tetraline that selectively binds and inhibits CALCIUM CHANNELS, T-TYPE, but is a nonselective calcium channel blocker developed for treatment of hypertension and chronic angina (https://pharos.nih.gov)<br>Roche pulled it from market after FDA approval due to due to the potential for lethal drug interactions. Several other non-selective calcium channel blockers have been developed see Supplementary Table 23.

rsID lead SNP	Discovery phenotype	OR GWAS	PVAL GWAS (10E-X)	Gene	IDG family	Drug (name, group, indications, mode of action)
rs17685540-T	MIG	1.037	9	*CFDP1**	Other	None
rs2273621-G	MO	1.096	11	*FHL5*	Other	None
rs2273621-G	MIG	1.082	36	*FHL5*	Other	None
rs5753008-C	MIG	1.039	10	*HORMAD2*	Other	None
rs72854118-A	MIG	1.435	14	*KCNK5*	Ion channel	Halothane: positive modulator, a nonflammable, halogenated, hydrocarbon anesthetic that provides relatively rapid induction with little or no excitement. Analgesia may not be adequate. NITROUS OXIDE is often given concomitantly. Because halothane may not produce sufficient muscle relaxation, supplemental neuromuscular blocking agents may be required. (From AMA Drug Evaluations Annual, 1994, p178 in IDG)
rs11172113-T	MIG	1.096	53	*LRP1*	Other	ANG1005: is a Prolow-density lipoprotein receptor-Related protein 1 binding agent that is in phase II trials against brain cancer, glioma/glioblastoma. Other targets of ANG1005 are various microtubule genes: e.g. TUBA4A, TUBB3, TUBB2B. Has not been tested in migraine. (From OpenTargets)
rs10748014-T	VD	1.073	12	*LRRK2*	Kinase	Sunitinib: An indole and pyrrole derivative that inhibits VEGFR-2 and PDGFR BETA RECEPTOR TYROSINE KINASES. It is used as an antineoplastic agent for the treatment of GASTROINTESTINAL STROMAL TUMORS, and for treatment of advanced or metastatic RENAL CELL CARCINOMA.<br>Fostamatinib: is a tyrosine kinase inhibitor with demonstrated activity against spleen tyrosine kinase (SYK). The major metabolite of fostamatinib, R406, inhibits signal transduction of Fc-activating receptors and B-cell receptor. The fostamatinib metabolite R406 reduces antibody-mediated destruction of platelets. It is used for treatment of chronic immune thrombocytopenia.<br>DGIDB lists 24 other ligands targeting *LRRK2* (Supplementary Table 23).<br>ClinicalTrials.gov lists 28 trials of drugs targeting *LRRK2* for Parkinson´s Disease

rsID<br>lead SNP	Discovery phenotype	OR GWAS	PVAL GWAS (10E-X)	Gene	IDG family	Drug (name, group, indications, mode of action)
rs750439-C	MO	1.092	10	*MEF2D*	Transcription factor	None
rs28451064-G	MIG	1.061	11	*MRPS6**	Other	None
rs4909945-C	MIG	1.058	19	*MRVI1**	Other	None
rs6330-A	MIG	1.035	8	*NGF*	Other	Fasinumab, Tanezumab, Fulranumab:<br>Beta-nerve growth fafctor inhibitors (antibodies) tested for osteoarthritis paink, low back pain, abdominal pain, sciatica and other pain conditions.<br>ClinicalTrials.gov lists 215 trials of drugs targeting NGF for various indications, mainly pain.<br>These inhibitors have not been studied in migraine.
rs9349379-A	MIG	1.078	35	*PHACTR1*	Enzyme	Zibotentan:<br>rs9349379 regulates expression of Endothelin-1, (encoded by upstream gene *EDN1*) in human vascular cells. ClinicalTrials.gov lists 29 studies of Zibotentan, a potent selective inhibitor of the ETA receptor, to treat microvascular angina. The minor allele rs9349379-A, that confers protective effects on migraine, enhances circulating endothelin-1, a potent vasoconstrictor. Originally developed by AstraZeneca for cancer treatment, prior research has confirmed that Zibotentan (a vasodilator)relaxes small blood vessels of patients with MVA. Has not been studied in migraine.
rs2274224-G	MIG	1.043	12	*PLCE1*	Enzyme	Hydrochlorothiazide:<br>A thiazide diuretic to lower blood pressure. Variants in PLCE1 found to associate with hydro-Chlorothiazide response (PMID: 31327267) . Can benefit to reduce edema in menstrual migraine.
rs11799356-A	MIG	1.039	10	*PPCS**	Enzyme	None
rs587778771-GCC	MA	5.446	16	*PRRT2*	Other	None in IDG, OpenTargets, DrugBank or ClinicalTrials.gov.<br>Bryostatin:<br>A potent modulator of protein kinase C (PKC), that is in several trials to improve cognitive function in Alzheimer´s disease according to ClinicalTrials.gov. Pre-clinical studes indicate that PKC is a critical mechanism for generation and maintenance of migraine.<br>Bryostatin has not been tested in migraine. In DGIDB (no PMID or source reference).

rsID lead SNP	Discovery phenotype	OR GWAS	PVAL GWAS (10E-X)	Gene	IDG family	Drug (name, group, indications, mode of action)
rs587778771-GCC	MIG	3.038	16	*PRRT2*	Other	See above
rs33985936-T	MIG	1.041	9	*SCN11A*	Ion Channel	All sources list several approved drugs (targeting voltage-gated sodium channel blockers in general, are not specific to Nav1.9 encoded by SCN11A. Several have been tested to treat migraine, including Lidocaine, Ropivacaine (blockers), Topiramate (antagonist). Carbamazepine also non-selectively targets voltage-gated sodium channels, is an anticonvulsant and with several other indications including neuropathic pain (Supplementary Table 23)
rs3827986-A	MIG	1.050	13	*SLC24A3*	Transporter	None
rs186166891-T	MIG	1.084	14	*SUGCT*	Enzyme	None
rs12470426-G	MO	1.172	10	*TRPM8*	Ion Channel	Levomenthol: A monoterpene cyclohexanol produced from mint oils, is an approved drug that has mild cooling and decongestant properties that help soothe cough and ease congestion in the lungs. Used also topically for headache relief (applied to forehead). Several active ligands for *TRPM8* are listed in DGIDB and IGD
rs12134493-A	MIG	1.112	30	*TSPAN2*	Other	None
rs35212307-T	MIG	1.054	9	*WDR12**	Other	None

References

1. Sveinbjornsson, G. *et al.* Weighting sequence variants based on their annotation increases power of whole-genome association studies. *Nat Genet* **48**, 314-7 (2016).
2. Bhattacharjee, S. *et al.* A Subset-Based Approach Improves Power and Interpretation for the Combined Analysis of Genetic Association Studies of Heterogeneous Traits. *The American Journal of Human Genetics* **90**, 821-835 (2012).
3. Hautakangas, H. *et al.* Genome-wide analysis of 102,084 migraine cases identifies 123 risk loci and subtype-specific risk alleles. *Nat Genet* **54**, 152-160 (2022).
4. Bakker, M.K. *et al.* Genome-wide association study of intracranial aneurysms identifies 17 risk loci and genetic overlap with clinical risk factors. *Nat Genet* **52**, 1303-1313 (2020).
5. Visscher, P.M. *et al.* 10 Years of GWAS Discovery: Biology, Function, and Translation. *Am J Hum Genet* **101**, 5-22 (2017).
6. Katsarava, Z., Mania, M., Lampl, C., Herberhold, J. & Steiner, T.J. Poor medical care for people with migraine in Europe – evidence from the Eurolight study. *The Journal of Headache and Pain* **19**, 10 (2018).
7. Lonsdale, J. *et al.* The Genotype-Tissue Expression (GTEx) project. *Nature Genetics* **45**, 580-585 (2013).
8. Consortium, E.P. *et al.* Expanded encyclopaedias of DNA elements in the human and mouse genomes. *Nature* **583**, 699-710 (2020).
9. Meuleman, W. *et al.* Index and biological spectrum of human DNase I hypersensitive sites. *Nature* **584**, 244-251 (2020).
10. Boix, C.A., James, B.T., Park, Y.P., Meuleman, W. & Kellis, M. Regulatory genomic circuitry of human disease loci by integrative epigenomics. *Nature* **590**, 300-307 (2021).
