# Supplementary material for: Rare variants with large effects provide functional insights into the pathology of migraine subtypes, with and without aura
Source: Nat Genet. 2023 Oct 26;55(11):1843–53. doi: 10.1038/s41588-023-01538-0 (PMC10632135; doi:10.1038/s41588-023-01538-0)
Supplement: Supplementary file 2 — Reporting Summary [file 41588_2023_1538_MOESM2_ESM.pdf]

nature portfolio

Corresponding author(s): Gyda Bjornsdottir

Last updated by author(s): Apr 13, 2022

Reporting Summary

Nature Portfolio wishes to improve the reproducibility of the work that we publish. This form provides structure for consistency and transparency in reporting. For further information on Nature Portfolio policies, see our Editorial Policies and the Editorial Policy Checklist.

Statistics

For all statistical analyses, confirm that the following items are present in the figure legend, table legend, main text, or Methods section.

n/a	Confirmed	
☐	☒	The exact sample size (*n*) for each experimental group/condition, given as a discrete number and unit of measurement
☐	☒	A statement on whether measurements were taken from distinct samples or whether the same sample was measured repeatedly
☐	☒	The statistical test(s) used AND whether they are one- or two-sided<br>*Only common tests should be described solely by name; describe more complex techniques in the Methods section.*
☐	☒	A description of all covariates tested
☐	☒	A description of any assumptions or corrections, such as tests of normality and adjustment for multiple comparisons
☐	☒	A full description of the statistical parameters including central tendency (e.g. means) or other basic estimates (e.g. regression coefficient) AND variation (e.g. standard deviation) or associated estimates of uncertainty (e.g. confidence intervals)
☐	☒	For null hypothesis testing, the test statistic (e.g. *F*, *t*, *r*) with confidence intervals, effect sizes, degrees of freedom and *P* value noted<br>*Give P values as exact values whenever suitable.*
☒	☐	For Bayesian analysis, information on the choice of priors and Markov chain Monte Carlo settings
☒	☐	For hierarchical and complex designs, identification of the appropriate level for tests and full reporting of outcomes
☐	☒	Estimates of effect sizes (e.g. Cohen's *d*, Pearson's *r*), indicating how they were calculated

Our web collection on statistics for biologists contains articles on many of the points above.

Software and code

Policy information about availability of computer code

Data collection	No software was used for data collection

Data analysis

In conjunction with methods developed at deCODE Genetics as described in the methods section, we used publicly available software that is available on request under the following URLs:
GraphTyper (v2.0-beta, GNU GPLv3 license): https://github.com/DecodeGenetics/graphtyper,
Eagle (version 2.4.1): http://www.hsph.harvard.edu/alkes-price/software/,
Shapeit 4: https://odelaneau.github.io/shapeit4/
ADMIXTURE (v1.23):https://dalexander.github.io/admixture/
BOLT-LMM (v.2.1): http://www.hsph.harvard.edu/alkes-price/software/,
R (version 3.6.3): https://www.r-project.org/,
R package ggplot for visualization (version 3.3.3): https://ggplot2.tidyverse.org/,
Ensembl v.87: https://www.ensembl.org/index.html,
IMPUTE2 v.2.3.1: https://mathgen.stats.ox.ac.uk/impute/impute_v2.html,
dbSNP v.140: http://www.ncbi.nlm.nih.gov/SNP/,
kallisto v.0.46: https://github.com/pachterlab/kallisto,
For subtype stratification analysis we used R code available at: https://github.com/mjpirinen/migraine-meta,
MAGMA (v1.08): http://ctglab.nl/software/magma,
Variant Effect Predictor (release 100): https://github.com/Ensembl/ensembl-vep and FUMA:at https://fuma.ctglab.nl/.
Sequencher 5.0: https://sequencher.software.informer.com/5.0/
No custom code was written for this study.

For manuscripts utilizing custom algorithms or software that are central to the research but not yet described in published literature, software must be made available to editors and reviewers. We strongly encourage code deposition in a community repository (e.g. GitHub). See the Nature Portfolio guidelines for submitting code & software for further information.

Data

Policy information about availability of data

All manuscripts must include a data availability statement. This statement should provide the following information, where applicable:

- Accession codes, unique identifiers, or web links for publicly available datasets
- A description of any restrictions on data availability
- For clinical datasets or third party data, please ensure that the statement adheres to our policy

Our previously described Icelandic population WGS data have been deposited at the European Variant Archive under accession PRJEB15197 (https://www.ebi.ac.uk/ena/browser/view/PRJEB15197). The GWAS summary statistics for the migraine GWAS meta-analyses are available at https:// www.decode.com/summarydata/. FinnGen data are publicly available and were downloaded from https://www.finngen.fi/en/access_results. The UKB data were downloaded under application no. 42256. Proteomics data and protein mapping to UniProt identifiers and gene names were provided by SomaLogic and Olink. Other data generated or analyzed in this study are included in the article and its Supplementary tables and information.

Human research participants

Policy information about studies involving human research participants and Sex and Gender in Research.

Reporting on sex and gender

No sex-specific results are reported.

Population characteristics

Our study is based on data from study participants of European descent from 6 populations (Iceland, Denmark, Norway, UK, USA, Finnland). A description of all population characteristics is included in the methods section. Genetic ancestry filtering and principal components determining European ancestry in each population are also described in methods. Cases with migraine and the migraine subtypes with and without aura, were in all cohorts but Norway (using self-reported migraine from questionnaires), mainly defined by International Classification of Diseases (ICD-10) codes (or comparable codes from earlier versions of ICD) representing migraine with aura (MA, code G43.1, migraine without aura (MO, G43.0) and overall migraine (G43). Diagnostic codes were assigned by physicians and captured through both inpatient and outpatient diagnostic registries. As triptan medications (Anatomical Therapeutic Chemical (ATC) code N02CC) are used to prevent/treat migraine attacks, subjects who had received triptan subscriptions were identified in data from drug registries (Iceland, Denmark, Finland and UK) and added to migraine cases (without subtype). For the MA-proxy phenotype used in this study (Visual disturbances preceding headaches, VD), we defined cases and controls from questionnaire data obtained in the studies conducted in Iceland, Denmark and the UK Biobank. Questions used in Icelandic and Danish cohorts were comparable to the question answered by participants in the UK Biobank (Data-Field 120065: Data description: Visual changes before or near the onset of headaches, Question: "I develop visual changes such as spots, lines and heat waves or greying out of my vision". Responses "Yes" were compared to responses "No". Such defined cases with, and controls without, headache-related visual disturbances had all previously responded "Yes" to a question on headaches as asked in the UK Biobank survey (Data-Field 120053: Data description: Bad and/or recurring headaches at any time in life, Question: "Have you ever had bad and/or recurring headaches at any time in your life?"). We used this UK Biobank data field 120053 as a migraine-proxy, defining comparable severity qualified headache questions in Icelandic and Danish questionnaire datasets for the GWAS meta-analysis.

Recruitment

The GWAS meta-analyses reported in the study were performed using data from 6 populations (Iceland, Denmark, Norway, UK, USA, Finland) and results across populations were compared. Recruitment and phenotype assessment information for each population is detailed in the methods section. In short:

Icelandic participants were recruited into the ongoing deCODE Genetics migraine studies and various other deCODE research projects. About 155K, or close to half of the Icelandic population of 340K has participated in various ongoing nationwide research programs at deCODE Genetics.

Danish samples and data were obtained in collaboration with the ongoing recruitment performed by the Copenhagen Hospital Biobank Study and the Danish Blood Donor Study (DBDS).
Data on Norwegian migraine cases and controls were obtained from the Hordaland Health study (HUSK), a population-based study recruiting participants in Hordaland county in Western Norway.
UK participants were recruited into the UK Biobank study that since 2006 has collected extensive phenotype and genotype data from about 500,000 participants, recruited widely from across the UK.
Participants from the US were recruited via ongoing studies at Intermountain Healthcare in Utah.
Data from Finnland was obtained from the ongoing FinnGen study, that consists of samples collected from the Finnish biobanks and phenotype data collected at Finland´s national health registers.

Ethics oversight

All data and samples on which this study is based, were collected under licenses obtained from the respective studies' local ethics and data privacy protection committees and under informed consent of participants, as described in detail in the methods section of the manuscript. In short:
Icelandic data were analyzed under a study on the genetics of migraine approved by the National Bioethics Committe (NBC#; 19-158-V3, VSNb2019090003/03.01) following review by the Icelandic Data Protection Authority (DPA).
Danish data were analyzed under the "Genetics of pain and degenerative diseases" protocol, approved by the Danish Data Protection Agency (P-2019-51) and the National Committee on Health Research Ethics (NVK-18038012). The Danish Data Protection Agency (P-2019-99) and the National Committee on Health Research Ethics (NVK-1700407) approved the studies under which data on DBDS participants were obtained for this study.
Data from the UK Biobank were analyzed under approval from The North West Research Ethics Committee, which reviewed and approved UK Biobank's scientific protocol and operational procedures (REC Reference Number: 06/MRE08/65). This study was conducted under the approved application number 42256.
The Finnish data were analyzed under approval from The Coordinating Ethics Committee of the Helsinki and Uusimaa Hospital District, which evaluated and approved the FinnGen research project. The FinnGen project complies with existing legislation (in particular the Biobank Law and the Personal Data Act). The official data controller of the FinnGen study is the University of Helsinki.
Analyses of data from the US (Intermountain) were approved by The Intermountain Healthcare Institutional Review Board and all participants provided written informed consent and samples for genotyping.
Norwegian data were analyzed under approval from the National Health Screening Service, Oslo (now the Norwegian Institute of Public Health) in cooperation with the University of Bergen.

Note that full information on the approval of the study protocol must also be provided in the manuscript.

Field-specific reporting

Please select the one below that is the best fit for your research. If you are not sure, read the appropriate sections before making your selection.

☒ Life sciences ☐ Behavioural & social sciences ☐ Ecological, evolutionary & environmental sciences

For a reference copy of the document with all sections, see nature.com/documents/nr-reporting-summary-flat.pdf

Life sciences study design

All studies must disclose on these points even when the disclosure is negative.

Sample size	Sample sizes are reported in the article and correspond to all available data.
Data exclusions	No available data was excluded from the study, other than data from participants of non-European ethnicity as described for all cohorts in methods.
Replication	The GWAS meta-analyses reported in the study were performed using data from 6 populations (Iceland, Denmark, Norway, UK, USA, Finnland) and association results across populations are provided in our results. We did not conduct replication since we had all study data available to us included in the GWAS meta-analysis. For all known migraine variants published by Hautakangas et al.,2022, we provide association results with all studied phenotypes.
Randomization	Randomization was not performed. Within each GWAS included in the GWAS meta-analyses, covariates were adjusted for to account for potential confounding. Covariate adjustments are reported in detail in methods.
Blinding	Group allocation was not relevant to this study, hence blinding was not performed.

Reporting for specific materials, systems and methods

We require information from authors about some types of materials, experimental systems and methods used in many studies. Here, indicate whether each material, system or method listed is relevant to your study. If you are not sure if a list item applies to your research, read the appropriate section before selecting a response.

Materials & experimental systems

n/a	Involved in the study
☒	☐ Antibodies
☒	☐ Eukaryotic cell lines
☒	☐ Palaeontology and archaeology
☒	☐ Animals and other organisms
☒	☐ Clinical data
☒	☐ Dual use research of concern

Methods

n/a	Involved in the study
☒	☐ ChIP-seq
☒	☐ Flow cytometry
☒	☐ MRI-based neuroimaging
