# Supplementary material for: Rare variants with large effects provide functional insights into the pathology of migraine subtypes, with and without aura
Source: Nat Genet. 2023 Oct 26;55(11):1843–53. doi: 10.1038/s41588-023-01538-0 (PMC10632135; doi:10.1038/s41588-023-01538-0)
Supplement: Supplementary file 4 — Reactome gene pathway report. [file 41588_2023_1538_MOESM4_ESM.pdf]

Pathway Analysis Report

This report contains the pathway analysis results for the submitted sample ''. Analysis was performed against Reactome version 81 on 30/07/2022. The web link to these results is:

https://reactome.org/PathwayBrowser/#/ANALYSIS=MjAyMjA3MzAxMzMzNDhfMjIxNTc%3D

Please keep in mind that analysis results are temporarily stored on our server. The storage period depends on usage of the service but is at least 7 days. As a result, please note that this URL is only valid for a limited time period and it might have expired.

Table of Contents

1. Introduction
2. Properties
3. Genome-wide overview
4. Most significant pathways
5. Pathways details
6. Identifiers found
7. Identifiers not found

1. Introduction

Reactome is a curated database of pathways and reactions in human biology. Reactions can be considered as pathway 'steps'. Reactome defines a 'reaction' as any event in biology that changes the state of a biological molecule. Binding, activation, translocation, degradation and classical biochemical events involving a catalyst are all reactions. Information in the database is authored by expert biologists, entered and maintained by Reactome’s team of curators and editorial staff. Reactome content frequently cross-references other resources e.g. NCBI, Ensembl, UniProt, KEGG (Gene and Compound), ChEBI, PubMed and GO. Orthologous reactions inferred from annotation for Homo sapiens are available for 17 non-human species including mouse, rat, chicken, puffer fish, worm, fly, yeast, rice, and Arabidopsis. Pathways are represented by simple diagrams following an SBGN-like format.

Reactome's annotated data describe reactions possible if all annotated proteins and small molecules were present and active simultaneously in a cell. By overlaying an experimental dataset on these annotations, a user can perform a pathway over-representation analysis. By overlaying quantitative expression data or time series, a user can visualize the extent of change in affected pathways and its progression. A binomial test is used to calculate the probability shown for each result, and the p-values are corrected for the multiple testing (Benjamini–Hochberg procedure) that arises from evaluating the submitted list of identifiers against every pathway.

To learn more about our Pathway Analysis, please have a look at our relevant publications:

Fabregat A, Sidiropoulos K, Garapati P, Gillespie M, Hausmann K, Haw R, … D’Eustachio P (2016). The reactome pathway knowledgebase. Nucleic Acids Research, 44(D1), D481–D487. https://doi.org/10.1093/nar/gkv1351.

Fabregat A, Sidiropoulos K, Viteri G, Forner O, Marin-Garcia P, Arnau V, … Hermjakob H (2017). Reactome pathway analysis: a high-performance in-memory approach. BMC Bioinformatics, 18.

2. Properties

- This is an **overrepresentation** analysis: A statistical (hypergeometric distribution) test that determines whether certain Reactome pathways are over-represented (enriched) in the submitted data. It answers the question 'Does my list contain more proteins for pathway X than would be expected by chance?' This test produces a probability score, which is corrected for false discovery rate using the Benjamani-Hochberg method.
- 16 out of 23 identifiers in the sample were found in Reactome, where 160 pathways were hit by at least one of them.
- All non-human identifiers have been converted to their human equivalent.
- This report is filtered to show only results for species 'Homo sapiens' and resource 'UniProt'.
- The unique ID for this analysis (token) is MjAyMjA3MzAxMzMzNDhfMjIxNTc%3D. This ID is valid for at least 7 days in Reactome's server. Use it to access Reactome services with your data.

3. Genome-wide overview

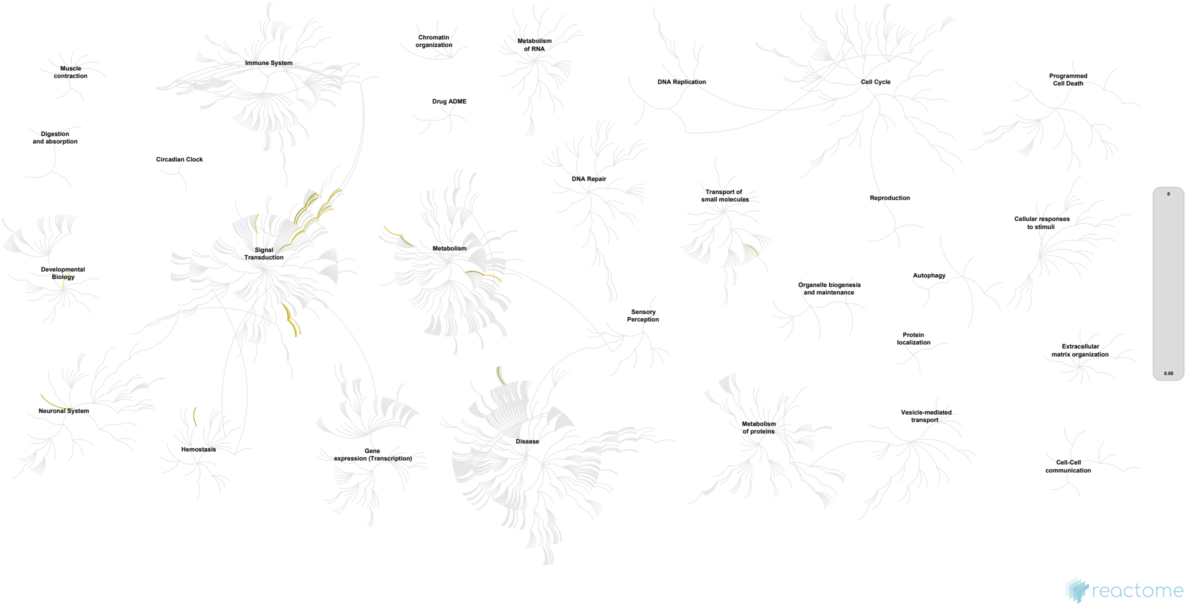

This figure shows a genome-wide overview of the results of your pathway analysis. Reactome pathways are arranged in a hierarchy. The center of each of the circular "bursts" is the root of one top-level pathway, for example "DNA Repair". Each step away from the center represents the next level lower in the pathway hierarchy. The color code denotes over-representation of that pathway in your input dataset. Light grey signifies pathways which are not significantly over-represented.

4. Most significant pathways

The following table shows the 25 most relevant pathways sorted by p-value.

Pathway name	Entities				Reactions	
	found	ratio	p-value	FDR*	found	ratio
Myogenesis	2 / 29	0.002	0.002	0.108	2 / 14	0.001
TRKA activation by NGF	1 / 3	2.58e-04	0.007	0.108	4 / 4	2.87e-04
PLC-gamma1 signalling	1 / 3	2.58e-04	0.007	0.108	3 / 3	2.15e-04
NFG and proNGF binds to p75NTR	1 / 3	2.58e-04	0.007	0.108	2 / 2	1.44e-04
Signalling to STAT3	1 / 3	2.58e-04	0.007	0.108	1 / 1	7.18e-05
ABO blood group biosynthesis	1 / 3	2.58e-04	0.007	0.108	4 / 7	5.02e-04
Ceramide signalling	1 / 3	2.58e-04	0.007	0.108	1 / 2	1.44e-04
Expression and Processing of Neurotrophins	1 / 4	3.44e-04	0.009	0.108	5 / 5	3.59e-04
NGF processing	1 / 4	3.44e-04	0.009	0.108	5 / 5	3.59e-04
Axonal growth stimulation	1 / 4	3.44e-04	0.009	0.108	1 / 2	1.44e-04
Signalling to p38 via RIT and RIN	1 / 5	4.31e-04	0.012	0.108	3 / 3	2.15e-04
NADE modulates death signalling	1 / 6	5.17e-04	0.014	0.108	3 / 3	2.15e-04
PTK6 promotes HIF1A stabilization	1 / 6	5.17e-04	0.014	0.108	4 / 6	4.31e-04
p75NTR negatively regulates cell cycle via SC1	1 / 6	5.17e-04	0.014	0.108	1 / 3	2.15e-04
ARMS-mediated activation	1 / 7	6.03e-04	0.016	0.108	6 / 6	4.31e-04
Activation of TRKA receptors	1 / 7	6.03e-04	0.016	0.108	4 / 8	5.74e-04
Coenzyme A biosynthesis	1 / 8	6.89e-04	0.018	0.108	1 / 7	5.02e-04
PI3K/AKT activation	1 / 9	7.75e-04	0.021	0.108	3 / 5	3.59e-04
p75NTR regulates axonogenesis	1 / 10	8.61e-04	0.023	0.108	1 / 8	5.74e-04
Sodium/Calcium exchangers	1 / 11	9.47e-04	0.025	0.108	1 / 4	2.87e-04
Presynaptic depolarization and calcium channel opening	1 / 12	0.001	0.028	0.108	1 / 1	7.18e-05
FGFR1c ligand binding and activation	1 / 12	0.001	0.028	0.108	2 / 3	2.15e-04
Signaling by activated point mutants of FGFR1	1 / 12	0.001	0.028	0.108	2 / 4	2.87e-04
Frs2-mediated activation	1 / 12	0.001	0.028	0.108	5 / 13	9.33e-04
NF-kB is activated and signals survival	1 / 13	0.001	0.03	0.108	2 / 4	2.87e-04

* False Discovery Rate

5. Pathways details

For every pathway of the most significant pathways, we present its diagram, as well as a short summary, its bibliography and the list of inputs found in it.

1. Myogenesis (R-HSA-525793)

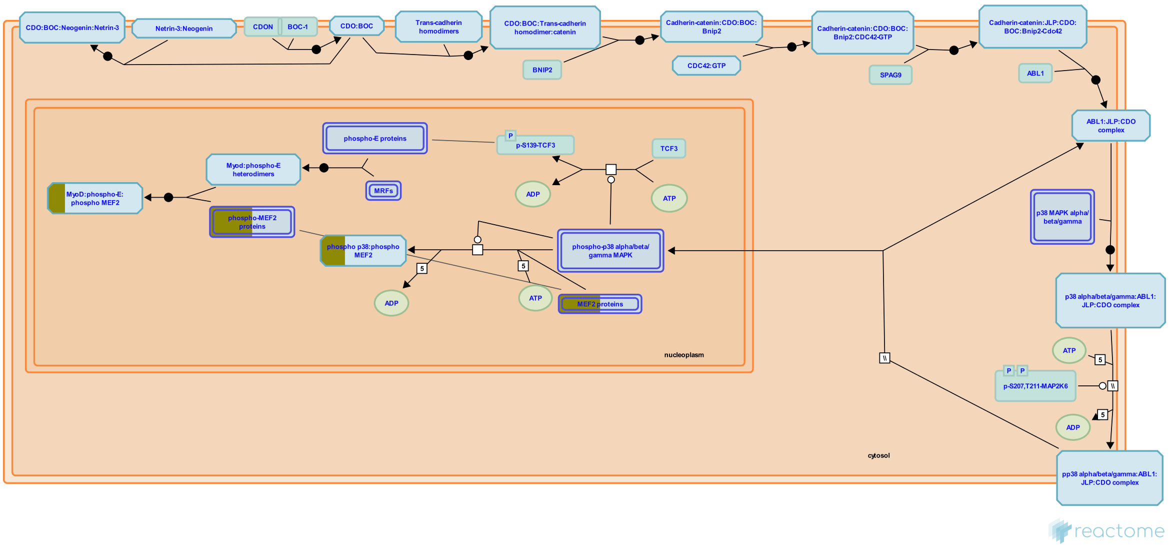

Myogenesis, the formation of muscle tissue, is a complex process involving steps of cell proliferation mediated by growth factor signaling, cell differentiation, reorganization of cells to form myotubes, and cell fusion. Here, one regulatory feature of this process has been annotated, the signaling cascade initiated by CDO (cell-adhesion-molecule-related/downregulated by oncogenes) and associated co-receptors.

CDO/Cdon is a type I transmembrane multifunctional co-receptor consisting of five immunoglobulin and three fibronectin type III (FNIII) repeats in the extracellular domain, and an intracellular domain with no identifiable motifs. It has been implicated in enhancing muscle differentiation in promyogenic cells. CDO exert its promyogenic effects as a component of multiprotein complexes that include the closely related factor Boc, the Ig superfamily receptor neogenin and its ligand netrin-3, and the adhesion molecules N- and M-cadherin. CDO modulates the Cdc42 and p38 mitogen-activated protein kinase (MAPK) pathways via a direct association with two scaffold-type proteins, JLP and Bnip-2, to regulate activities of myogenic bHLH factors and myogenic differentiation. CDO activates myogenic bHLH factors via enhanced heterodimer formation, most likely by inducing hyper-phosphorylation of E proteins.

Myogenic basic helix-loop-helix (bHLH) proteins are master regulatory proteins that activate the transcription of many muscle-specific genes during myogenesis. These myogenic bHLH proteins also referred to as MyoD family includes four members, MyoD, myogenin, myf5 and MRF4. These myogenic factors dimerize with E-proteins such as E12/E47, ITF-2 and HEB to form heterodimeric complexes that bind to a conserved DNA sequence known as the E box, which is present in the promoters and enhancers of most muscle-specific genes. Myocyte enhancer binding factor 2 (MEF2), which is a member of the MADS box family, also plays an important role in muscle differentiation. MEF2 activates transcription by binding to the consensus sequence, called the MEF2-binding site, which is also found in the control regions of numerous muscle-specific genes. MEF2 and myogenic bHLH proteins synergistically activate expression of muscle-specific genes via protein-protein interactions between DNA-binding domains of these heterologous classes of transcription factors. Members of the MyoD and MEF2 family of transcription factors associate combinatorially to control myoblast specification, differentiation and proliferation.

References

Kang JS, Cole F, Takaesu G, Gaio U, Zhang W & Krauss RS (2005). Close encounters: regulation of vertebrate skeletal myogenesis by cell-cell contact. J Cell Sci, 118, 2355-62.

Edit history

Date	Action	Author
2010-02-09	Reviewed	Krauss RS
2010-02-16	Edited	Garapati P V
2010-02-16	Authored	Garapati P V
2010-02-22	Created	Matthews L
2022-05-20	Modified	Weiser JD

1 submitted entities found in this pathway, mapping to 2 Reactome entities

Input	UniProt Id
MEF2D	Q02078, Q14814

2. TRKA activation by NGF (R-HSA-187042)

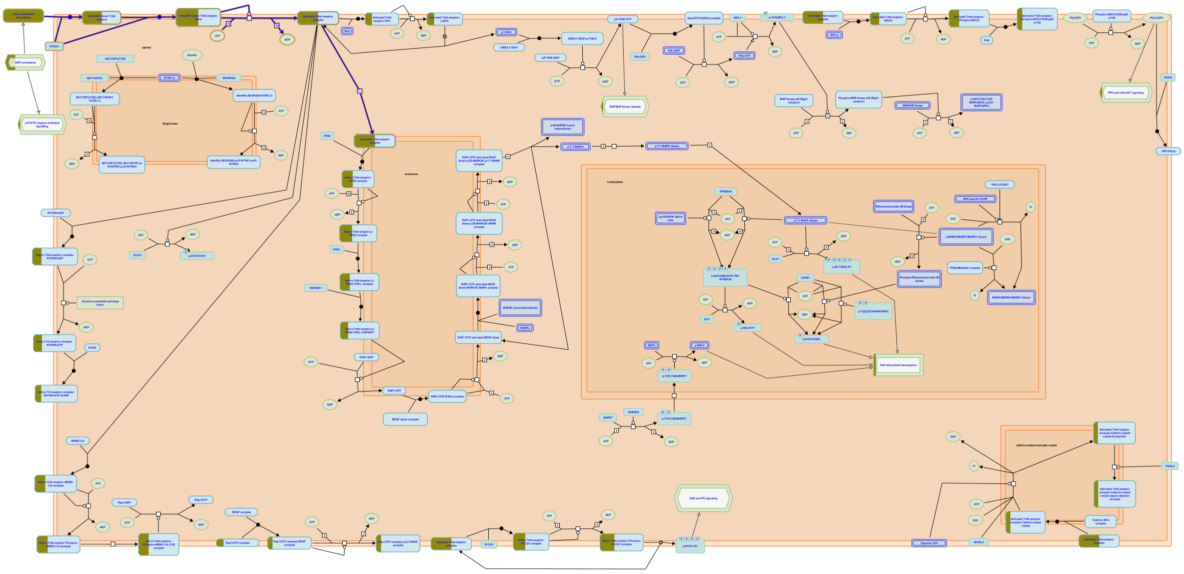

Neurotrophin functions are mediated by binding of the secreted neurotrophin homodimers to their common neurotrophin receptor p75NTR, and to their cognate tropomyosin related kinase (TRK) receptor. NGF binds to TRKA, BDNF and NT4 bind to TRKB, NT3 binds to TRKC. A tri-molecular signalling complex (NGF-p75NTR-TRKA) might also be possible.

References

Barker PA & Murphy RA (1992). The nerve growth factor receptor: a multicomponent system that mediates the actions of the neurotrophin family of proteins. Mol Cell Biochem, 110, 1-15.

Barbacid M, Lamballe F & Klein R (1991). The trk family of oncogenes and neurotrophin receptors. Princess Takamatsu Symp, 22, 153-70.

Edit history

Date	Action	Author
2006-09-07	Created	Jassal B
2006-10-10	Edited	Jassal B
2006-10-10	Authored	Annibali D, Nasi S
2007-11-08	Reviewed	Greene LA
2022-05-20	Modified	Weiser JD

1 submitted entities found in this pathway, mapping to 1 Reactome entities

Input	UniProt Id
NGF	P01138

3. PLC-gamma1 signalling (R-HSA-167021)

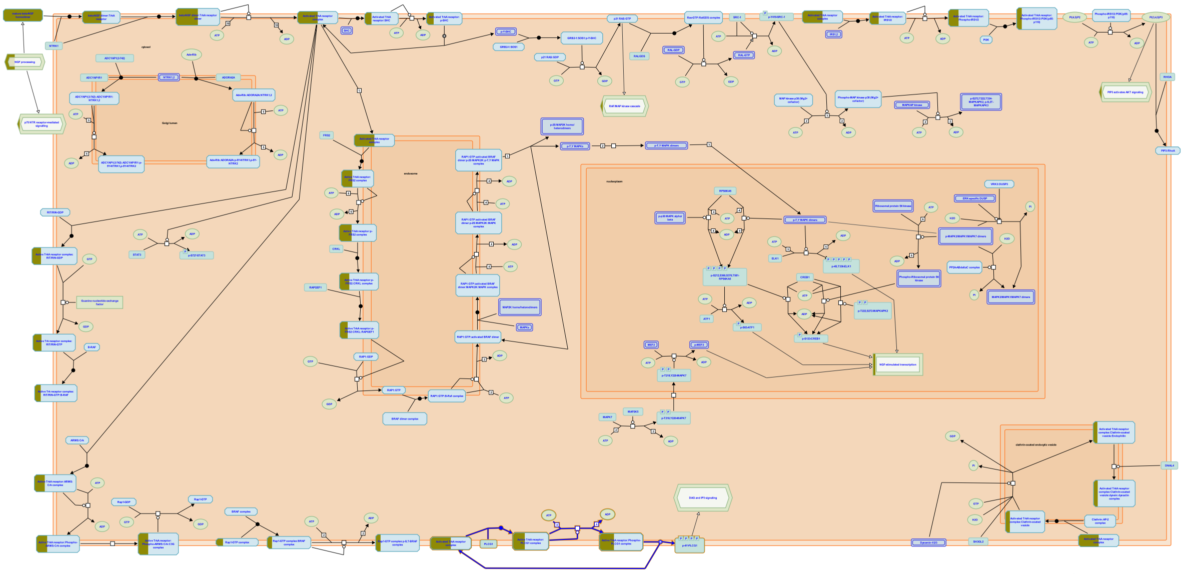

The activation of phosphlipase C-gamma (PLC-gamma) and subsequent mobilization of calcium from intracellular stores are essential for neurotrophin secretion. PLC-gamma is activated through the phosphorylation by TrkA receptor kinase and this form hydrolyses PIP2 to generate inositol tris-phosphate (IP3) and diacylglycerol (DAG). IP3 promotes the release of Ca2+ from internal stores and this results in activation of enzymes such as protein kinase C and Ca2+ calmodulin-regulated protein kinases.

References

Reichardt LF & Huang EJ (2003). Trk receptors: roles in neuronal signal transduction. Annu Rev Biochem, 72, 609-42.

Ji Q & Carpenter G (1999). Phospholipase C-gamma as a signal-transducing element. Exp Cell Res, 253, 15-24.

Edit history

Date	Action	Author
2006-02-07	Created	
2006-10-10	Edited	Jassal B
2006-10-10	Authored	Annibali D, Nasi S
2007-11-08	Reviewed	Greene LA
2017-05-23	Modified	Rothfels K

1 submitted entities found in this pathway, mapping to 1 Reactome entities

Input	UniProt Id
NGF	P01138

4. NFG and proNGF binds to p75NTR (R-HSA-205017)

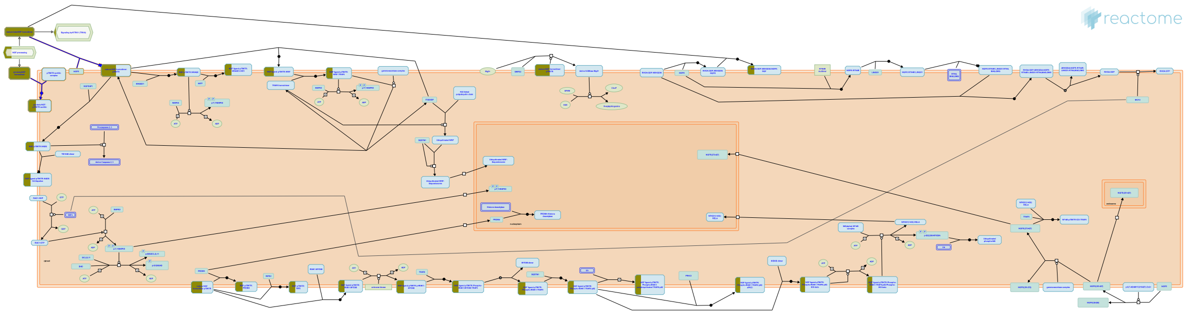

When the co-receptor sortilin is present at the cell surface, proNGF preferentially interacts with a p75NTR:sortilin complex. Thus, proNGF, which does not bind TRKA, discriminates between TRKA and p75NTR, in cells that express both receptors. The same is true for proBDNF. Pro-neurotrophin binding to p75NTR:sortilin activates an apoptotic cascade, which may be involved in cell death after injury, and in neurodegenerative diseases such as Alzheimer's dementia.

References

Edit history

Date	Action	Author
2006-10-10	Authored	Annibali D, Nasi S
2007-12-06	Created	Jassal B
2008-05-20	Edited	Jassal B
2008-05-20	Reviewed	Friedman WJ
2008-05-28	Reviewed	Chao MV
2022-05-20	Modified	Weiser JD

1 submitted entities found in this pathway, mapping to 1 Reactome entities

Input	UniProt Id
NGF	P01138

5. Signalling to STAT3 (R-HSA-198745)

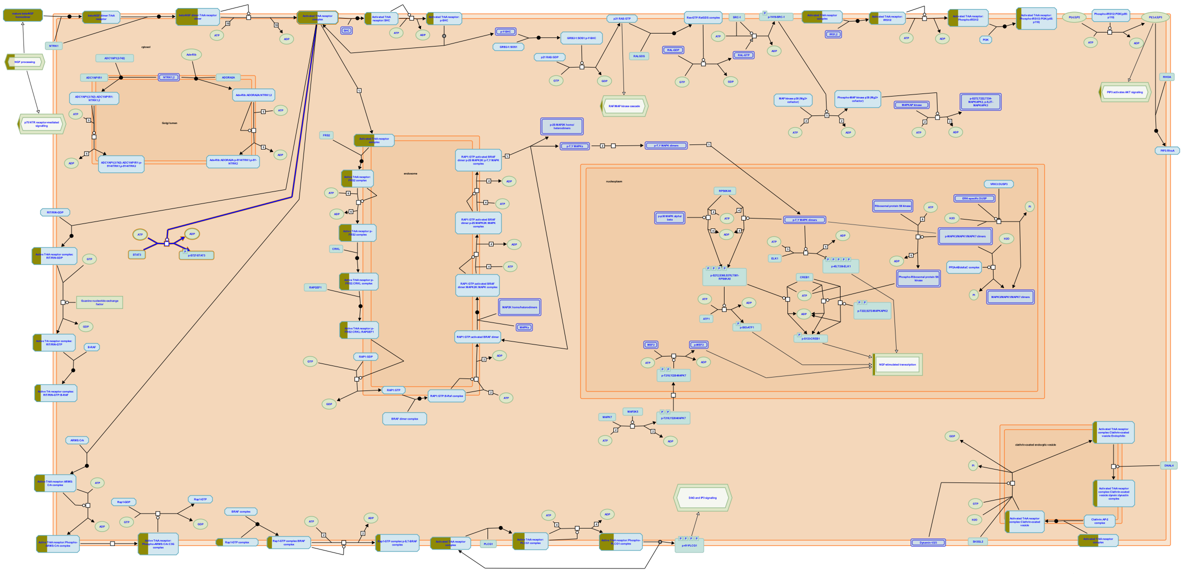

Neurotrophin-induced increase in Signal transducer and activator of transcription 3 (STAT3; acute-phase response factor) activation appears to underly several downstream functions of neurotrophin signalling, such as transcription of immediate early genes, proliferation arrest, and neurite outgrowth.

References

Edit history

Date	Action	Author
2006-10-10	Authored	Annibali D, Nasi S
2007-07-10	Created	
2007-11-08	Reviewed	Greene LA
2011-10-24	Modified	Jassal B

1 submitted entities found in this pathway, mapping to 1 Reactome entities

Input	UniProt Id
NGF	P01138

6. ABO blood group biosynthesis (R-HSA-9033807)

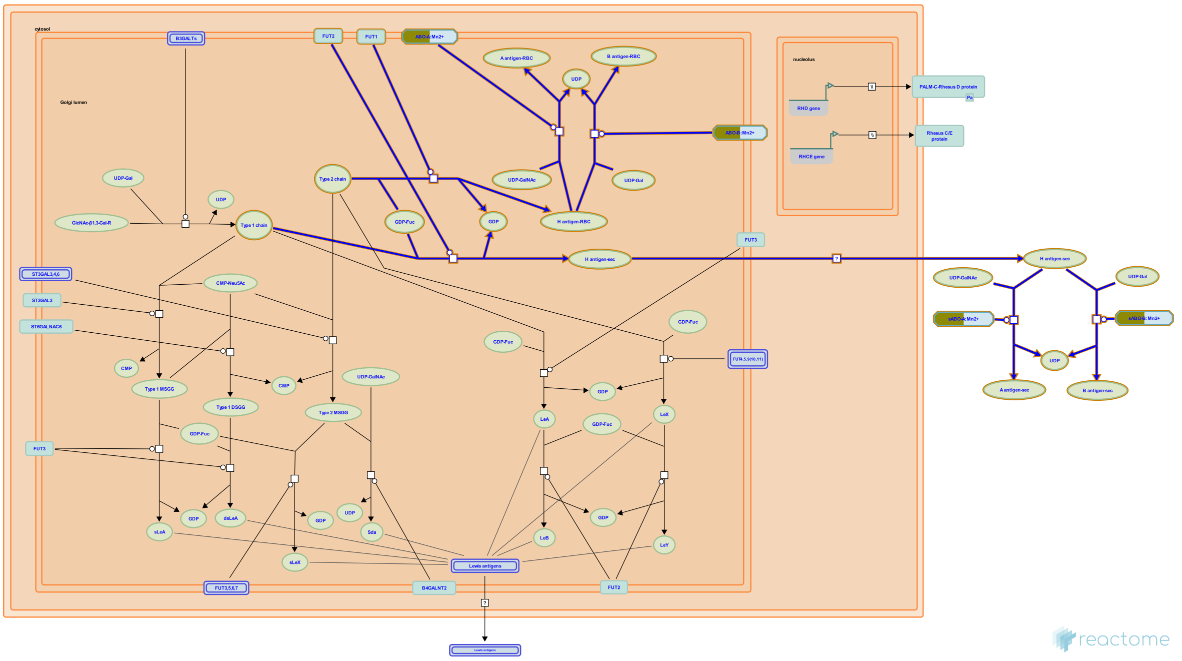

Perhaps the most important and widely studied blood group is the ABO blood group. It consists of antigens found on the outer surface of red cells and corresponding antibodies in plasma. The majority of the world's population (~80%) are 'secretors' which means that the antigens present in their blood will also be found in other body fluids such as saliva. An individual can be a Secretor (Se) or a non-secretor (se) and this is completely independent of whether the individual is of blood type A, B, AB, or O. From a very early age, the immune system develops antibodies against whichever ABO blood group antigens are not found on the individual's RBCs. Thus, a blood group A individual will have anti-B antibodies and a blood group B individual will have anti-A antibodies. Individuals with the most common blood group, O, will have both anti-A and anti-B in their plasma. Blood group AB is the least common, and these individuals will have neither anti-A nor anti-B in their plasma.

The primary structure of these antigens is an oligosaccharide precursor sequence on to which one or more sugars are attached at specific locations. The blood group oligosaccharide antigens A, B and H are produced by enzymes expressed by these genes and form the basis of the ABO 'blood type' phenotypes. A and B antigens were originally identified on red blood cells (RBCs) but later identified on other cell types and in bodily secretions. The ABO blood group system is important in blood transfusion, cell/tissue/organ transplantation and forensic evidence at crime scenes.

The H antigen is formed with the addition of a fucose sugar onto one of two precursor oligosaccharide sequences (Type 1 chains are Gal 1,3 GlcNAc 1,3 Gal R and Type 2 chains are Gal 1,4 GlcNAc 1,3 Gal R; where R is a glycoprotein (Type 1) or glycosphingolipid (Type 2). Type 2 chains are only found on RBCs, epithelial cells and endothelial cells. The *H* gene expressed in hematopoietic cells produces -1,2-fucosyltransferase 1 (FUT1) which adds a fucose to Type 2 chains to form the H antigen in non-secretors. Type 1 chains are found in secretors. The *Se* gene expressed in secretory glands produces -1,2-fucosyltransferase 2 (FUT2) which adds a fucose to Type 1 chains to form the H antigen in secretors.

The H antigen is abundant in individuals with blood group O and is the essential precursor for the production of A and B antigens. A and B antigens are formed by the action of glycosyltransferases encoded by functional alleles at the ABO genetic locus. The co dominant A allele encodes A transferase, which transfers an N acetylgalactosamine (GalNAc) sugar to the H antigen forming the A antigen. Similarly, the co dominant B allele encodes B transferase, which transfers a galactose (Gal) sugar to the H antigen forming the B antigen. Individuals who have both A and B alleles form the AB antigen. Individuals who are homozygous for the recessive O allele express the H antigen but do not form A or B antigens as they lack both the glycosyltransferase enzymes for their formation. Mutant alleles of the corresponding FUT1 or FUT2 genes result in either a H– phenotype (Bombay phenotype, Oh) or a weak H phenotype (para Bombay) where the affected individual cannot form H, A or B antigens (Kaneko et al. 1997, Koda et al. 1997). The biosyntheses of the A, B and H antigens are described in this section (Ewald & Sumner 2016, Scharberg et al. 2016).

References

Ewald DR & Sumner SC (2016). Blood type biochemistry and human disease. Wiley Interdiscip Rev Syst Biol Med, 8, 517-535.

Scharberg EA, Olsen C & Bugert P (2016). The H blood group system. Immunohematology, 32, 112-118.

Shinya N, Kudo T, Okubo Y, Seno T, Iwasaki H, Kaneko M, ... Nishihara S (1997). Wide variety of point mutations in the H gene of Bombay and para-Bombay individuals that inactivate H enzyme. Blood, 90, 839-49.

Johnson PH, Soejima M, Koda Y, Smart E & Kimura H (1997). Missense mutation of FUT1 and deletion of FUT2 are responsible for Indian Bombay phenotype of ABO blood group system. Biochem. Biophys. Res. Commun., 238, 21-5.

Edit history

Date	Action	Author
2017-12-28	Edited	Jassal B
2017-12-28	Authored	Jassal B
2017-12-28	Created	Jassal B
2018-12-17	Reviewed	Matsui T
2022-05-20	Modified	Weiser JD

1 submitted entities found in this pathway, mapping to 1 Reactome entities

Input	UniProt Id
ABO	P16442

7. Ceramide signalling (R-HSA-193681)

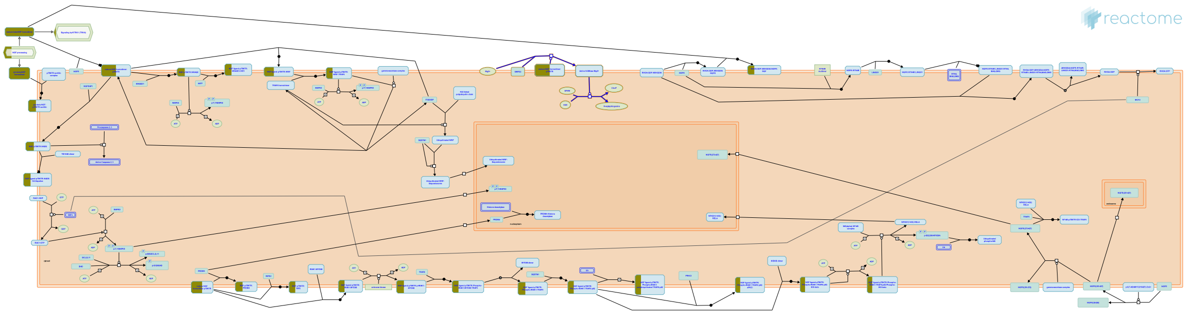

In certain cell types, ligand binding to p75NTR leads to ceramide production, which can mediate either cell survival (e.g. in noecotical subplate neurons) or apoptosis (e.g. in oligodendrocytes). Low levels of ceramide are also able to stimulate axonal outgrowth in hippocampal neurons.

References

Reichardt LF & Huang EJ (2003). Trk receptors: roles in neuronal signal transduction. Annu Rev Biochem, 72, 609-42.

Edit history

Date	Action	Author
2006-10-10	Authored	Annibali D, Nasi S
2007-02-23	Created	Jassal B
2008-05-20	Edited	Jassal B
2008-05-20	Reviewed	Friedman WJ
2008-05-28	Reviewed	Chao MV
2016-09-24	Modified	D'Eustachio P

1 submitted entities found in this pathway, mapping to 1 Reactome entities

Input	UniProt Id
NGF	P01138

8. Expression and Processing of Neurotrophins (R-HSA-9036866)

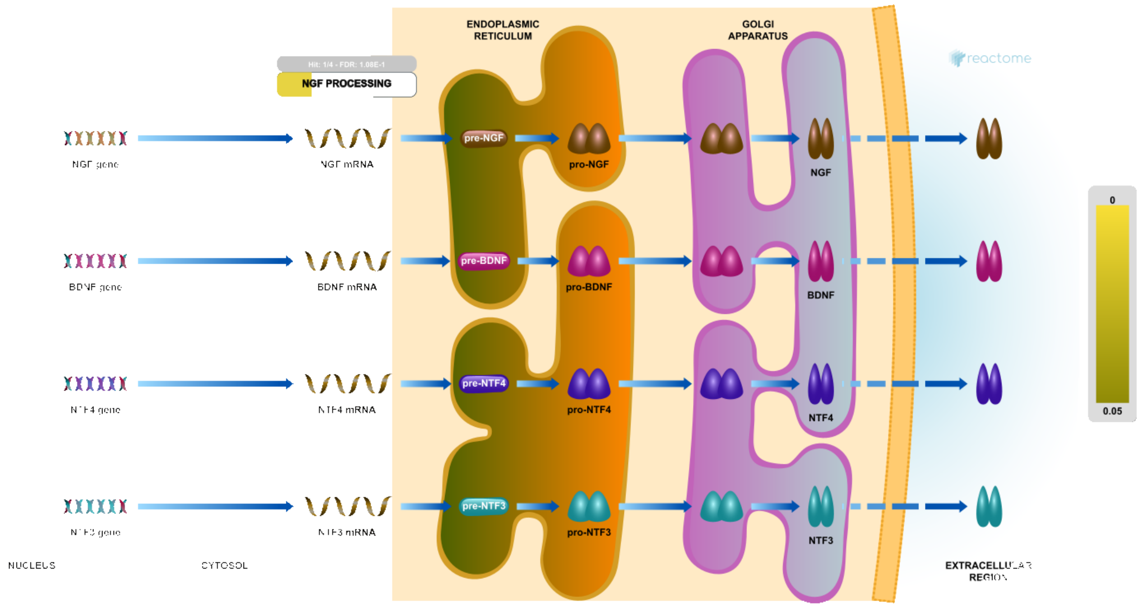

Neurotrophins function as ligands for receptor tyrosine kinases of the NTRK (TRK) family, as well as the death receptor NGFR (p75NTR). While all four neurotrophins, NGF, BDNF, NTF3 (NT-3) and NTF4 (NT-4, NT-5, NTF5) can bind to and activate NGFR, they show different specificity for NTRKs. NGF exclusively activates NTRK1 (TRKA). BDNF and NTF4 are high affinity ligands for NTRK2 (TRKB). NTF3 is a high affinity ligand for NTRK3 (TRKC) and a low affinity ligand for NTRK2. Neurotrophins play pivotal roles in survival, differentiation, and plasticity of neurons in the peripheral and central nervous system. They are produced, and secreted in minute amounts, by a variety of tissues. For review, please refer to Lessmann et al. 2003, Chao 2003, and Park and Poo 2013.

Human NGF, also knowns as the nerve growth factor, is encoded by a gene on chromosome 1, which produces a single transcript. Nascent NGF protein, pre-pro-NGF, is 241 amino acids long. As pre-pro-NGF enters the endoplasmic reticulum (ER), the signal peptide, consisting of eighteen amino acids at the N-terminus, is cleaved, producing pro-NGF. Two molecules of pro-NGF form homodimers in the ER. After transport of pro-NGF homodimers to the Golgi, 103 amino acids at the N-terminus of pro-NGF are cleaved, producing mature NGF homodimers. Both pro-NGF homodimers and mature NGF homodimers are secreted to the extracellular space. Mature NGF homodimers activate NTRK1 signaling, while NGFR signaling can be activated by both mature and pro-NGF homodimers. Secreted pro-NGF homodimers may be cleaved by extracellular matrix proteases to produce mature NGF homodimers. For review, please refer to Poo 2001, Lu et al. 2005, Skaper et al. 2012, Bradshaw et al. 2015.

Human BDNF, also known as brain-derived neurotrophic factor, is encoded by a gene on chromosome 11, which, through the use of 9 alternative promoters and alternative splicing, produces 17 protein-coding transcripts. Most BDNF transcripts result in the same pre-pro-BDNF protein of 247 amino acids, but alternative promoters and different 5' and 3'UTRs allow to fine-tune regulation of BDNF expression at different developmental stages and at different levels of neuronal activity. Similar to NGF, pre-pro-BDNF is processed by proteolytic cleavage in the ER to produce pro-BDNF homodimers. It is unclear whether proteolytic processing of pro-BDNF, to produce mature BDNF homodimers, occurs in the Golgi or in the secretory granules. Extracellular matrix proteases can also cleave secreted pro-BDNF to produce mature BDNF homodimers. Secreted mature BDNF homodimers can activate NTRK2 signaling, while secreted pro-BDNF homodimers can activate NGFR signaling. For review, please refer to Poo 2001, Lu et al. 2005, Skaper et al. 2012, Park and Poo 2013.

Human NTF4, also known as neurotrophin-4, is transcribed from a gene on chromosome 19. A single experimentally confirmed transcript produces a pre-pro-NTF4 protein of 210 amino acids. After proteolytic processing in the ER and Golgi, mature NTF4 homodimers are secreted and can activate NTRK2 signaling (Hibbert et al. 2003). For review, please refer to Poo 2001, Skaper et al. 2012.

Human NTF3, also known as neurotrophin-3, is transcribed from a gene on chromosome 12. Two NTF3 transcripts have been experimentally confirmed, but only the longer NTF3 splice variant of 270 amino acids has been studied. After proteolytic processing in the ER and Golgi, mature NTF3 homodimers are secreted and can activate NTRK3 signaling (Seidah et al. 1996, Farhadi et al. 2000). For review, please refer to Poo 2001, Skaper et al. 2012.

References

Poo MM & Park H (2013). Neurotrophin regulation of neural circuit development and function. Nat. Rev. Neurosci., 14, 7-23.

Pang PT, Woo NH & Lu B (2005). The yin and yang of neurotrophin action. Nat. Rev. Neurosci., 6, 603-14.

Chao MV (2003). Neurotrophins and their receptors: a convergence point for many signalling pathways. Nat Rev Neurosci, 4, 299-309.

Pundavela J, Hondermarck H, Chalkley RJ, Bradshaw RA, Burlingame AL & Biarc J (2015). NGF and ProNGF: Regulation of neuronal and neoplastic responses through receptor signaling. Adv Biol Regul, 58, 16-27.

Skaper SD (2012). The neurotrophin family of neurotrophic factors: an overview. Methods Mol. Biol., 846, 1-12.

Edit history

Date	Action	Author
2018-02-10	Edited	Orlic-Milacic M
2018-02-10	Authored	Orlic-Milacic M
2018-02-10	Created	Orlic-Milacic M
2022-05-20	Modified	Weiser JD

1 submitted entities found in this pathway, mapping to 1 Reactome entities

Input	UniProt Id
NGF	P01138

9. NGF processing (R-HSA-167060)

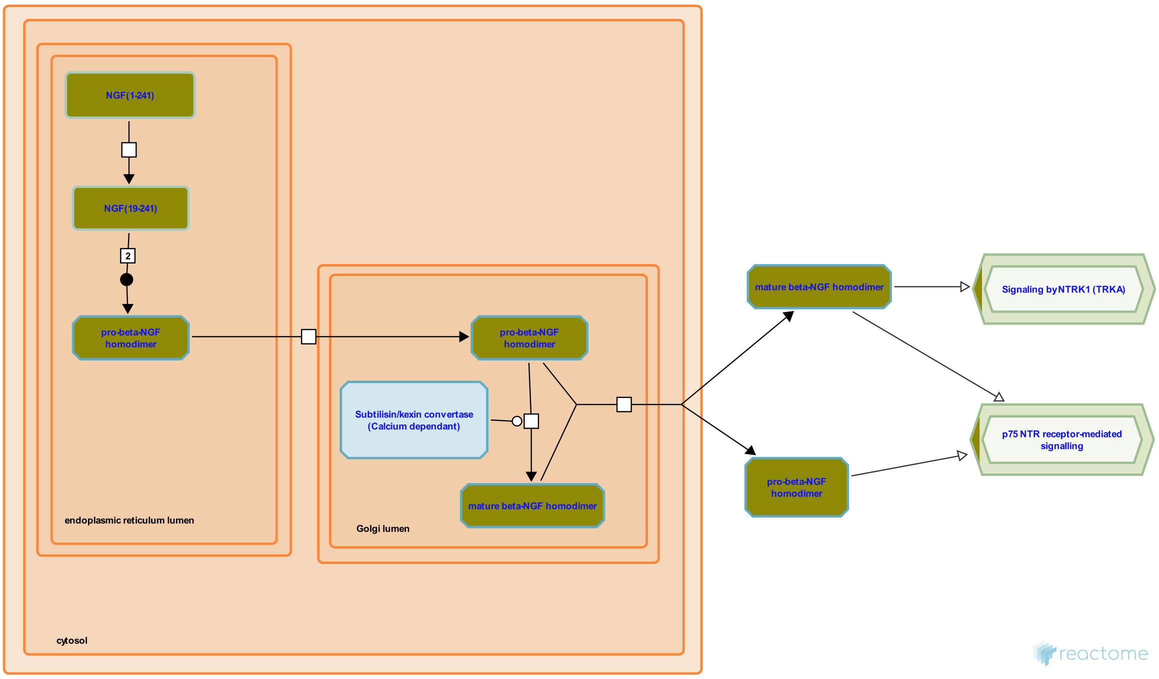

All neurotrophins (NTs) are generated as pre-pro-neurotrophin precursors. The signal peptide is cleaved off as NT is associated with the endoplasmic reticulum (ER). The resulting pro-NT can form a homodimer spontaneously which then transits to the Golgi apparatus and then onto the trans-Golgi network (TGN). Resident protein convertases (PCs) can cleave off the pro-sequence and mature NT is is targeted to constitutively released vesicles. The pro-NT form can also be released to the extracellular region.

References

Lessmann V, Gottmann K & Malcangio M (2003). Neurotrophin secretion: current facts and future prospects. Prog Neurobiol, 69, 341-74.

Edit history

Date	Action	Author
2006-02-07	Created	
2006-10-10	Edited	Jassal B
2006-10-10	Authored	Annibali D, Nasi S
2007-11-08	Reviewed	Greene LA
2022-05-20	Modified	Weiser JD

1 submitted entities found in this pathway, mapping to 1 Reactome entities

Input	UniProt Id
NGF	P01138

10. Axonal growth stimulation (R-HSA-209563)

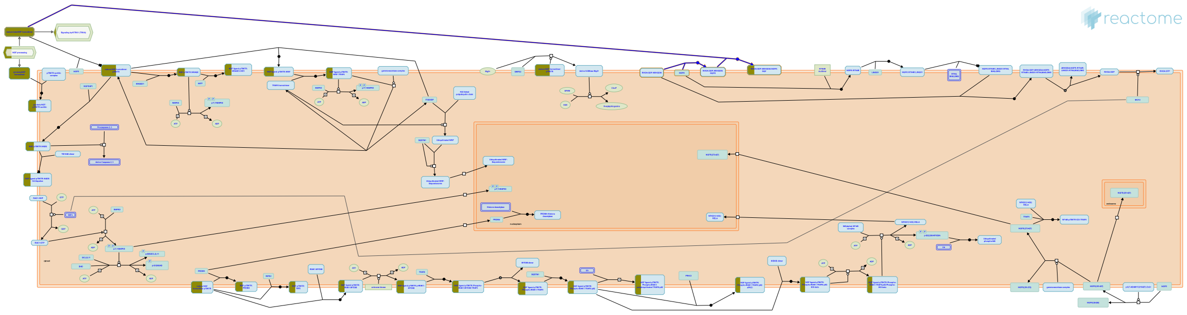

Complex formation between p75NTR and RHOA can leads to inhibition of RHOA activity and axonal growth.

References

Mimura F, Yamashita T, Fujitani M, Hata K & Yamagishi S (2005). Multiple signals regulate axon regeneration through the Nogo receptor complex. Mol Neurobiol, 32, 105-11.

Edit history

Date	Action	Author
2006-10-10	Authored	Annibali D, Nasi S
2007-12-19	Created	Jassal B
2008-05-20	Edited	Jassal B
2008-05-20	Reviewed	Friedman WJ
2008-05-28	Reviewed	Chao MV
2022-05-20	Modified	Weiser JD

1 submitted entities found in this pathway, mapping to 1 Reactome entities

Input	UniProt Id
NGF	P01138

11. Signalling to p38 via RIT and RIN (R-HSA-187706)

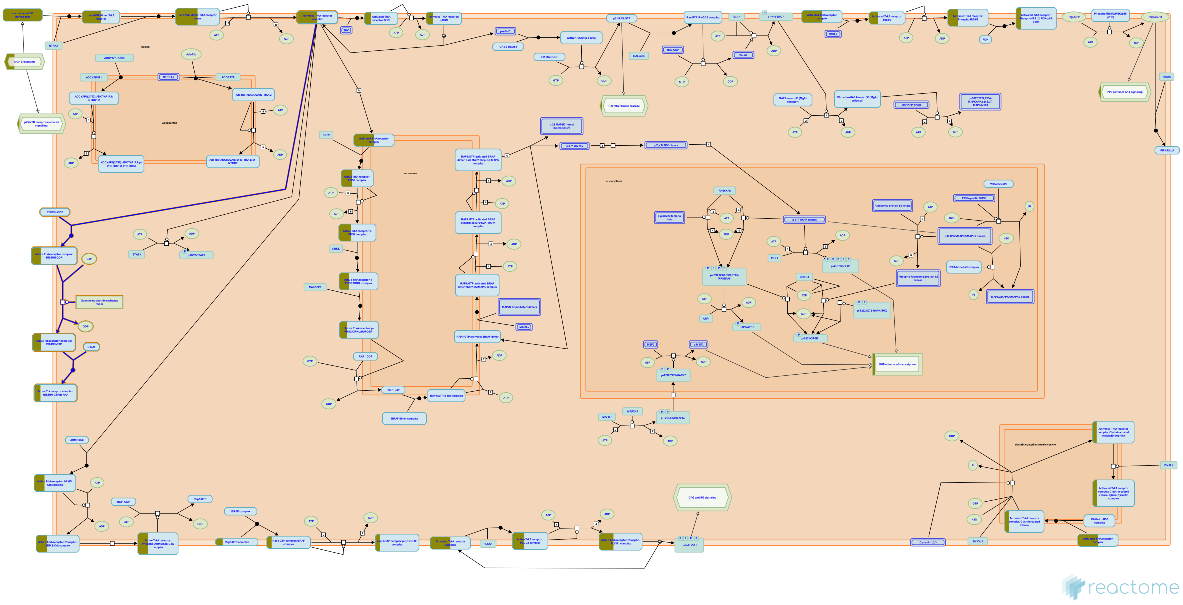

RIT and RIN are two small guanine nucleotide binding proteins that share more than 50% sequence identity with RAS, including highly conserved core effector domains. Unlike RAS, the C termini of RIT and RIN lack a typical prenylation motif (CAAX, XXCC, or CXC) required for the association of RAS proteins with the plasma membrane. RIT is expressed in all tissues, whereas RIN is neuron-specific. They have similar signalling properties and are activated by NGF through unknown exchange factors. They signal to ERKs and p38 MAP kinase. They mainly lead to p38 activation via the BRAF-MEK kinase cascade.

References

Der CJ & Reuther GW (2000). The Ras branch of small GTPases: Ras family members don't fall far from the tree. Curr Opin Cell Biol, 12, 157-65.

Edit history

Date	Action	Author
2006-09-21	Created	Jassal B
2006-10-10	Edited	Jassal B
2006-10-10	Authored	Annibali D, Nasi S
2007-11-08	Reviewed	Greene LA
2017-05-23	Modified	Rothfels K

1 submitted entities found in this pathway, mapping to 1 Reactome entities

Input	UniProt Id
NGF	P01138

12. NADE modulates death signalling (R-HSA-205025)

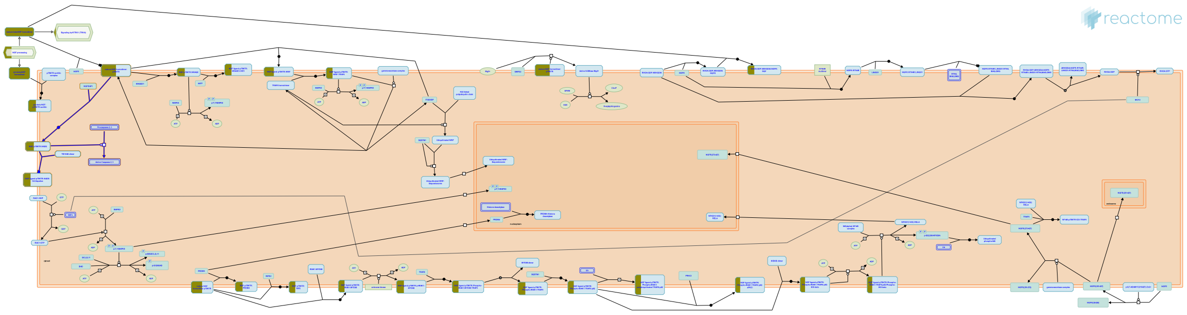

NADE protein (p75NTR-associated cell death executor) may induce cell death upon NGF binding, but not BDNF, NT3, or NT4/5 binding, to p75NTR. The NADE-dependent apoptosis is modulated by the 14-3-3-epsilon protein (Kimura MT et al, 2001).

References

Irie S, Sato TA, Mukai J, Shoji-Hoshino S, Oshimura M, Nadano D & Kimura MT (2001). 14-3-3 is involved in p75 neurotrophin receptor-mediated signal transduction. J Biol Chem, 276, 17291-300.

Edit history

Date	Action	Author
2006-10-10	Authored	Annibali D, Nasi S
2007-12-06	Created	Jassal B
2008-05-20	Edited	Jassal B
2008-05-20	Reviewed	Friedman WJ
2008-05-28	Reviewed	Chao MV
2022-05-20	Modified	Weiser JD

1 submitted entities found in this pathway, mapping to 1 Reactome entities

Input	UniProt Id
NGF	P01138

13. PTK6 promotes HIF1A stabilization (R-HSA-8857538)

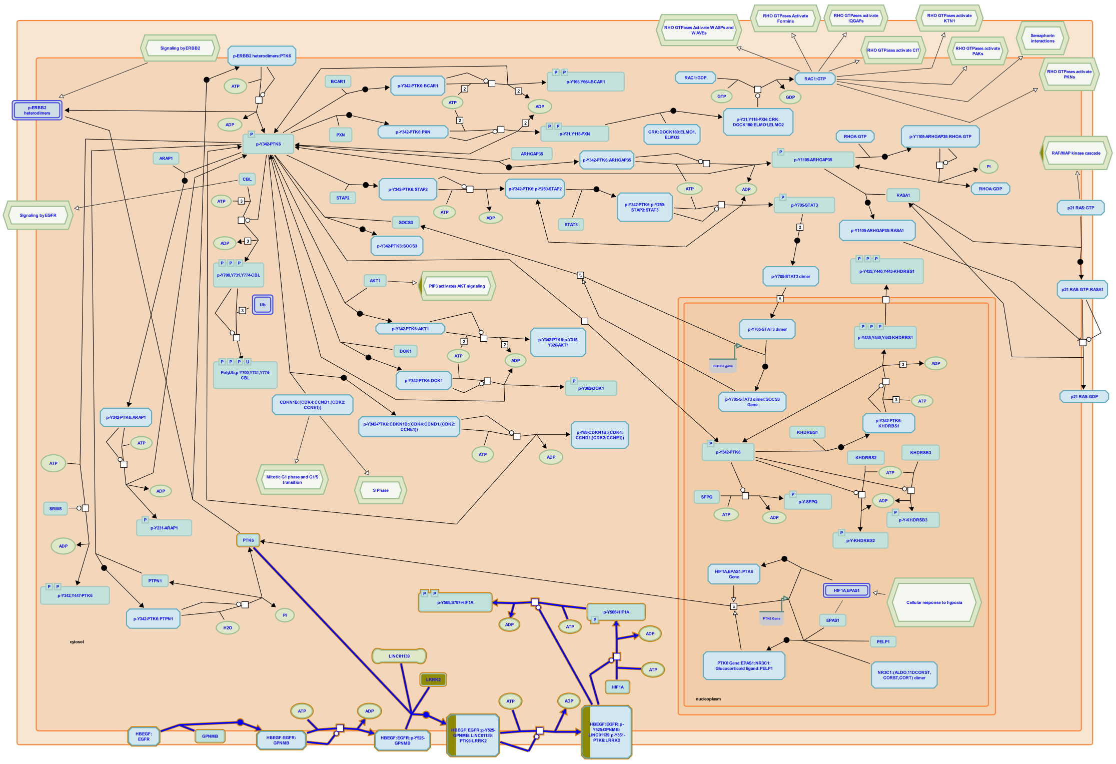

HBEGF-stimulated formation of EGFR heterodimers with GPNMB triggers PTK6-mediated phosphorylation and stabilization of the hypoxia inducible factor 1 alpha (HIF1A) under normoxic conditions. This process depends on the presence of a long non-coding RNA LINC01139 (LINK-A) (Lin et al. 2016).

References

Marks JR, Yang L, Zhou Y, Ma G, Wang S, Xing Z, ... Hawke DH (2016). The LINK-A lncRNA activates normoxic HIF1 signalling in triple-negative breast cancer. Nat. Cell Biol., 18, 213-24.

Edit history

Date	Action	Author
2016-02-07	Reviewed	Pires IM
2016-02-11	Edited	Orlic-Milacic M
2016-02-11	Authored	Orlic-Milacic M
2016-02-15	Created	Orlic-Milacic M
2022-06-07	Modified	Weiser JD

1 submitted entities found in this pathway, mapping to 1 Reactome entities

Input	UniProt Id
LRRK2	Q5S007

14. p75NTR negatively regulates cell cycle via SC1 (R-HSA-193670)

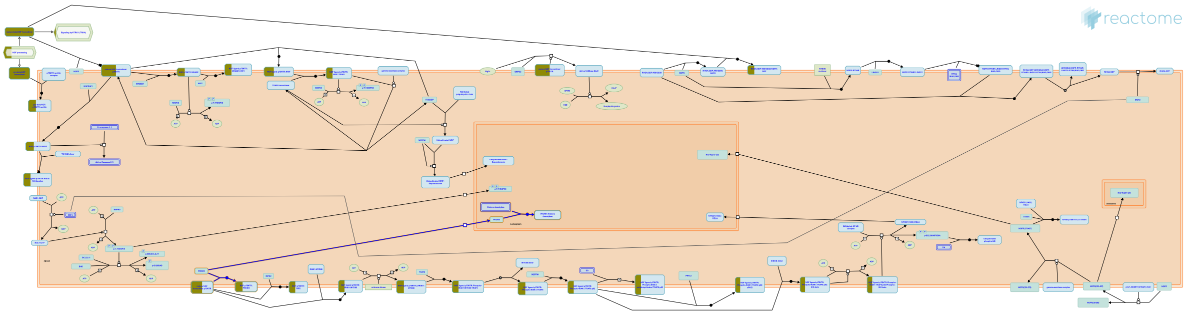

SC1 (Schwann Cell factor 1; also called PR domain zinc finger protein 4, PRDM4) interacts with an NGF:p75NTR complex and signals cell cycle arrest by regulating the levels of cyclin E.

References

Frade JM & Lopez-Sanchez N (2002). Control of the cell cycle by neurotrophins: lessons from the p75 neurotrophin receptor. Histol Histopathol, 17, 1227-37.

Edit history

Date	Action	Author
2006-10-10	Authored	Annibali D, Nasi S
2007-02-23	Created	Jassal B
2008-05-20	Edited	Jassal B
2008-05-20	Reviewed	Friedman WJ
2008-05-28	Reviewed	Chao MV
2010-03-05	Modified	Jassal B

1 submitted entities found in this pathway, mapping to 1 Reactome entities

Input	UniProt Id
NGF	P01138

15. ARMS-mediated activation (R-HSA-170984)

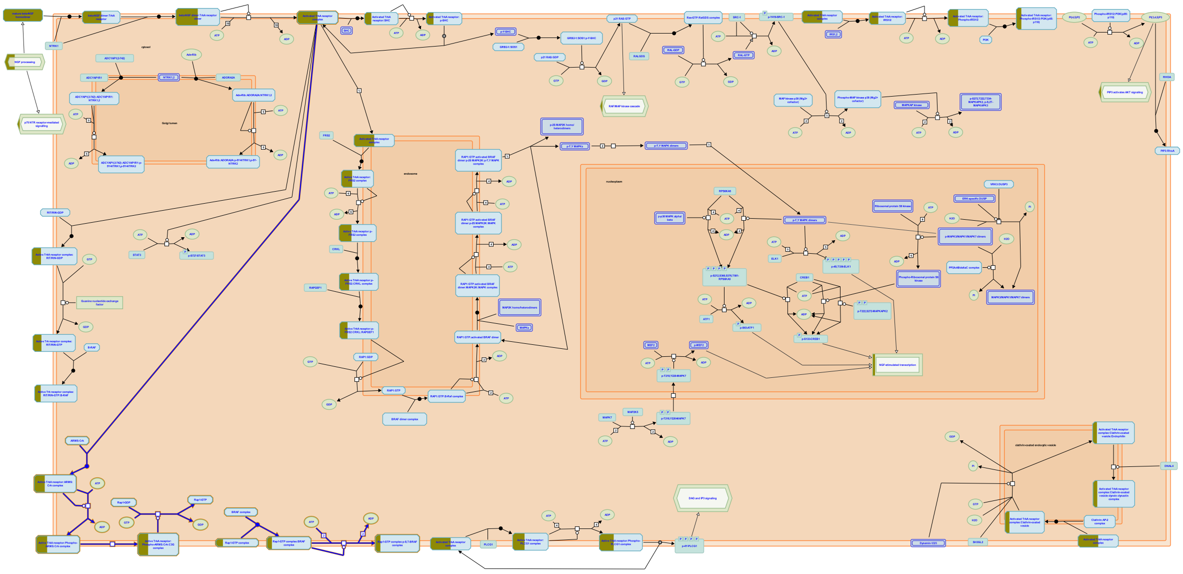

ARMS (Ankyrin-Rich Membrane Spanning/Kidins 220) is a 220kD tetraspanning adaptor protein which becomes rapidly tyrosine phosphorylated by active Trk receptors. ARMS is another adaptor protein which is involved in the activation of Rap1 and the subsequent prolonged activation of the MAPK cascade.

References

Miller FD & Kaplan DR (1997). Signal transduction by the neurotrophin receptors. Curr Opin Cell Biol, 9, 213-21.

Edit history

Date	Action	Author
2006-01-25	Created	Jassal B
2006-10-10	Edited	Jassal B
2006-10-10	Authored	Annibali D, Nasi S
2007-11-08	Reviewed	Greene LA
2022-05-20	Modified	Weiser JD

1 submitted entities found in this pathway, mapping to 1 Reactome entities

Input	UniProt Id
NGF	P01138

16. Activation of TRKA receptors (R-HSA-187015)

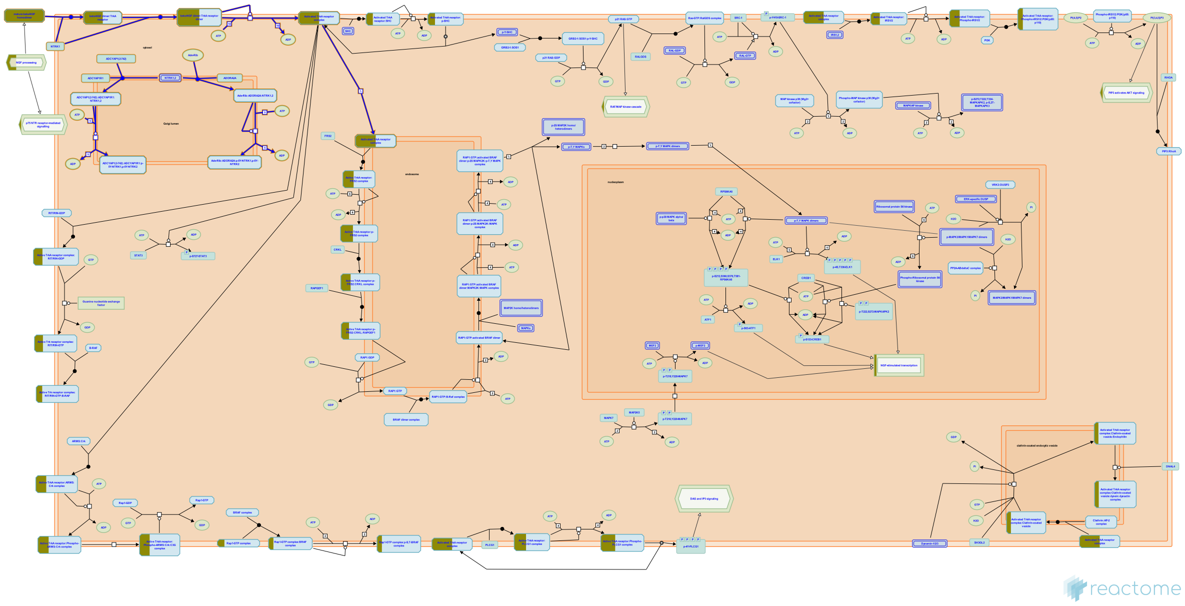

Trk receptors can either be activated by neurotrophins or by two G-protein-coupled receptors (GP-CRs) although the biological relevance of GPCRs remains to be shown.

References

Reichardt LF (2006). Neurotrophin-regulated signalling pathways. Philos Trans R Soc Lond B Biol Sci, 361, 1545-64.

Edit history

Date	Action	Author
2006-09-07	Created	Jassal B
2006-10-10	Edited	Jassal B
2006-10-10	Authored	Annibali D, Nasi S
2007-11-08	Reviewed	Greene LA
2022-05-20	Modified	Weiser JD

1 submitted entities found in this pathway, mapping to 1 Reactome entities

Input	UniProt Id
NGF	P01138

17. Coenzyme A biosynthesis (R-HSA-196783)

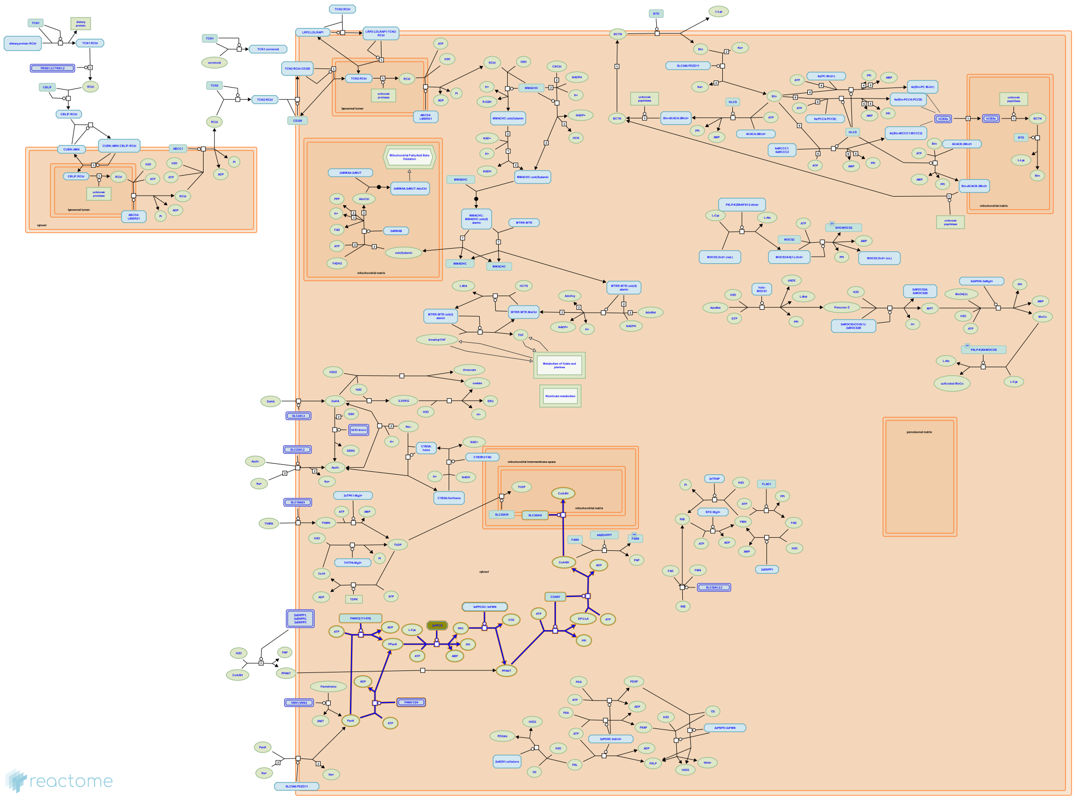

Coenzyme A (CoA) is a ubiquitous cofactor that functions as an acyl group carrier in diverse processes including fatty acid metabolism and the TCA cycle (Lipmann 1953). It is synthesized from the vitamin pantothenate in a sequence of five reactions (Daugherty et al. 2002; Leonardi et al. 2005; Robishaw and Neely 1985). These reactions all occur in the cytosol or the mitochondrial intermembrane space (Leonardi et al. 2005). A recently described transport protein appears to mediate the uptake of Coenzyme A into the mitochondrial matrix (Prohl et al. 2001).

References

Prohl C, Lill R, Kispal G, Diekert K, Bedekovics T, Pelzer W & Kmita H (2001). The yeast mitochondrial carrier Leu5p and its human homologue Graves' disease protein are required for accumulation of coenzyme A in the matrix. Mol Cell Biol, 21, 1089-97.

Leonardi R, Zhang YM, Rock CO & Jackowski S (2005). Coenzyme A: back in action. Prog Lipid Res, 44, 125-53.

Lipmann F (1953). On chemistry and function of coenzyme A. Bacteriol Rev, 17, 1-16.

Robishaw JD & Neely JR (1985). Coenzyme A metabolism. Am J Physiol, 248, E1-9.

Osterman A, Farrell M, Daugherty M, de Crecy-Lagard V, Scholle M, Polanuyer B & Lykidis A (2002). Complete reconstitution of the human coenzyme A biosynthetic pathway via comparative genomics. J Biol Chem, 277, 21431-9.

Edit history

Date	Action	Author
2007-04-24	Edited	Jassal B
2007-04-24	Authored	Jassal B

Date	Action	Author
2007-04-24	Created	Jassal B
2007-07-10	Reviewed	D'Eustachio P
2022-05-21	Modified	Weiser JD

1 submitted entities found in this pathway, mapping to 1 Reactome entities

Input	UniProt Id
PPCS	Q9HAB8

18. PI3K/AKT activation (R-HSA-198203)

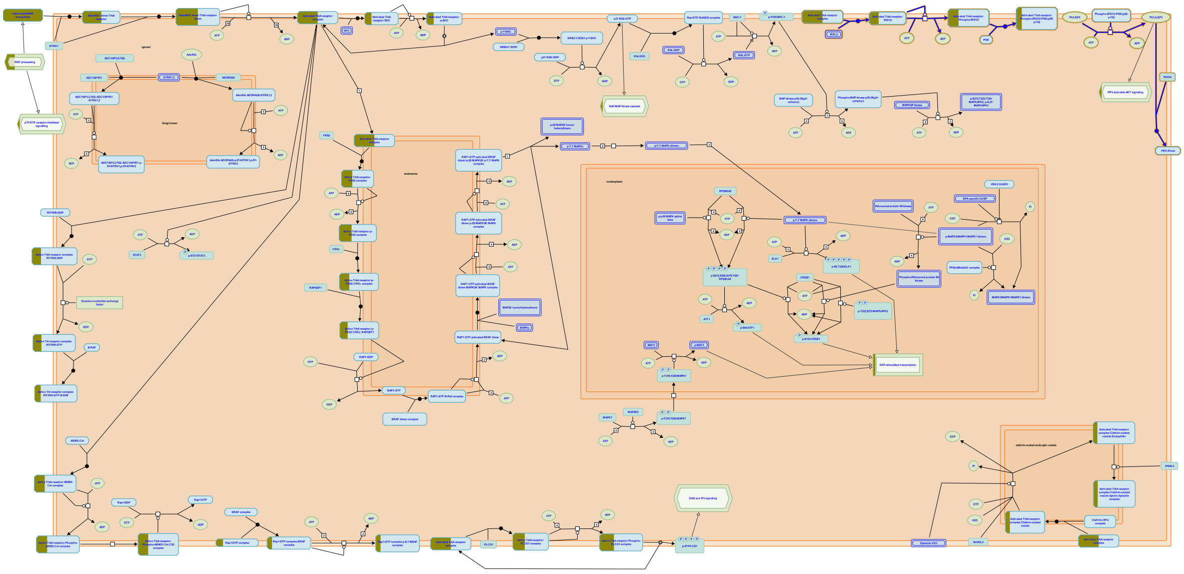

PI3K/AKT signalling is a major regulator of neuron survival. It blocks cell death by both impinging on the cytoplasmic cell death machinery and by regulating the expression of genes involved in cell death and survival. In addition, it may also use metabolic pathways to regulate cell survival.The PI3K/AKT pathway also affects axon diameter and branching (Marcus et al, 2002) and regulates small G proteins like RhoA (Vanhaesebroeck, B and Waterman, MD, 1999), which control the behaviour of the F-actin cytoskeleton. Moreover, through its connection with the TOR pathway, it promotes translation of a subset of mRNAs.

References

Snider WD, Zhong J & Markus A (2002). Raf and akt mediate distinct aspects of sensory axon growth . Neuron, 35, 65-76.

Waterfield MD & Vanhaesebroeck B (1999). Signaling by distinct classes of phosphoinositide 3-kinases. Exp Cell Res, 253, 239-54.

Edit history

Date	Action	Author
2006-10-10	Authored	Annibali D, Nasi S
2007-05-21	Created	Jassal B
2007-11-08	Reviewed	Greene LA
2022-06-07	Modified	Weiser JD

1 submitted entities found in this pathway, mapping to 1 Reactome entities

Input	UniProt Id
NGF	P01138

19. p75NTR regulates axonogenesis (R-HSA-193697)

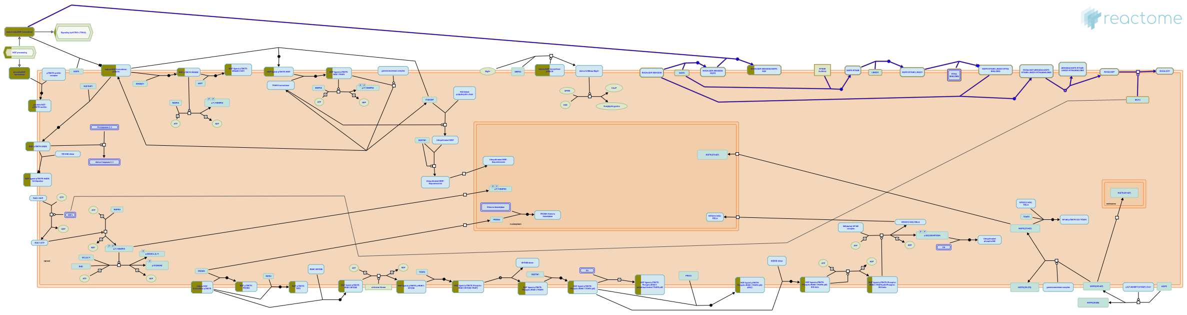

p75NTR modulates axonal growth by regulating the activity of small GTPases like RHOA and RHOB, that control the state of actin polymerization. The best studied is RHOA. In its active, GTP-bound form, RHOA rigidifies the actin cytoskeleton, thereby inhibiting axonal elongation and causing growth cone collapse. Depending on the ligand that binds to it, p75NTR can either promote or inhibit axonal growth, Neurotrophin binding leads to inhibition of RHOA activity and axonal growth. Axonal growth inhibition is caused by myelin molecules named MDGIs (myelin-derived growth inhibitors), such as NOGO, MAG, OMGP. MDGIs bind to a complex made up of p75NTR and the NOGO receptor, causing RHOA activation and axonal growth inhibition.

References

Li HY & Zhou XF (2007). Roles of glial p75NTR in axonal regeneration. J Neurosci Res, 85, 1601-5.

Edit history

Date	Action	Author
2006-10-10	Authored	Annibali D, Nasi S
2007-02-23	Created	Jassal B
2008-05-20	Edited	Jassal B
2008-05-20	Reviewed	Friedman WJ
2008-05-28	Reviewed	Chao MV
2022-05-20	Modified	Weiser JD

1 submitted entities found in this pathway, mapping to 1 Reactome entities

Input	UniProt Id
NGF	P01138

20. Sodium/Calcium exchangers (R-HSA-425561)

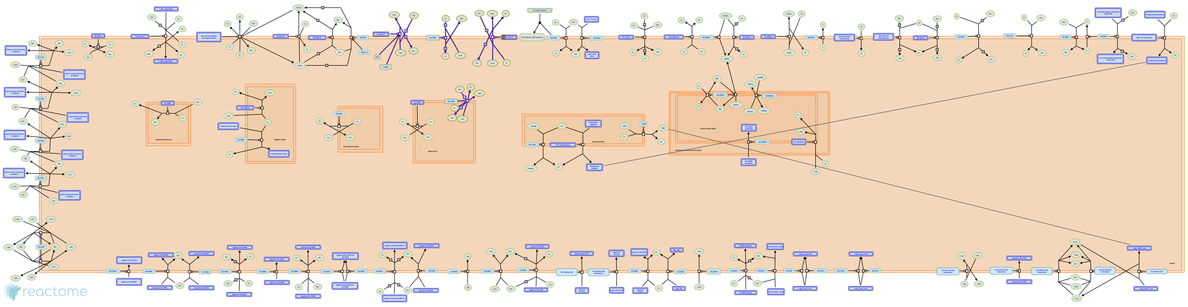

Calcium ions are used by cells as ubiquitous signalling molecules that control diverse physiological events. Three mammalian gene families control Ca2+ transport across plasma membranes and intracellular compartments (Lytton J, 2007). They are the Na+/Ca2+ exchanger family designated NCX (SLC8) (three members NCX1-3) (Quednau BD et al, 2004), the Na+/Ca2+-K+ exchanger family designated NCKX (SLC24) (five members NCKX1-5) (Schnetkamp PP, 2004) and a Ca2+/cation exchanger (NCKX6, NCLX) whose physiological function remains unclear.

References

Lytton J (2007). Na+/Ca2+ exchangers: three mammalian gene families control Ca2+ transport. Biochem J, 406, 365-82.

Schnetkamp PP (2004). The SLC24 Na+/Ca2+-K+ exchanger family: vision and beyond. Pflugers Arch, 447, 683-8.

Quednau BD, Philipson KD & Nicoll DA (2004). The sodium/calcium exchanger family-SLC8. Pflugers Arch, 447, 543-8.

Edit history

Date	Action	Author
2009-06-05	Edited	Jassal B
2009-06-05	Authored	Jassal B
2009-06-05	Created	Jassal B
2009-08-24	Reviewed	He L
2022-06-07	Modified	Weiser JD

1 submitted entities found in this pathway, mapping to 1 Reactome entities

Input	UniProt Id
SLC24A3	Q9HC58

21. Presynaptic depolarization and calcium channel opening (R-HSA-112308)

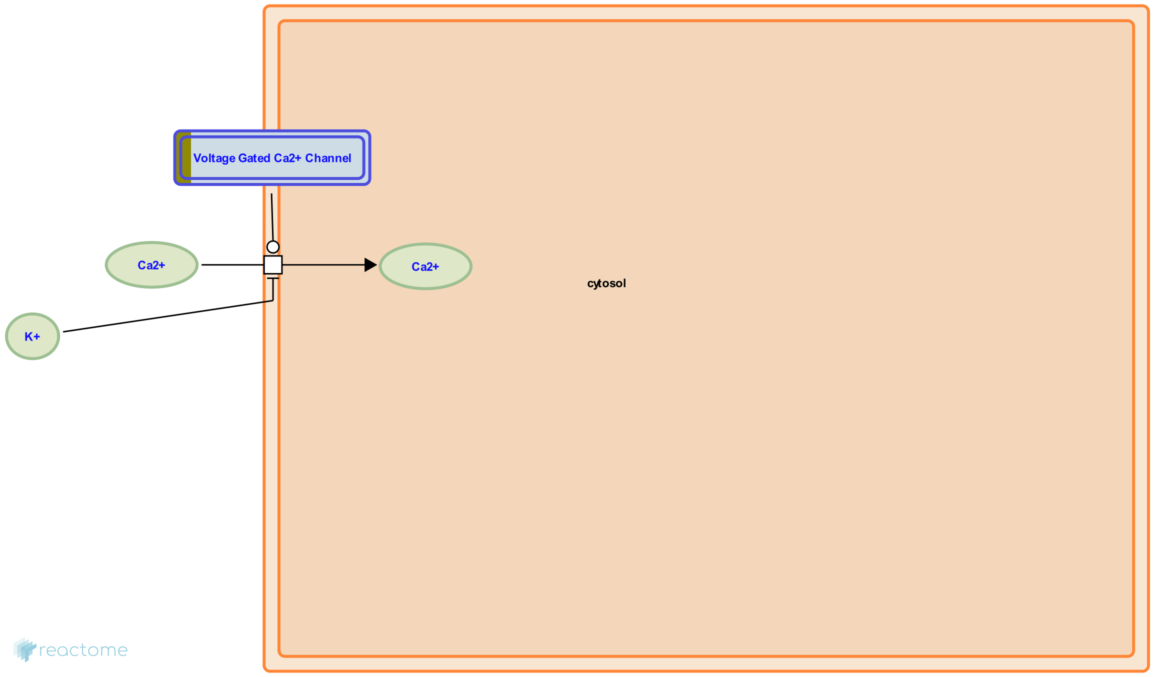

Action potentials occur in electrically excitable cells such as neurons, muscles, and endocrine cells. They are initiated by transient opening of voltage dependent sodium channels, causing a rapid, large depolarization of membrane potentials that spread along the axon membrane.

The action potential travels down the axon and reaches the presynaptic terminal depolarizing the membrane in the pre synaptic terminal. The depolarization causes the voltage gated Ca2+ channels to open allowing the influx of Ca2+ that signals the release of neurotransmitter into the synaptic cleft.

References

Edit history

Date	Action	Author
2004-04-22	Created	Joshi-Tope G
2008-01-14	Edited	Mahajan SS
2008-01-14	Authored	Mahajan SS
2008-04-24	Reviewed	Kavalali E
2020-01-24	Reviewed	Wen H
2022-05-20	Modified	Weiser JD

1 submitted entities found in this pathway, mapping to 1 Reactome entities

Input	UniProt Id
CACNA1A	O00555

22. FGFR1c ligand binding and activation (R-HSA-190373)

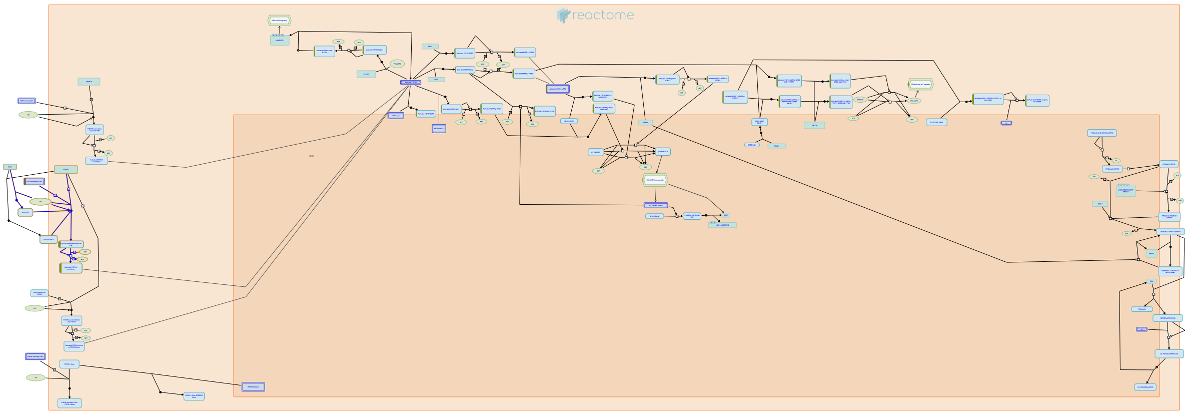

This pathway depicts the binding of an experimentally-verified range of ligands to FGFR1c. While binding affinities may vary considerably within this set, the ligands listed have been established to bring about receptor activation at their reported physiological concentrations.

References

Ornitz DM, Umemori H, Mohammadi M, Olsen SK, Ibrahimi OA & Zhang X (2006). Receptor specificity of the fibroblast growth factor family. The complete mammalian FGF family. J Biol Chem, 281, 15694-700.

Edit history

Date	Action	Author
2007-01-02	Created	de Bono B
2007-01-10	Authored	de Bono B
2007-02-07	Reviewed	Mohammadi M
2016-01-06	Reviewed	Grose RP
2022-05-20	Modified	Weiser JD

1 submitted entities found in this pathway, mapping to 1 Reactome entities

Input	UniProt Id
FGF6	P10767

23. Signaling by activated point mutants of FGFR1 (R-HSA-1839122)

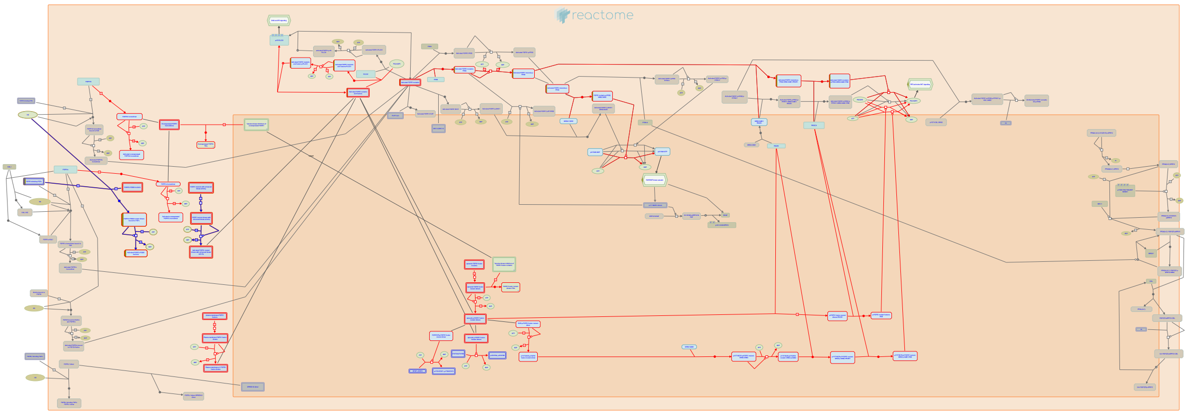

Cellular compartments: plasma membrane, extracellular region, cytosol.

Diseases: cancer, bone development disease.

Unlike FGFR2 and FGFR3, FGFR1 appears not to be a frequent target of activating point mutations (reviewed in Wesche, 2011; Turner and Grose, 2010). Germline point mutations at residue P252 have been identified in Pfeiffer syndrome (reviewed in Webster and Donoghue, 1997; Burke, 1998; Cunningham, 2007) while mutation of the same residue arising somatically has been identified in melanoma and lung cancer (Ruhe, 2007; Davies, 2005). Two kinase domain mutations have been characterized in glioblastoma (Rand, 2005; Network TCGA, 2008), both at positions that are also mutated in an autosomal disorder in one of the FGFR family members (Muenke, 1994; Bellus, 1995a; Bellus, 2000; Tavormina, 1995a; Tavormina, 1999).

References

Cancer Genome Atlas Research Network - (2008). Comprehensive genomic characterization defines human glioblastoma genes and core pathways. Nature, 455, 1061-8.

Donoghue DJ & Webster MK (1997). FGFR activation in skeletal disorders: too much of a good thing. Trends Genet, 13, 178-82.

Wilcox WR, Lachman RS, Cohn DH, Wasmuth JJ, Thompson LM, Wilkin DJ, ... Tavormina PL (1995). Thanatophoric dysplasia (types I and II) caused by distinct mutations in fibroblast growth factor receptor 3. Nat Genet, 9, 321-8.

Easton DF, Dicks E, Stratton MR, Davies H, Cooper CS, Tofts C, ... Varian J (2005). Somatic mutations of the protein kinase gene family in human lung cancer. Cancer Res, 65, 7591-5.

Burke D, Malcolm S, Blundell TL & Wilkes D (1998). Fibroblast growth factor receptors: lessons from the genes. Trends Biochem Sci, 23, 59-62.

Edit history

Date	Action	Author
2011-10-27	Created	Rothfels K
2012-02-10	Authored	Rothfels K
2012-05-15	Reviewed	Ezzat S
2012-05-16	Edited	Rothfels K
2012-05-26	Modified	Rothfels K

1 submitted entities found in this pathway, mapping to 1 Reactome entities

Input	UniProt Id
FGF6	P10767

24. Frs2-mediated activation (R-HSA-170968)

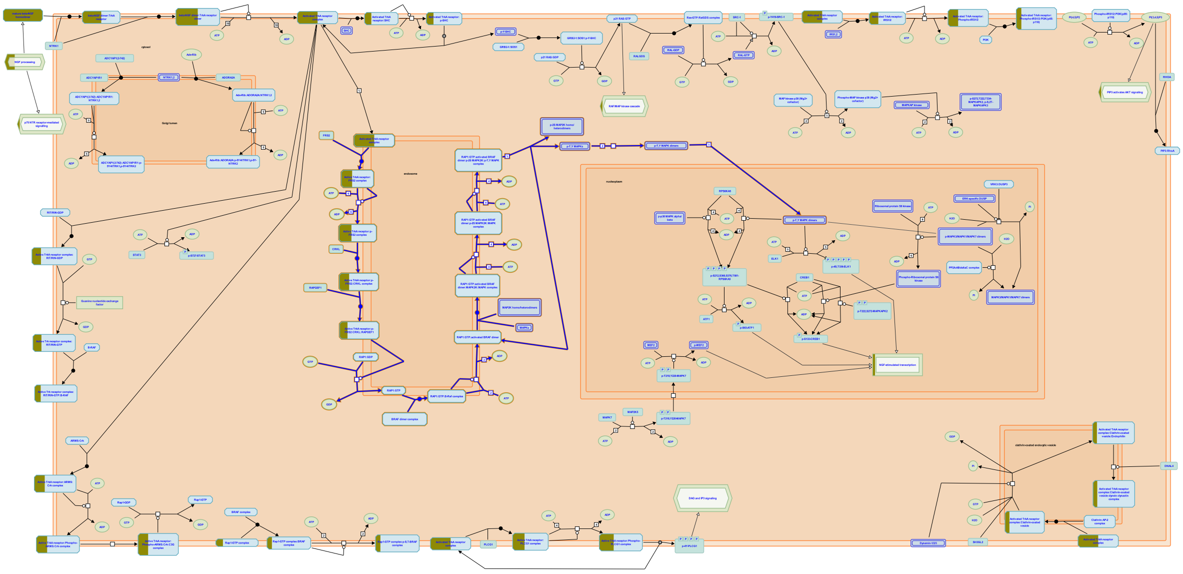

reactome

The adaptor protein Frs2 (Fibroblast growth factor receptor substrate 2) can mediate the prolonged activation of the MAPK (ERK) cascade.

References

Miller FD & Kaplan DR (1997). Signal transduction by the neurotrophin receptors. Curr Opin Cell Biol, 9, 213-21.

Edit history

Date	Action	Author
2006-01-25	Created	Jassal B
2006-10-10	Edited	Jassal B
2006-10-10	Authored	Annibali D, Nasi S
2007-11-08	Reviewed	Greene LA
2022-05-20	Modified	Weiser JD

1 submitted entities found in this pathway, mapping to 1 Reactome entities

Input	UniProt Id
NGF	P01138

25. NF-kB is activated and signals survival (R-HSA-209560)

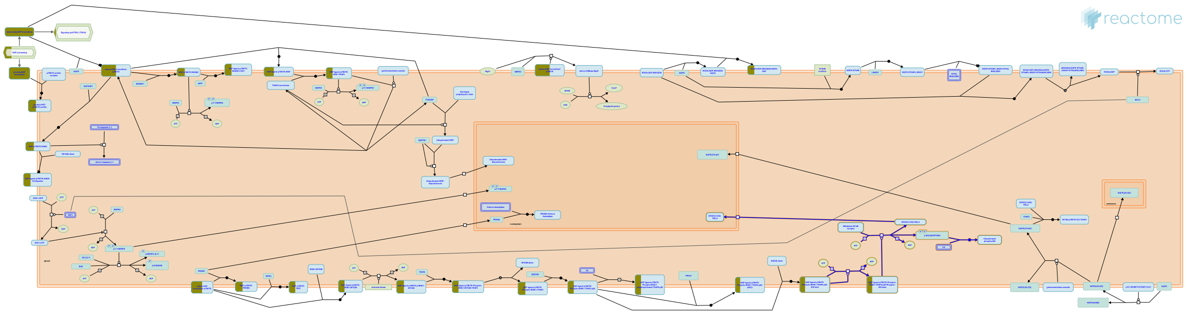

Upon activation in response to NGF, NF-kB moves to the nucleus, where it turns on genes that promote survival, and triggers the expression of HES1/5 to modulate dendritic growth.

References

Mattson MP (2005). NF-kappaB in the survival and plasticity of neurons. Neurochem Res, 30, 883-93 .

Edit history

Date	Action	Author
2006-10-10	Authored	Annibali D, Nasi S
2007-12-19	Created	Jassal B
2008-05-20	Edited	Jassal B
2008-05-20	Reviewed	Friedman WJ
2008-05-28	Reviewed	Chao MV
2022-06-07	Modified	Weiser JD

1 submitted entities found in this pathway, mapping to 1 Reactome entities

Input	UniProt Id
NGF	P01138

6. Identifiers found

Below is a list of the input identifiers that have been found or mapped to an equivalent element in Reactome, classified by resource.

16 of the submitted entities were found, mapping to 20 Reactome entities

Input	UniProt Id	Input	UniProt Id	Input	UniProt Id
ABO	P16442	ADAMTSL4	Q6UY14	CACNA1A	O00555
FGF6	P10767	LRP1	Q13901	LRRK2	Q5S007
MEF2D	Q02078, Q14814	MRPS6	P82932	MRVI1	Q9Y6F6
NGF	P01138	PLCE1	Q9P212	PPCS	Q9HAB8
SCN11A	Q9UI33	SLC24A3	Q9HC58	TRPM8	Q7Z2W7
WDR12	Q9GZL7				

7. Identifiers not found

These 7 identifiers were not found neither mapped to any entity in Reactome.

CFDP1	FHL5	HYDIN	PHACTR1	PRRT2	SUGC2	TSPAN2
